# Comparative assessment of different familial aggregation methods in the context of large and unstructured pedigrees

**DOI:** 10.1093/bioinformatics/bty541

**Published:** 2018-07-13

**Authors:** Christian X Weichenberger, Johannes Rainer, Cristian Pattaro, Peter P Pramstaller, Francisco S Domingues

**Affiliations:** Institute for Biomedicine, Eurac Research, Affiliated Institute of the University of Lübeck, Bolzano, Italy

## Abstract

**Motivation:**

Familial aggregation analysis is an important early step for characterizing the genetic determinants of phenotypes in epidemiological studies. To facilitate this analysis, a collection of methods to detect familial aggregation in large pedigrees has been made available recently. However, efficacy of these methods in real world scenarios remains largely unknown. Here, we assess the performance of five aggregation methods to identify individuals or groups of related individuals affected by a Mendelian trait within a large set of decoys. We investigate method performance under a representative set of combinations of causal variant penetrance, trait prevalence and number of affected generations in the pedigree. These methods are then applied to assess familial aggregation of familial hypercholesterolemia and stroke, in the context of the Cooperative Health Research in South Tyrol (CHRIS) study.

**Results:**

We find that in some situations statistical hypothesis testing with a binomial null distribution achieves performance similar to methods that are based on kinship information, while kinship based methods perform better when information is available on fewer generations. Potential case families from the CHRIS study are reported and the results are discussed taking into account insights from the performance assessment.

**Availability and implementation:**

The familial aggregation analysis package is freely available at the Bioconductor repository, http://www.bioconductor.org/packages/FamAgg.

**Supplementary information:**

Supplementary data are available at *Bioinformatics* online.

## 1 Introduction

Familial aggregation (FA) analysis is classically the first step in genetic epidemiology studies (Khoury *et al.*, 1993). Aim of FA analysis is to identify groups of related individuals carrying the same symptoms or disease due to some underlying shared mechanism. A wide range of diseases has been previously analyzed with FA methods (Naj and Beaty, 2017), including cancer (Tokuhata and Lilienfeld, 1963), cardiovascular disease (Feinleib *et al.*, 1977) and more recently autoimmune disease (Kuo *et al.*, 2015) and insomnia (Jarrin *et al.*, 2017). The increasing availability of large pedigrees for epidemiological research provides both an opportunity and a challenge for FA analysis, especially considering that such large pedigrees are often incomplete and with no regular structure. When pedigrees lack a regular structure, kinship-based methods have proven to be successful in modeling aggregation in terms of distance between individuals (Hill, 1980; Savica *et al.*, 2016; Sveinbjornsdottir *et al.*, 2000). Even though research in this field initiated decades ago, little effort has been made to disseminate aggregation methods and make them available as software tools for use by the scientific community. We therefore have recently developed FamAgg (Rainer *et al.*, 2016), an open source R Bioconductor package providing both established and novel approaches to investigate FA for binary traits in large pedigrees. An exemplary application has demonstrated the potential of kinship-based methods in detecting FA. Nevertheless, a formal comparison between those methods in the context of large and unstructured pedigrees has not yet been made, making it unclear under which scenarios these methods may perform better or worse.

Here, we report on the performance assessment of different kinship-based approaches for FA. Formal comparisons were based on the structure of the pedigrees from the Minnesota Breast Cancer Family study (MBC) (Sellers *et al.*, 1999), which are publicly available and allow reproducible tests. Using such pedigree structure as a backbone, we simulated FA scenarios by generating randomly disease cases in families under a dominant model of inheritance. Methods were then tested for their capability to identify these disease cases in case families among a set of control families with randomly assigned diseased individuals. Finally, we provide applications of the methods to a real context by assessing the aggregation of familial hypercholesterolemia and stroke in the Cooperative Health Research in South Tyrol (CHRIS) study (Pattaro *et al.*, 2015), the former representing a disease that is still largely under-diagnosed and under-treated (Abul-Husn *et al.*, 2016; Nordestgaard *et al.*, 2013).

## 2 Materials and methods

### 2.1 Dataset

The performance assessment analysis is based on the pedigree structure of the publicly available MBC dataset (Sellers *et al.*, 1999), which consists of genealogic information on 426 independent families whose founders entered a longitudinal study on breast cancer in the state of Minnesota (USA) in 1944. After removing 10 060 individuals with no gender information or no relatives (singletons), 18 021 related subjects were available. We further dropped 11 families for different practical reasons, leaving 415 families with 16 719 individuals available for analysis. These families are organized into two to five generations with a median family size of 36 individuals.

### 2.2 Performance assessment

Assessment is set up such that aggregation methods are required to identify families characterized by a Mendelian disease (*case families*) among *control families* with an unspecific random disease background. The choice of a Mendelian trait presents a suitable test setup, as it reflects a real-world scenario where kinship plays an important role among affected individuals.

We create case families in the MBC dataset by running RarePedSim software (Li *et al.*, 2015) to randomly generate disease cases under a Mendelian autosomal dominant model of inheritance (briefly, Mendelian) with full penetrance and no phenocopy effects. The result is at least one affected founder (one individual case) in each case family, who passes on the trait to descendants according to the aforementioned model.

Three parameters are allowed to vary in order to generate a range of scenarios: the disease penetrance (*Q*), the disease prevalence (*R*) and the number of generations (*G*). Penetrance *Q* describes the fraction of cases that also express the phenotype. We test scenarios under *Q *=* *100% (full penetrance), 60% (moderate) and 30% (low). For a case family, penetrance *Q *<* *100%, is achieved by randomly turning (100 − *Q*)% of the affected individuals to non-affected. Prevalence *R* is defined as the simple proportion of disease cases on the total number of subjects in the pedigrees. The term prevalence is used for convenience and it is not referred to any reference population. For control families, we randomly choose affected individuals by simulating a Bernoulli trial with success probabilities *P *=* *1/10 (10%), 1/16 (6.25%), 1/25 (4%), 1/50 (2%) and 1/80 (1.25%), such that the proportion of affected individuals in the overall population corresponds to prevalence *R *=* P*. Ultimately, the third parameter controls the number of generations (*G*) that are affected by the Mendelian trait, and mimics a spontaneous mutation or a mutation that is introduced into the family by marriage. We start with a case family as generated by RarePedSim, and keep the disease status of a sub-branch with *G* generations. For the remaining family members the disease status is cleared and reassigned according to the prevalence model described before. This procedure is carried out for *G *=* *2 and *G *=* *3 generations. When investigating all generations of a case family (*G* = all), we transfer the affected individuals as output by RarePedSim without masking any individuals. Table 1 summarizes the parameter space covered by the performance assessment.

**Table 1.** Number of generations used in the performance assessment for each penetrance/prevalence pair

	Penetrance (*Q*)		
Prevalence (*R*)	100% (full)	60% (moderate)	30% (low)
10%	3/all	3/all	—^a^
6.25%	3/all	3/all	—
4%	2/3/all	2/3/all	2/3/all
2%	2/3/all	2/3/all	2/3/all
1.25%	2/3/all	2/3/all	—

a No assessment was performed for penetrance/prevalence pairs indicated by a dash.

Generally, the assessment comprises of a case family that carries a Mendelian trait and control families. We therefore define a family set *F_a_* to consist of all 415 families where family number *a* (1 ≤ *a* ≤ 415) is the case family and all other families *b* ≠ *a* are controls. In total, we obtain 415 family sets *F_i_*, each with one case family and 414 control families. Case families are further parameterized by the number of affected generations *G* and causal variant penetrance *Q*, whereas affected individuals in controls are generated according to trait prevalence *R* described above. We note that for each possible parameter combination *G*, *Q* and *R*, the controls of family set *F_a_* are generated independently and randomly.

### 2.3 Familial aggregation analysis

The assessment evaluates the performance of several tests for FA without stratification provided by the FamAgg Bioconductor package (Rainer *et al.*, 2016), which utilize the kinship coefficient (Malécot, 1948), a measure that quantifies relationship between two individuals in a pedigree by computing the probability that an allele is shared identical-by-descent at a given locus. These methods have been previously described in detail (Rainer *et al.*, 2016). Briefly, each method calculates a specific test statistic from the observed family data, and a sampling distribution under the null hypothesis is generated empirically by random sampling 50 000 times the same number of affected individuals from the pool of all 415 families. The genealogical index of familiality (IF) test calculates the mean kinship coefficient between all affected individuals of a family (Hill, 1980). For an individual *i*, the kinship sum (KS) test computes the sum of kinship coefficients for all other affected family members. Kinship group tests utilize the most distant relative of a specific affected individual *i* to define a group of individuals more closely related to the affected individual. The group ratio (GR) test simply counts the number of affected persons in that group, whereas the group closest relative (GC) test records the kinship coefficient of the closest affected relative in the group. We have added a binomial test (PB) that estimates the probability to detect by chance a family of size *n* with ≥*k* affected members given trait prevalence *R*, i.e. we perform hypothesis testing with a random variable *X* ∼ B(*n*, *R*). This binomial test has now been integrated into the FamAgg package. Tests are assessed on their ability to identify the case family based on computed *P* values. This can readily be done for the IF and PB tests, which report *P* values on families only. For the KS, GR and GC tests that report a *P* value for each affected individual, we take the lowest *P* value within a family. We adjust for multiple hypothesis testing employing Benjamini-Hochberg correction at a false discovery rate (FDR) of 0.05. We notice that distinct unadjusted *P* values may cluster within the same FDR level. Therefore, unless otherwise noted, ranking is performed on unadjusted *P* values to preserve order and avoid ties. In addition, significance is reported at the aforementioned FDR. We use the notations *P*_raw_ and *P*_adj_ for unadjusted and adjusted *P* values, respectively.

### 2.4 Performance measures

For evaluation of a given aggregation method with fixed parameters *G*, *Q* and *R*, results obtained for all 415 family sets are combined, giving rise to 415 cases and 415 × 414 = 171 810 controls. Given a *P* value cutoff *t*, a binary classification is established based on the knowledge of cases and controls, defining true positives (TP) and false negatives (FN) (cases with *P* value ≤ *t* and > *t*, respectively), and false positives and true negatives (controls with *P* value ≤ *t* and > *t*, respectively) (Baldi *et al.*, 2000). Since this scenario defines a heavily imbalanced dataset with on average one case in 415 families, we analyze test performance by precision/recall curves, which put emphasis on detectability of true positives (Davis and Goadrich, 2006). These two performance measures are defined as follows: precision = TP/(TP + FP) and recall = TP/(TP + FN), whereas precision is also known as the positive predictive value, and recall as the true positive rate or sensitivity.

### 2.5 Application to the CHRIS study

We apply FA tests to data from the CHRIS study, a population-based study carried out in an alpine Italian valley (Pattaro *et al.*, 2015). The recruitment area includes several municipalities, each one characterized by a main center and small villages and settlements. Recruitment is performed municipality-wise, and the pedigree structure reflects the geographical landscape of the area. All participants underwent blood drawing following overnight fasting. Medical history was reconstructed via standardized computer assisted questionnaires. Drugs used for treatment were barcode scanned and classified according to the Anatomical Therapeutic Chemical (ATC) classification code. The study received ethical approval by the Ethical Committee of the Healthcare System of the Autonomous Province of Bolzano. All participants gave written informed consent.

After removing singletons, the dataset includes 4373 phenotyped individuals who reported two generations of ancestors resulting in 186 pedigrees with a total of 9024 individuals, out of which 4651 remain unphenotyped, but are used for establishing kinship (Noce *et al.*, 2017). One exceptionally large family (termed ‘XL’) includes 3676 phenotyped and 3997 unphenotyped individuals. However, this family’s tree exhibits a highly horizontal structure with the furthest two individuals being separated by 65 individuals along the shortest path within only five generations. Excluding family XL, family sizes range between 3 and 45 members.

Pedigree information is used to identify familial clusters of hypercholesterolemia (HC), a condition with high serum levels of total cholesterol or low density lipoprotein (LDL), which presents a risk factor for arteriosclerosis and development of coronary heart disease (Carmena *et al.*, 2004). We define HC as either total cholesterol level >290 mg/dl or low-density lipoprotein cholesterol (LDL cholesterol) level >190 mg/dl (Bertolini *et al.*, 2017; Simon Broome Register Group, 1991), provided that triglyceride levels are <200 mg/dl (National Heart Lung and Blood Institute, 2002). We identify 296 HC cases out of 4979, corresponding to a disease prevalence of *R *=* *5.94%. When removing singletons, we find 265 affected out of 4373, which gives *R *=* *6.06%. Information on prescription of statins (ATC classification code C10) is not considered a case of HC, but is used for descriptive purposes during family analysis.

In the CHRIS study, stroke is assessed based on the Jackson Heart Study (Sempos *et al.*, 1999) screening questionnaire. We utilize here the question ‘Have you ever been told by a doctor that you had a stroke?’, resulting in 54 cases of self-reported incidences of stroke (*R *=* *1.23%) (see Section 5 of the Supplementary Material for more details.).

## 3 Results

### 3.1 Performance assessment

Each FA test was applied to a set of 415 families, where one particular family (case family) included affected individuals following an autosomal dominant inheritance mode. Each family was assigned as case family and combined with the remaining 414 control families, resulting in 415 family sets. Case families were parameterized by trait penetrance *Q* and number of affected generations *G*, and controls were generated according to prevalence *R*. Aggregation tests were challenged to identify cases in 415 family sets for an extensive combination of parameters *G*, *Q* and *R*. Certain parameter combinations were not examined, as they either resulted in cases indistinguishable from the background (e.g. high prevalence, low penetrance and two affected generations) or represented trivial cases (see Table 1).

#### *3.1.1* Assessment by family set

Two questions need to be addressed when analyzing the performance of an aggregation test for a given family set: first, what is the rank of the case family and second, is the reported case statistically significant? As mentioned in the Materials and methods section, ranking is performed on unadjusted *P* values, and significance is reported at a FDR of 0.05.

**Fig. 1.**
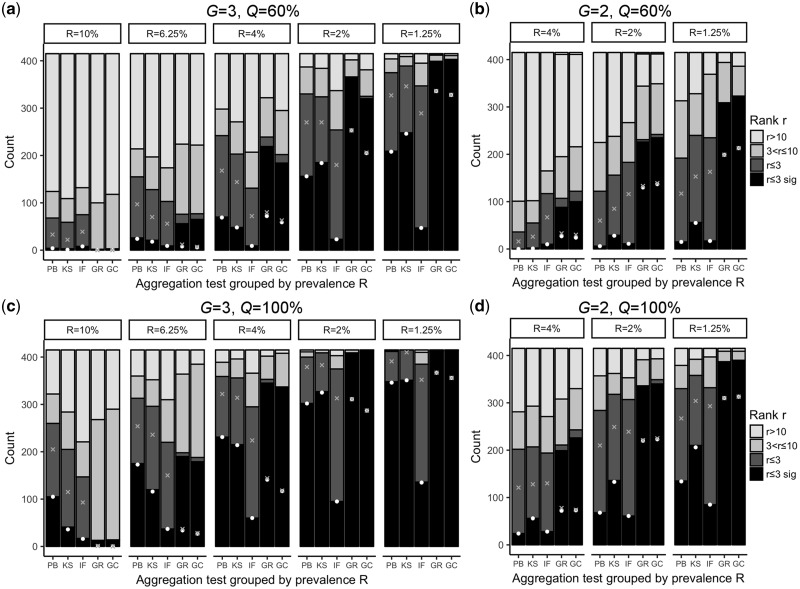
Number of case families by test rank and prevalence. For a fixed number of generations *G* and a fixed penetrance *Q*, each panel of this figure shows the number of cases a test has found for different rank ranges. Additionally, panels are further divided into groups describing prevalence *R*, ordered from highest prevalence (left) to lowest (right). Each such prevalence group then visualizes the number of cases a test has detected for a certain rank range by bar heights. The ranges partition the set of 415 cases and the associated bars are colored as follows: black, case is a significant hit in the top three; dark gray, case ranks in the top three, but is insignificant; gray, case ranks between four and 10, independent of significance; light gray, case ranks higher than 10. A white point is positioned at a test’s number of top scoring significant cases and a gray cross stands for the number of overall top scoring cases. (**a**) Three generations and 60% penetrance. Generally, tests perform better with decreasing trait prevalence *R*, as cases become easier distinguishable from controls due to lower background noise. We point out that IF test sticks out with the lowest number of significant top and top three hits. For *R *≤* *4%, tests GC and GR have the highest number of significant top three ranking cases. (**b**) Two generations and 60% penetrance. Notice that no assessment was carried out for prevalence *R *=* *10% and *R *=* *6.25%, due to excess background noise generated by the controls. Spreading a trait across multiple generations increases test performance, as becomes evident when comparing the results from two affected generations (this panel) with those obtained by three [panel (a)]. (**c**) Three generations and full penetrance. A notable boost of test performance is observed when traits are switched from moderately to fully penetrant [panel (a) versus this panel]. Interestingly, PB test outperforms KS test for high prevalence settings *R *=* *10% and *R *=* *6.25%. (**d**) Two generations and full penetrance

Figure 1 shows the assessment results for moderately and fully penetrant traits restricted to two and three generations. Generally, for moderate penetrance and high prevalence scenarios the aggregation tests are operating in the limit of detectability (Fig. 1a and b). For fully penetrant scenarios the performance of the different methods improves considerably (Fig. 1c and d), as expressed by a manifold increase of top three ranking cases (black and dark gray bars in Fig. 1). Furthermore, the performance is lower for *G *=* *2 than when three generations are considered (Fig. 1b and d versus a and c). Tests PB and KS were frequently the best performing methods regarding the number of top ranking case families (Fig. 1, crosses and dots). For a more challenging *G *=* *2, KS test had an advantage over PB test. It also turned out that GC and GR tests reported a high number of significant cases among top three results, but usually they result in no further cases among the insignificant top three hits. However, for other parameter combinations the number of top three ranking case families found by GC and GR tests were similar to those identified by KS and PB tests. (See Supplementary Figures S1–S9 for all parameter sets.) We note in advance that despite their good performance in ranking case families in the top three, tests GC and GR are characterized by a high number of false positives, such that results should be treated cautiously. A detailed analysis of cases differentiated by rank, significance and number of affected individuals is given by Supplementary Figures S10–S41.

The overlap of top ranking cases among the different methods is provided in Figure 2. The agreement is lower for higher prevalence (*R *=* *6.25%), with a considerable number of unique top rank cases identified by PB, followed by IF and KS. The agreement between methods increased for lower prevalence (*R *=* *2%). Overall, with decreasing difficulty, expressed by higher *G*, lower *R* or higher *Q*, we observed higher agreement in ranking cases on the top (we refer to Supplementary Figures S42–S73 for a comprehensive set of overlap analysis plots).

**Fig. 2.**
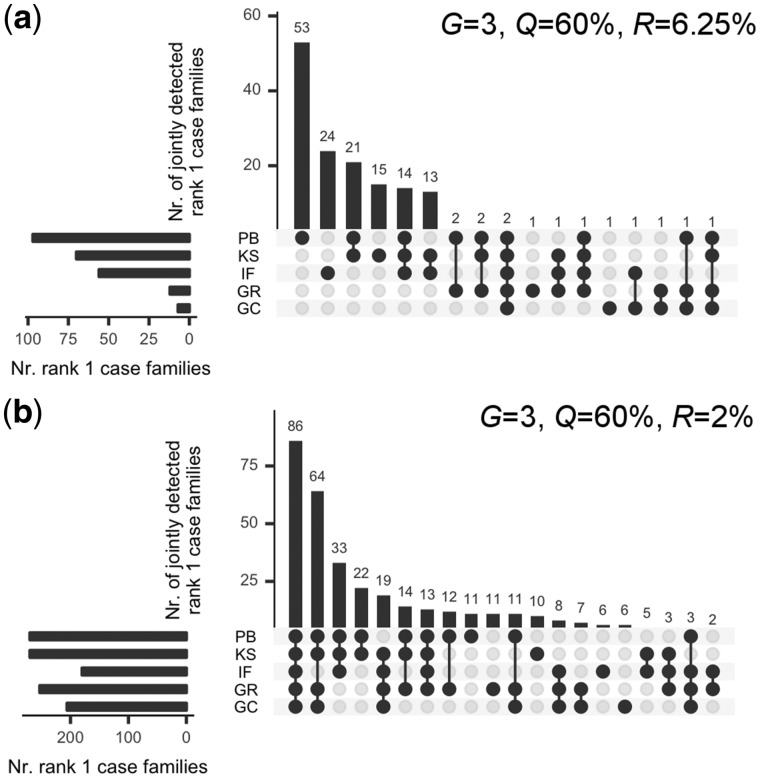
Top rank case family overlaps via UpSet plots. Each plot visualizes the overlap of rank 1 case families between the five aggregation methods, independent of significance. The diagrams show on the lower left hand side the set size for rank 1 case families for each method, one per row. The central element is a dot matrix, and filled dots connected by lines indicate the sets that are overlapping, and the magnitude of the overlap is shown in the bar plot above. A single dot without any connecting lines refers to those elements of a set that do not overlap with any other set. The plots are limited to 20 overlaps out of 2^5^–1 = 31 possible overlaps and are sorted by overlap size. Plots were generated with UpSetR (Conway *et al.*, 2017). (**a**) Overlaps for three generations, 60% penetrance, and 6.25% prevalence. PB test identifies 53 families that no other test has put on the top rank. Tests IF and KS also show some ability to uniquely place cases on top. Overall, 140 out of 415 cases (34%) were identified by tests PB, KS or IF. (**b**) Same parameters as in panel (a), but prevalence *R *=* *2%. Lowering the trait prevalence, the five tests agree for 86 case families (21%) by ranking them on the top. Another 64 cases are top ranked jointly by tests PB, KS, GC and GR. There is high agreement between methods, and compared to the scenario presented in panel (a), no single method is able to distinguish itself from the other aggregation tests

#### *3.1.2* Assessment by binary classifier

**Fig. 3.**
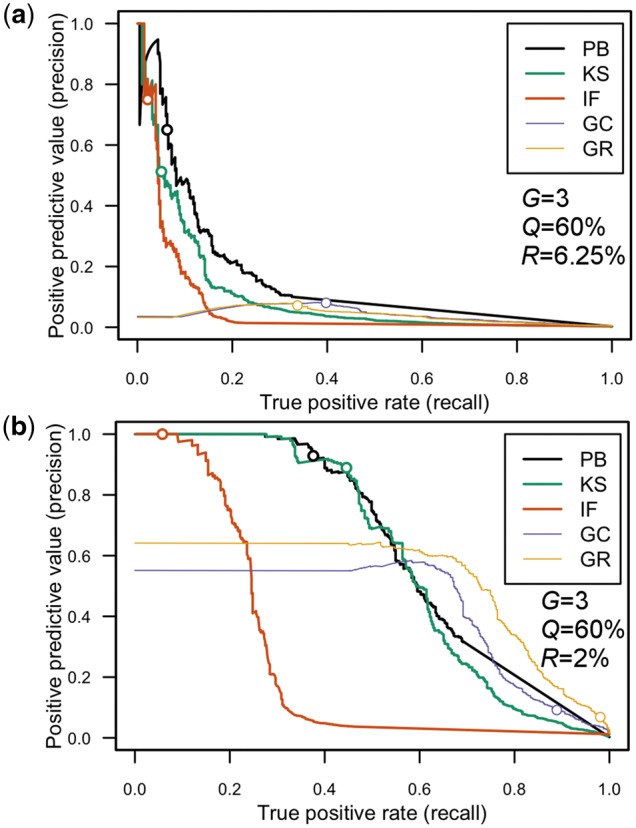
Binary classification performance evaluation by precision/recall plots. The binary classifier curves show precision (*y*-axis) versus recall (*x*-axis) values based on *P*_adj_ for selected parameters *G*, *Q* and *R*. An ideal curve would remain at precision 1.0 till it reaches the recall value of 1.0 and then drop in a single step to zero. Since all plots are based on 415 positives (the cases), the *x*-axis can also be interpreted on an absolute scale, e.g. a recall of 0.4 corresponds to 0.4 × 415 = 166 cases. The recall/precision pair where *P*_adj_ = 0.05 is marked by a circle. Tests are denoted by color and line thickness as given by the figure legends. Panels shown here correspond to data also presented in Figures 1 and 2. (**a** and **b**) Parameters as in Figure 2a and 2b, respectively. We refer to Supplementary Material Section 4 for a detailed discussion on the shape of the curves

In the previous section, we have compared aggregation tests by investigating the rank of the affected case family within a family set in combination with *P*_adj_ ≤ 0.05 cutoff for assessing significant aggregation. In a second analysis, we have formulated the question of overall test performance as a binary classification problem based on *P*_adj_, where for a fixed parameter set *G*, *Q* and *R* all 415 family sets were treated as a single set with 415 cases and 415 × 414 controls. For selected parameters, Figure 3 summarizes the results by means of precision/recall curves where threshold *P*_adj_ = 0.05 is marked by a circle. The performance curves for the GC and GR tests start with precision value well below 1.0 and remain at constant precision until a certain recall value. This is due to a mixture of cases and controls that both were reported with the same, small *P*_raw_ value, implying that these two tests report false positives even at moderate recall and high significance level. Frequently, we observe a short recovery phase indicated by increased precision, during which many cases are detected (e.g. Fig. 3a). After reaching a local maximum, precision drops due to an excess of false positives.

The PB and KS methods stood out by performing consistently better than the other three tests regarding precision and recall. Noteworthy, even though at low recall, tests PB, KS and IF are characterized by reasonably high precision values at the significance cutoff of *P*_adj_ = 0.05, distinguishing them as acceptable methods for FA analysis under the scenarios evaluated. The IF test usually lacks performance when compared to PB and KS tests, but showed some advantages when applied to small subfamilies with two generations or low to moderate trait penetrance (cf. Supplementary Figures S75–S77, S82, S85 and S96).

### 3.2 Familial aggregation in the CHRIS population study

We have applied different methods to detect FA to the CHRIS study in order to identify potential cases of familial hypercholesterolemia and stroke. We note that CHRIS pedigrees are characterized by nuclear families that were joined by a varying number of non-participating parents and grandparents.

#### *3.2.1* Detection of familial cases of hypercholesterolemia

In the CHRIS study, we found the HC threshold levels (defined in Materials and methods) of total cholesterol, LDL cholesterol, and triglyceride as the 97th, 93rd, and 94th percentile, respectively. Overall, applying the proposed HC criteria resulted in a disease prevalence *R* of 5.94% in the study.

Due to the large family XL, analyzing the CHRIS pedigrees represented a challenge, especially for methods based on families rather than individuals (IF and PB), as they might not have been able to fully capture the finer inheritance patterns between affected participants within that family. Family XL contained 236 out of all 265 affected participants. Interestingly, this family was identified by the IF test with *P*_adj_ = 1.41 × 10^−3^. The PB test did not report any significant family (*P*_raw_(family XL) = 0.18), which may be indicative that the proportion of affected individuals found in family XL is in line with the overall disease prevalence in the cohort. One the other hand, tests GR and GS resulted in 26 and 27 significant groups of individuals, respectively. Ultimately, the KS test did not report any significant results, but 11 participants were found as borderline cases with *P*_adj_ = 0.0528, all of them belonging to family XL. These 11 individuals form five distinct groups of up to nine affected relatives sharing kinship. Remarkably, all these groups also showed up in the top nine and top 15 hits reported by GC and GR tests, respectively (Table 2). Each of these groups can be seen as a separate family, since affected members do not share kinship between the groups. Therefore, we computed the *P*_raw_ values based on the number of phenotyped and affected individuals just as they would be obtained when applying the PB test to the group (see Methods). Remarkably, the ranking by these *P*_raw_ values is identical to the order obtained by KS test.

**Table 2.** Top scoring family groups for HC with associated results of familial aggregation tests

				KS^a^		GC			GR			PB
Group^b^	*N*^c^	*N*_kaff_^d^	*N*_aff_^e^	*P*_raw_	Rank^f^	*P*_raw_	*P*_adj_	Rank	*P*_raw_	*P*_adj_	Rank	*P*_raw_^g^
A	20	5	5	2.00 × 10^−4^	1	1.50 × 10^−4^	2.61 × 10^−3^	5	1.40 × 10^−4^	3.04 × 10^−3^	4	5.88 × 10^−3^
B	67	8	9	5.23 × 10^−4^	2	1.55 × 10^−3^	1.69 × 10^−2^	8	6.60 × 10^−4^	8.20 × 10^−3^	7	1.95 × 10^−2^
C	63	7	8	2.05 × 10^−3^	6.5	1.18 × 10^−3^	1.47 × 10^−2^	7	1.80 × 10^−4^	3.13 × 10^−3^	5	3.60 × 10^−2^
D	82	6	8	2.05 × 10^−3^	6.5	1.87 × 10^−3^	1.81 × 10^−2^	9	1.30 × 10^−4^	3.04 × 10^−3^	3	1.24 × 10^−1^
E	114	9	10	2.19 × 10^−3^	10	2.70 × 10^−4^	3.92 × 10^−3^	6	5.80 × 10^−3^	3.36 × 10^−2^	15	1.54 × 10^−1^

a Table is sorted by *P*_raw_ of KS test and refers to all individuals with *P*_adj_ = 0.0528 (the best *P*_adj_ observed for KS test).

b Isolated group of individuals within family XL that maximizes the number of affected people sharing kinship.

c Number of phenotyped individuals in the group.

d Number of affected individuals that share kinship within the group.

e Overall number of affected individuals in the group, irrespective of kinship.

f Rank denotes the position in the specific aggregation test result list. In case of ties, ranks are averaged.

g Result as would be returned by the PB test for a family with *N* members and *N*_aff_ affected family members and Bernoulli trail success probability *P *=* *265/4373, which is the prevalence of HC in the CHRIS study excluding singletons.

**Fig. 4.**
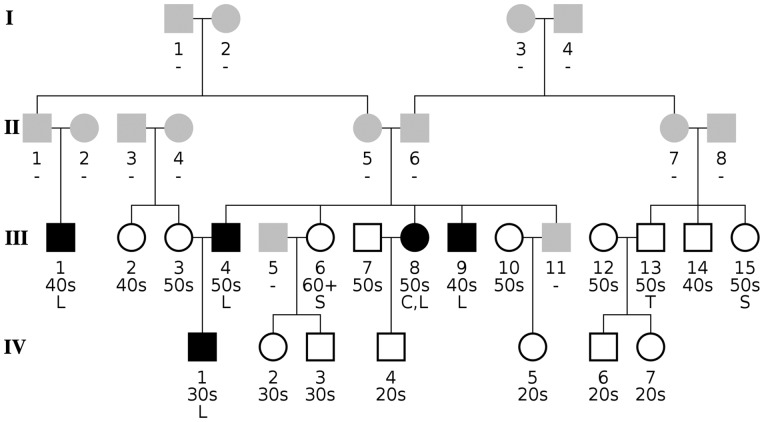
Family tree of group A (part of family XL). The pedigree has been constructed to include the complete sibship plus spouses of all five affected members (filled black symbols). Known non-participating relatives are denoted by gray symbols, whereas unaffected participants are indicated by empty symbols. Below each individual we list up to three lines: the individual’s integer identifier; an age range specifying the decade of the individual’s age at participation (e.g. 30s for thirties, ranging from 30 to 39 years), or a hyphen (‘-’) if the age is unknown; and a code detailing the participant’s phenotype: C, high total cholesterol level; L, high LDL level; S, prescription of statins (ATC code C10); T, high triglyceride level. Most affected participants are related to individuals II.5 and II.6 either as parents or grandparents, and individual III.6 is on statin medication. Male III.13 has high triglyceride levels but is otherwise unaffected by HC, however his sister III.15 is treated with statins

Table 2 shows that family group A has 20 members, five of them were affected by HC. The remaining groups B to E were at least three times as large and have only a few more affected individuals, which are rather spread out across the pedigrees and are therefore less likely to be carrier of fHC. Family group A, shown in Figure 4, revealed the most compact pedigree. The oldest two generations I and II were not phenotyped, as they only were reported by the participants, but helped in connecting the family members. Three out of the five siblings with parents II.5 and II.6 are affected, where one of the unaffected is taking statins (sister III.6) and the other unaffected (III.11) did not participate in the study. One young offspring of these siblings is also affected (male IV.1), whereas the others are unaffected. No cases of HC are found in the sub-pedigree formed by the parents of female III.3, indicating that this part of the family does not contribute to the affected male IV.1. Individual III.1 represents the fifth case in this family group and belongs to the maternal part of the family formed by parents II.5 and 6. On the paternal side we observe one individual under statin therapy (female III.15) and her brother with high triglyceride levels. Taken together, this group of affected individuals represents a possible candidate for fHC within the CHRIS dataset. When relating this result to our assessment, we found prevalence *R *=* *6.25% and penetrance *Q *=* *60% limited to three generations an appropriate parameter set reflecting the fHC scenario. At the significance level of *P*_adj_ = 0.0528 for the KS assessment, we observed with these parameters a precision of 0.51 (cf. Fig. 3a).

#### *3.2.2* Detection of familial cases of stroke

When performing FA analysis of stroke within CHRIS study participants, we have identified a nuclear family consisting of an affected mother (age group 80s) and both of her sons (age group 60s), all affected by stroke. In the performance assessment, this situation is best described by a fully penetrant trait in two generations with disease prevalence *R *=* *1.25% (cf. Fig. 1d). All kinship-based methods identified this trio as a significant cluster (*P*_adj_ < 0.05). Section 5 of the Supplementary Material provides further information that this family exhibits a high level of stroke aggregation.

## 4 Discussion

We have completed a performance assessment in order to investigate the ability of different methods to detect trait aggregation in large families, covering a range of trait prevalence and penetrance parameters, as well as the number of affected generations for a given set of pedigrees with phenotyped individuals. Overall, the PB test consistently displays high performance. Remarkably, this test was able to rank on top more cases for more challenging high trait prevalence (*R *=* *10% and *R *=* *6.25%) scenarios than the other tests based on kinship. Conversely, the PB test was inferior to KS for incomplete penetrance and very low prevalence (*R *≤* *2%) in up to three affected generations. Under such circumstances, kinship-based tests were more selective in detecting cases. In several scenarios with only two affected generations the IF test has demonstrated small advantages. For low and moderate penetrance, the KS test performed slightly better than the PB test. The ability of KS to identify individual cases in arbitrarily large families (in contrast to PB and IF), makes it a very good all-round aggregation test. Tests GC and GR clearly overestimate the significance of reported results, and should only be taken as an indicator to support hypothesis building based on the other tests’ results.

By looking at the overlap of the methods’ top ranking cases (Fig. 3, Supplementary Figures S42–S73), we identified several tendencies. First, tests GC and GR tend to report redundant results, as they frequently reported the same cases on top. Second, for low prevalence (*R *≤* *4%), there was considerable agreement between the different tests. Most importantly however, for many parameter combinations, PB is the only test being able to identify many cases. Therefore, the current best strategy is to combine the sensitivity of PB with the kinship awareness of KS, and which should inspire future development of more powerful aggregation methods.

Analysis of the performance assessment suggests a strategy to efficiently exploit the advantages of each aggregation test, as we have demonstrated in the fHC and stroke examples. The results indicate that it is advisable to run all tests from the FamAgg package and compare the top ranking families. Significance is assigned by choosing a false discovery rate threshold *P*_adj_, preferably ≤0.05, since this allows to better interpret the findings by comparison with the performance assessment results. The higher the number of agreement between tests PB, KS and IF, the higher the confidence that can be put on the findings. The KS test should always list the family in question as top or very high ranking, and if the family size is not too large, the PB test should confirm the KS results. Hits also detected by the IF test are further strengthening the overall analysis, especially when they are significant at a false discovery rate ≤0.05, since this test showed very high precision at this level of significance. (The IF test reported significant clustering of stroke and fHC cases in family XL.) On the other hand, due to the low precision of GC and GR aggregation methods, a positive hit from these tests should only be considered supportive for other tests’ results.

It should be recalled that in the performance assessment we observe many large families (median family size is 36 members) with all generations phenotyped, whereas the CHRIS study contains one distinguished family (family XL) that constitutes 84% of all 4373 phenotyped individuals and a set of smaller families (median family size 6). Furthermore, half of the individuals in the CHRIS pedigrees are not phenotyped, as they have been reported as ancestors of participants. Typically, these are the oldest two generations and are of great aid for establishing remote relationships between participants, but they also hamper a direct comparison with the performance assessment, which is based on fully phenotyped pedigree data. In the reported case of familial stroke, the comparison could be made directly. However, in the fHC example, we have taken a compromise and compared to a scenario with three affected generations, even though the grandparents remained unphenotyped. We find this an acceptable approach, especially since the disease was modeled as not fully penetrant. Affected individuals outside this sub-pedigree are then considered non-familial cases of HC, in agreement with the model introduced in the performance assessment. We are planning to further investigate the identified candidate family by examining the affected members for potential causal variants of fHC (Dron and Hegele, 2016).

## Funding

The research and the CHRIS study were funded by the Department of Innovation, Research, Development and Cooperatives of the Autonomous Province of Bolzano-South Tyrol. The authors thank the Department of Innovation, Research and University of the Autonomous Province of Bozen/Bolzano for covering the Open Access publication costs.

*Conflict of Interest*: none declared.

## Supplementary Material

Supplementary Data
