# Supplementary material for: Comparative assessment of different familial aggregation methods in the context of large and unstructured pedigrees
Source: Bioinformatics. 2018 Jul 13;35(1):69–76. doi: 10.1093/bioinformatics/bty541 (PMC6298062; doi:10.1093/bioinformatics/bty541)
Supplement: Supplementary Data [file bty541_suppl_data.zip › supplementary-information.pdf]

Supplementary Information

Contents

1 Number of case families by test rank and prevalence 1

2 Rank of case families and associated number of affected family members 5

3 Test overlap of top ranking case families 22

4 Binary classification performance evaluation 39

5 Investigation of familial cases of stroke in CHRIS 56

---

1 Number of case families by test rank and prevalence

This section lists plots as presented in Figure 1 for all parameters of the performance assessment. This includes two, three, or all generations and combinations with trait penetrance and prevalence according to Table 1.

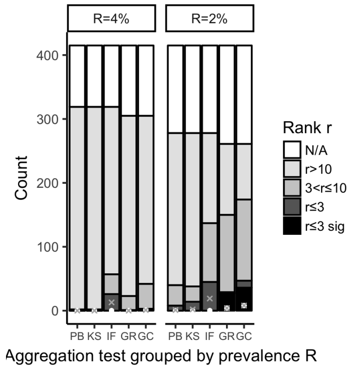

Supplementary Figure S1: Number of affected generations $G = 2$, penetrance $Q = 30\%$. (See Figure 1 for a detailed description.)

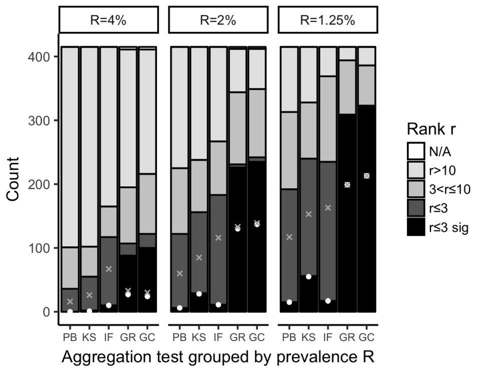

Supplementary Figure S2: Number of affected generations $G = 2$, penetrance $Q = 60\%$. (See Figure 1 for a detailed description.)

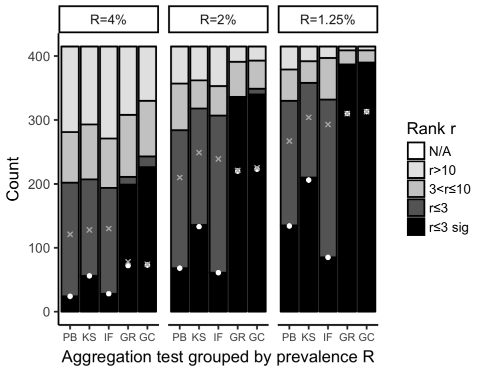

Supplementary Figure S3: Number of affected generations $G = 2$, penetrance $Q = 100\%$. (See Figure 1 for a detailed description.)

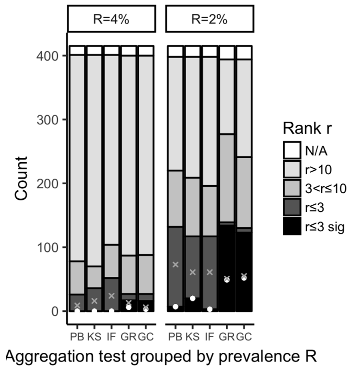

Supplementary Figure S4: Number of affected generations $G = 3$, penetrance $Q = 30\%$. (See Figure 1 for a detailed description.)

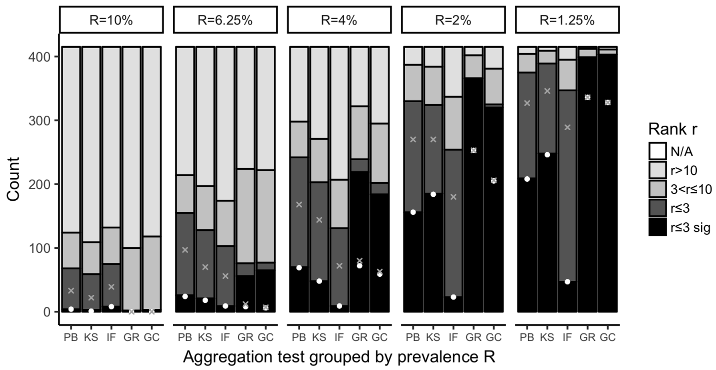

Supplementary Figure S5: Number of affected generations $G = 3$, penetrance $Q = 60\%$. (See Figure 1 for a detailed description.)

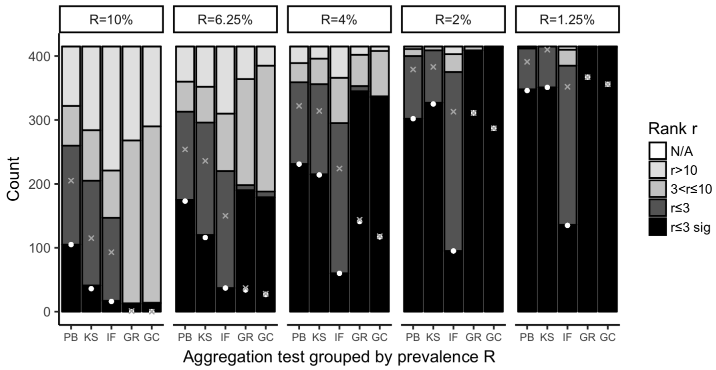

Supplementary Figure S6: Number of affected generations $G = 3$, penetrance $Q = 100\%$. (See Figure 1 for a detailed description.)

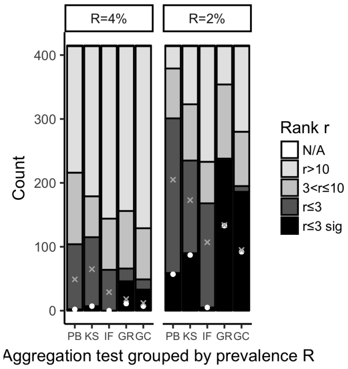

Supplementary Figure S7: Number of affected generations $G =$ all, penetrance $Q = 30\%$. (See Figure 1 for a detailed description.)

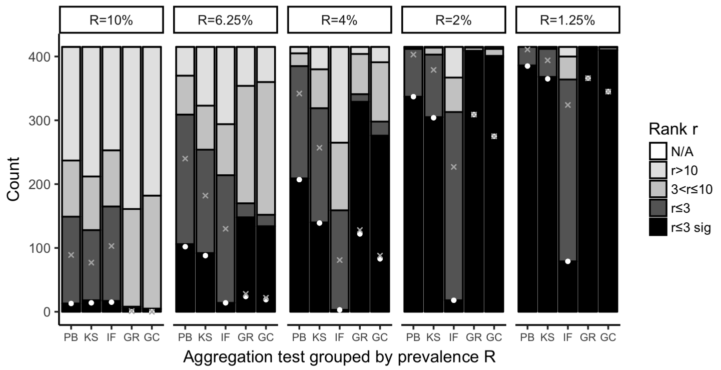

Supplementary Figure S8: Number of affected generations $G = \text{all}$, penetrance $Q = 60\%$. (See Figure 1 for a detailed description.)

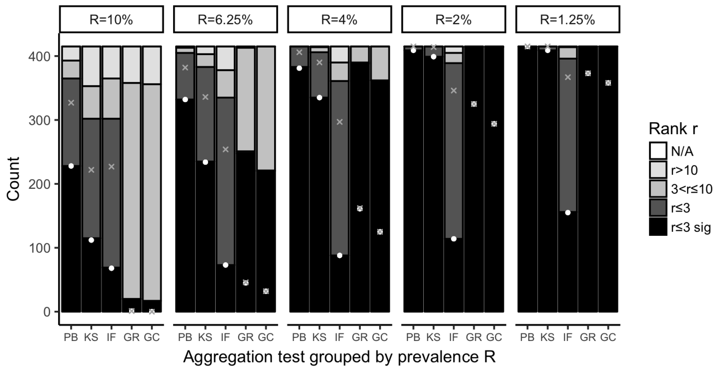

Supplementary Figure S9: Number of affected generations $G = \text{all}$, penetrance $Q = 100\%$. (See Figure 1 for a detailed description.)

2 Rank of case families and associated number of affected family members

This section provides a detailed analysis of ranked cases differentiated by rank, significance, and number of affected individuals. As in the previous section, we analyze a test's ability to detect and rank a case family and summarize the result over the 415 family sets.

Each of the following figures presents a detailed view on the rank distribution for data shown in in the previous section. Essentially, we give a breakup for each bar of the plots listed there. For a fixed set of parameters G, Q, and R, ranks of the case family in each of the 415 family sets are plotted as dots and circles on a logarithmic scale in order to highlight top ranking cases. A dot indicates a significant case, whereas a circle stands for not significant. Symbols are colored by the number of affected family members as shown in the legend. Cases are plotted from left to right by ascending rank, and within the same rank cases are assembled into groups of affected members, first significant, then insignificant ones, ordered descending by the number of affected family members.

In Fig. 1 of the main text we have chosen several non-trivial examples where aggregation tests exhibited varying performance, which are further discussed here. Assuming three affected generations and moderate penetrance ($G = 3$, $Q = 60\%$), the methods were incapable to place cases with up to three affected individuals among the top three ranks (Suppl. Fig. S21), which noticeably improved on a fully penetrant setting (Suppl. Fig. S26). The number of affected individuals appears to be most influential on KS test, especially when compared to PB test. For $R = 2\%$, we found more than half of the top ranking cases to be significant (Suppl. Fig. S23). Even though GR test was able to rank cases among the top (Suppl. Fig. S23), we observe an excess of families reported with significant aggregation, which negatively impacts precision. The same holds for GC test.

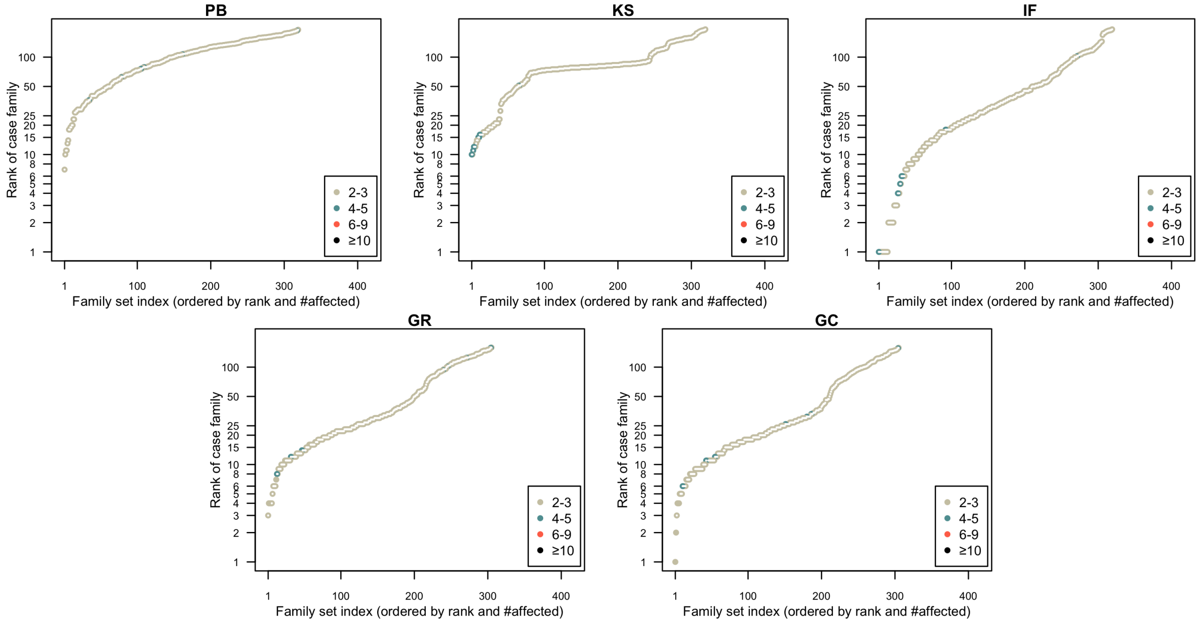

Supplementary Figure S10: Number of affected genereations $G = 2$, penetrance $Q = 30\%$, prevalence $R = 4\%$. All performance assessments are shown.

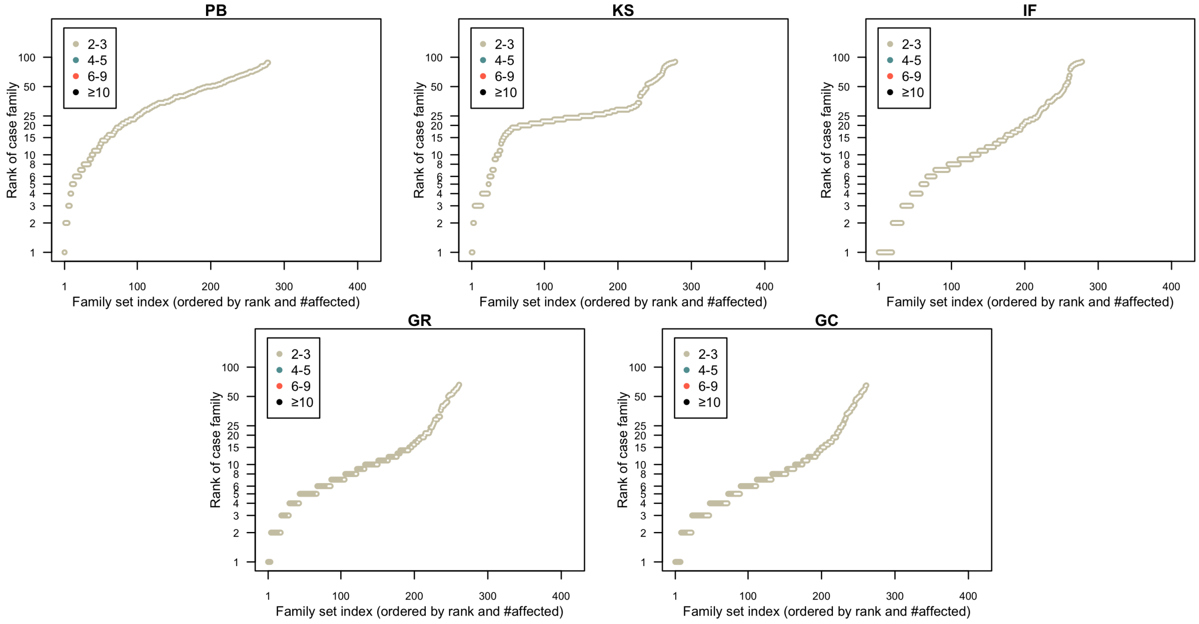

Supplementary Figure S11: Number of affected genereations $G = 2$, penetrance $Q = 30\%$, prevalence $R = 2\%$. All performance assessments are shown.

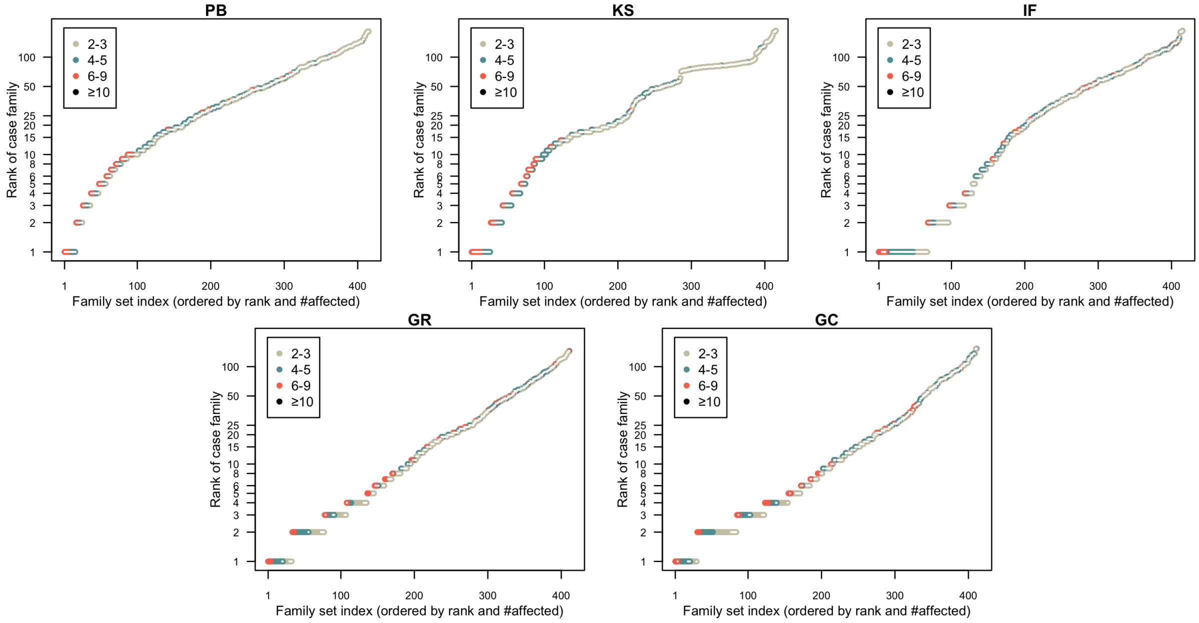

Supplementary Figure S12: Number of affected genereations $G = 2$, penetrance $Q = 60\%$, prevalence $R = 4\%$. All performance assessments are shown.

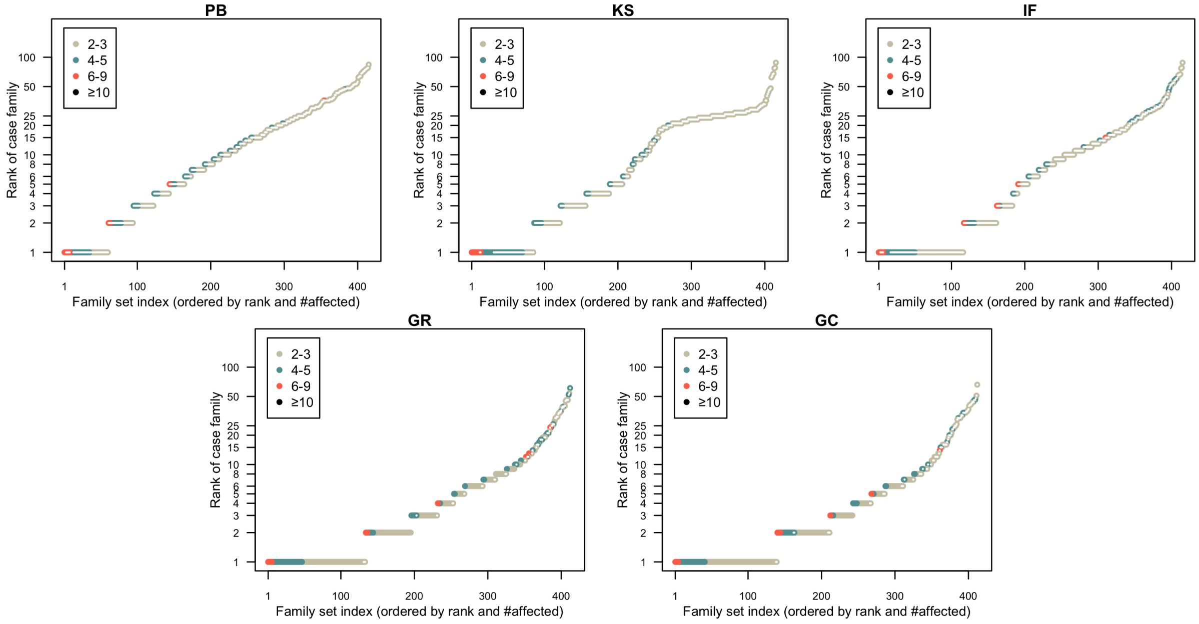

Supplementary Figure S13: Number of affected genereations $G = 2$, penetrance $Q = 60\%$, prevalence $R = 2\%$. All performance assessments are shown.

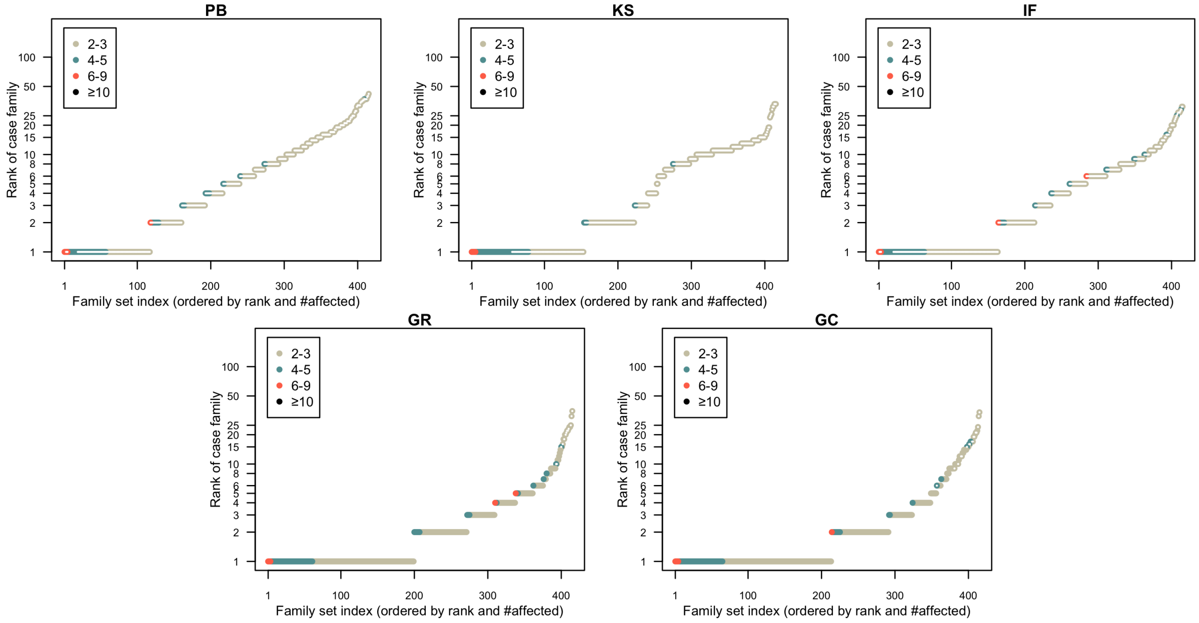

Supplementary Figure S14: Number of affected genereations $G = 2$, penetrance $Q = 60\%$, prevalence $R = 1.25\%$. All performance assessments are shown.

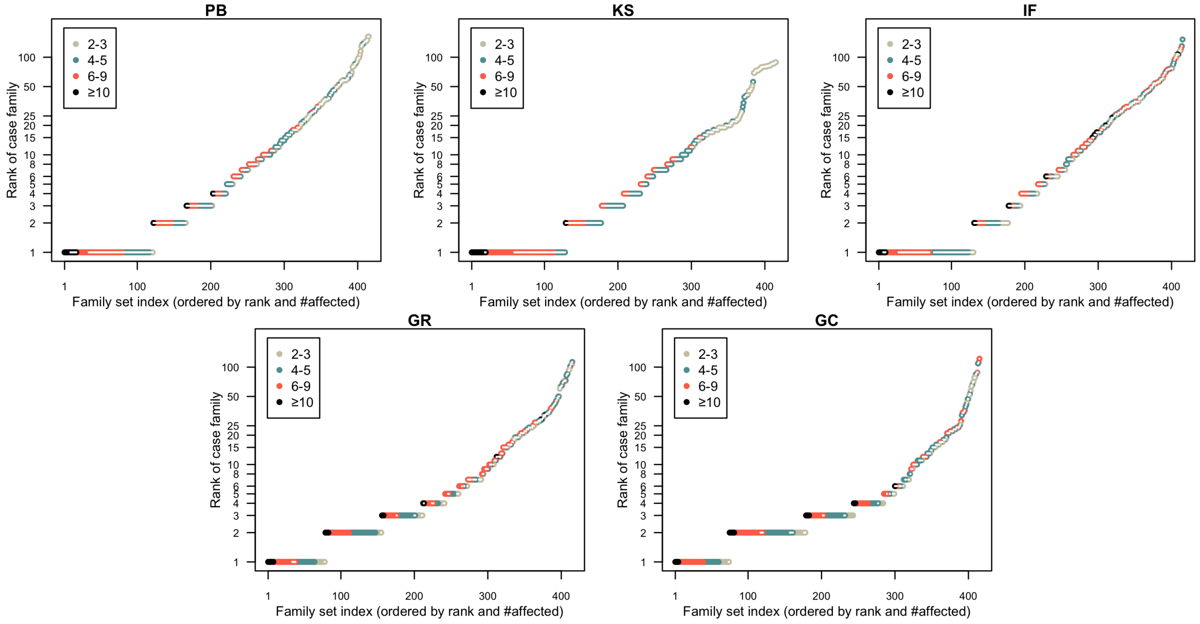

Supplementary Figure S15: Number of affected genereations $G = 2$, penetrance $Q = 100\%$, prevalence $R = 4\%$. All performance assessments are shown.

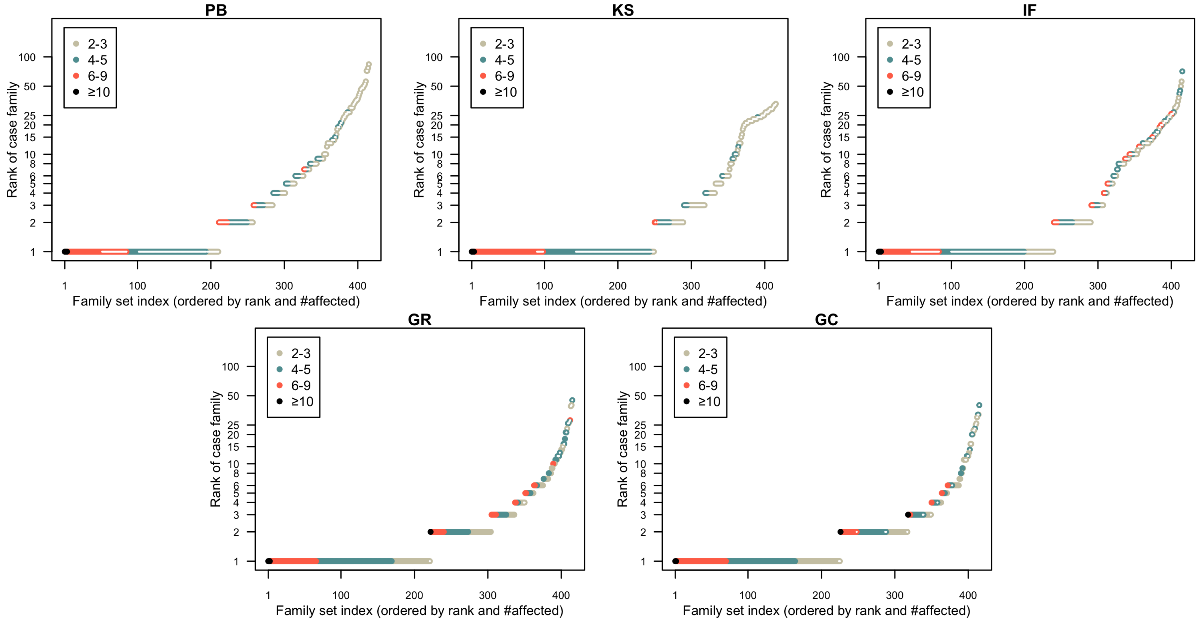

Supplementary Figure S16: Number of affected genereations $G = 2$, penetrance $Q = 100\%$, prevalence $R = 2\%$. All performance assessments are shown.

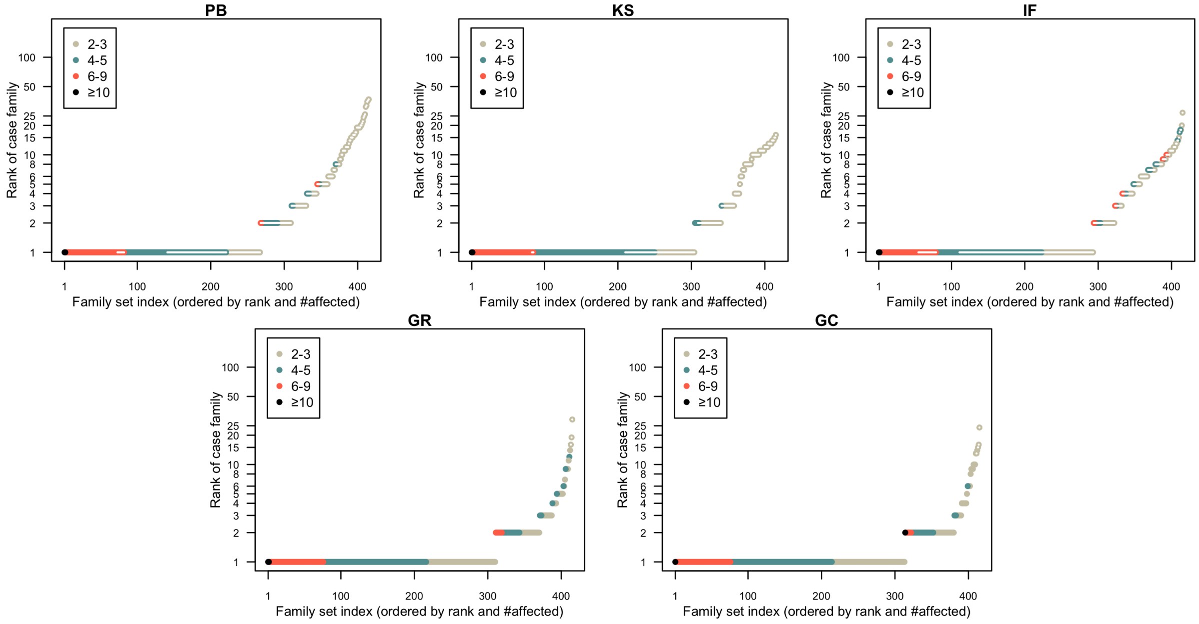

Supplementary Figure S17: Number of affected genereations $G = 2$, penetrance $Q = 100\%$, prevalence $R = 1.25\%$. All performance assessments are shown.

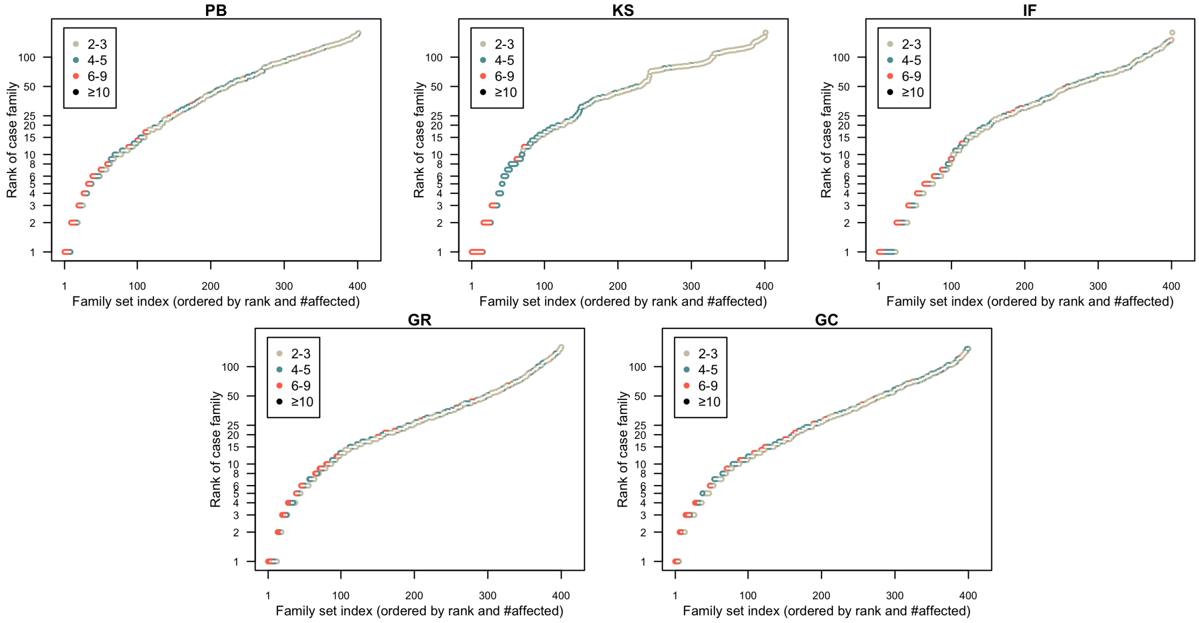

Supplementary Figure S18: Number of affected genereations $G = 3$, penetrance $Q = 30\%$, prevalence $R = 4\%$. All performance assessments are shown.

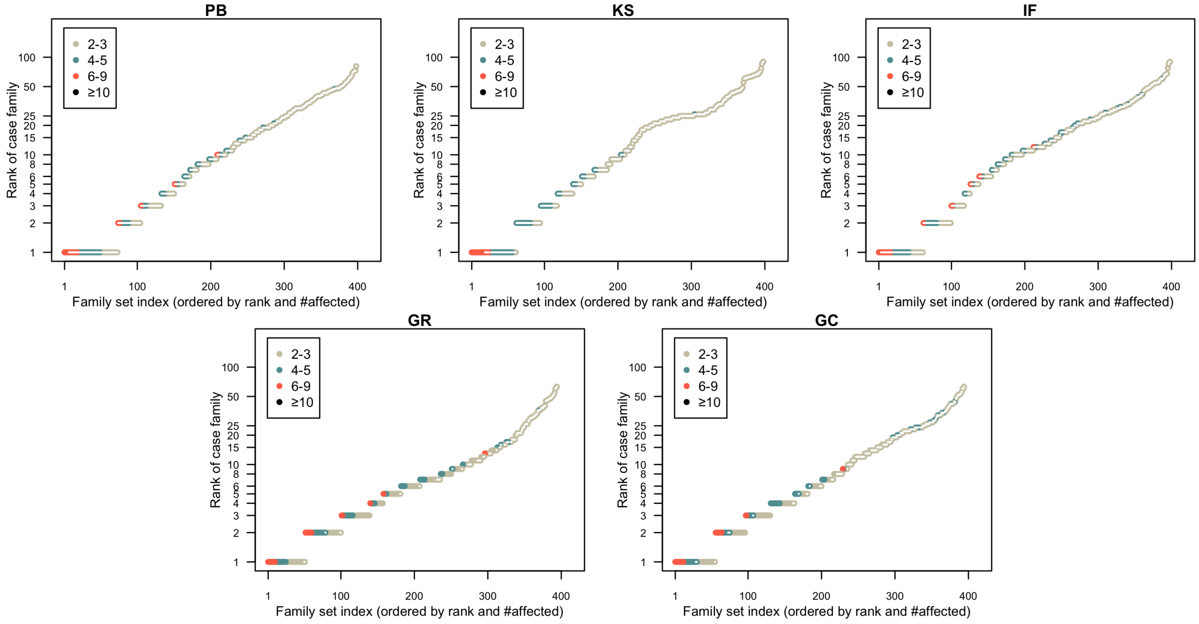

Supplementary Figure S19: Number of affected genereations $G = 3$, penetrance $Q = 30\%$, prevalence $R = 2\%$. All performance assessments are shown.

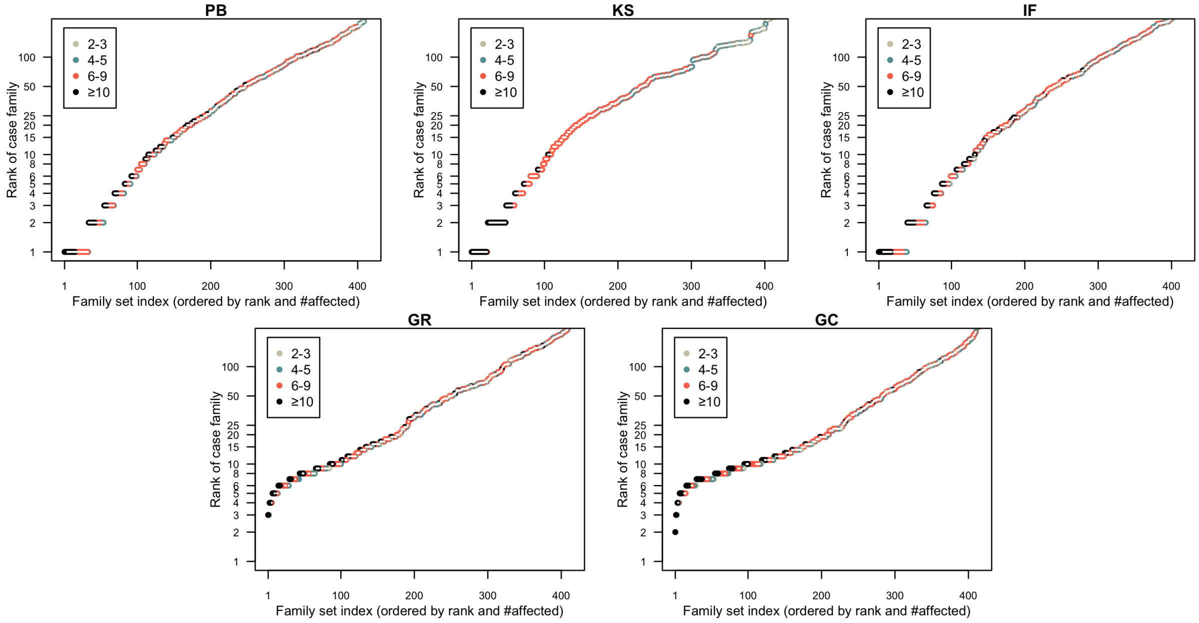

Supplementary Figure S20: Number of affected genereations $G = 3$, penetrance $Q = 60\%$, prevalence $R = 10\%$. All performance assessments are shown.

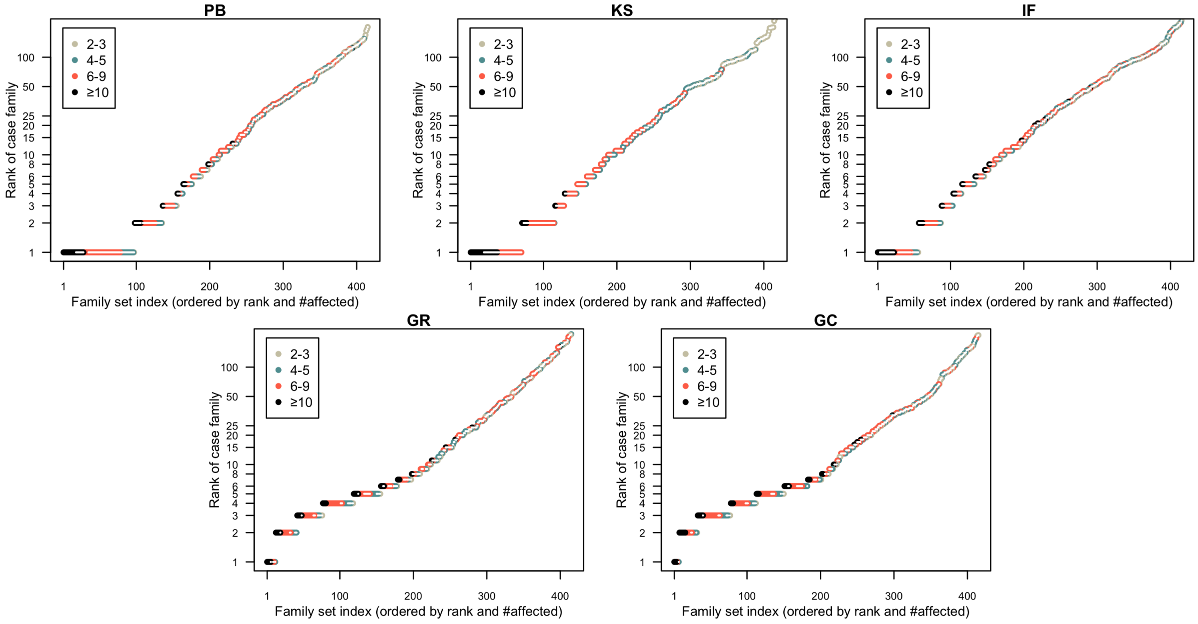

Supplementary Figure S21: Number of affected genereations $G = 3$, penetrance $Q = 60\%$, prevalence $R = 6.25\%$. All performance assessments are shown.

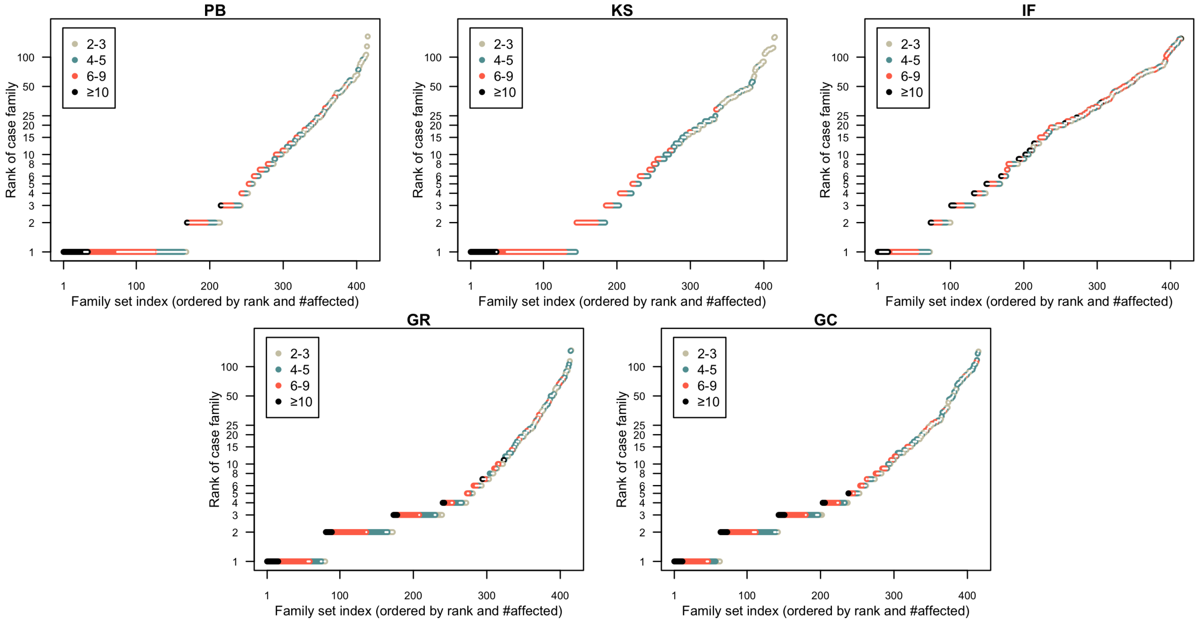

Supplementary Figure S22: Number of affected genereations $G = 3$, penetrance $Q = 60\%$, prevalence $R = 4\%$. All performance assessments are shown.

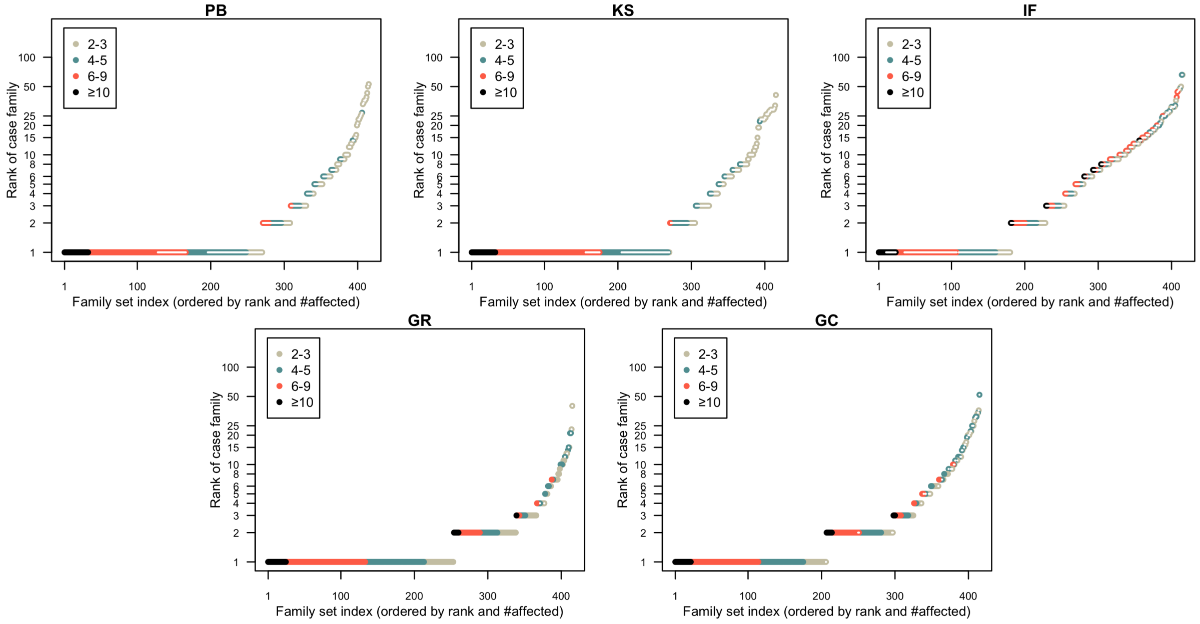

Supplementary Figure S23: Number of affected genereations $G = 3$, penetrance $Q = 60\%$, prevalence $R = 2\%$. All performance assessments are shown.

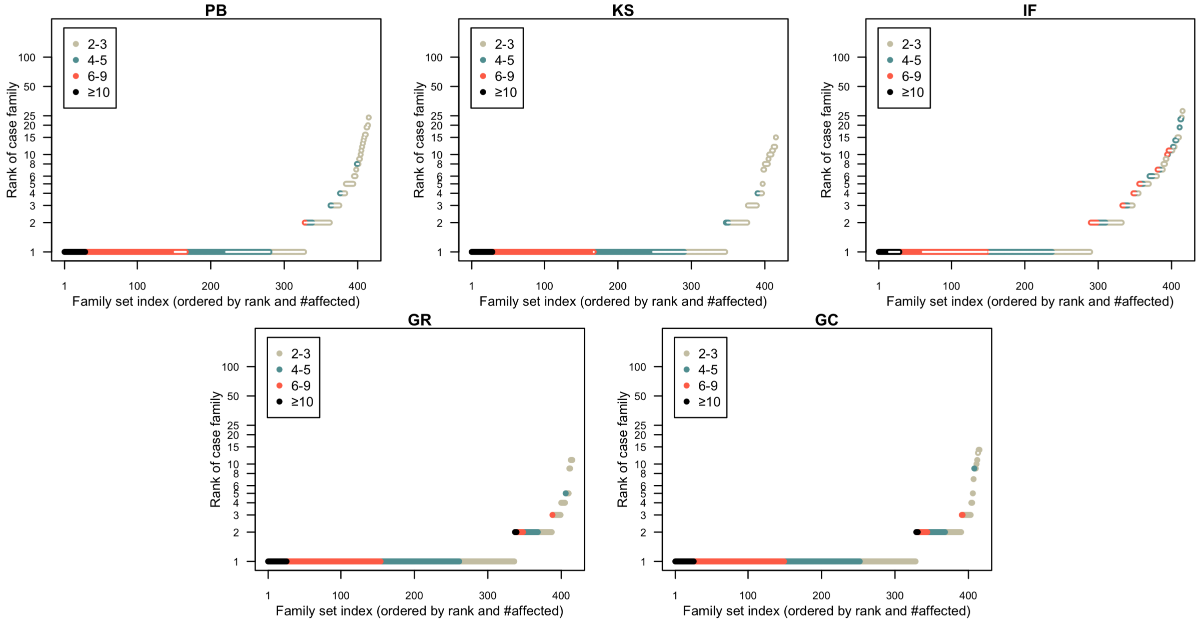

Supplementary Figure S24: Number of affected genereations $G = 3$, penetrance $Q = 60\%$, prevalence $R = 1.25\%$. All performance assessments are shown.

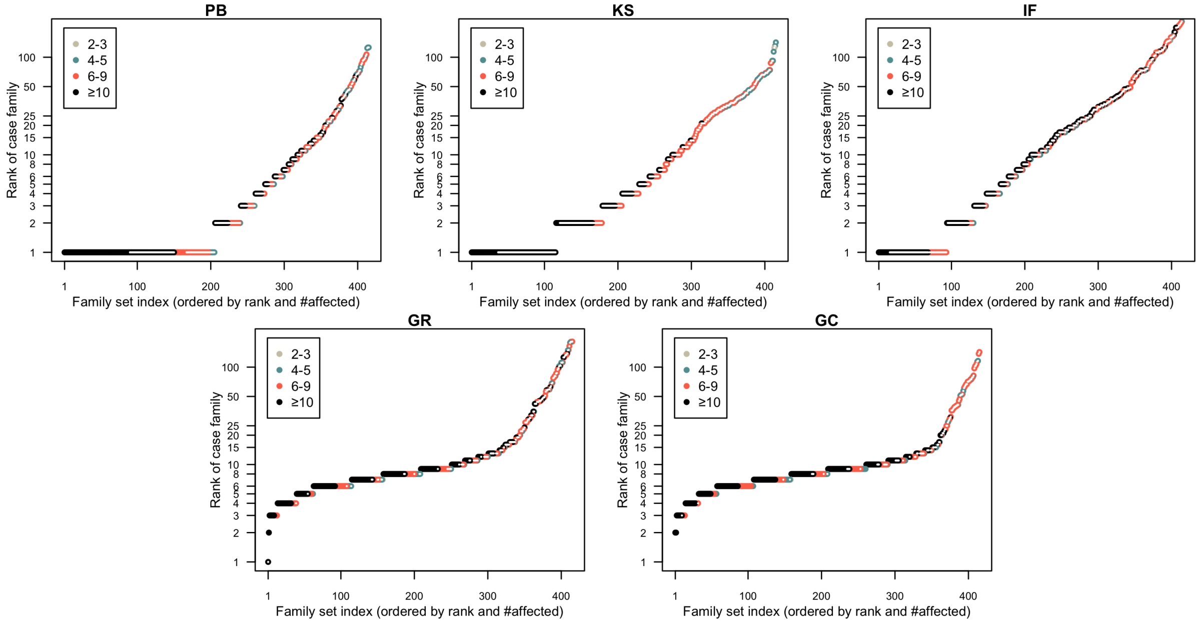

Supplementary Figure S25: Number of affected genereations $G = 3$, penetrance $Q = 100\%$, prevalence $R = 10\%$. All performance assessments are shown.

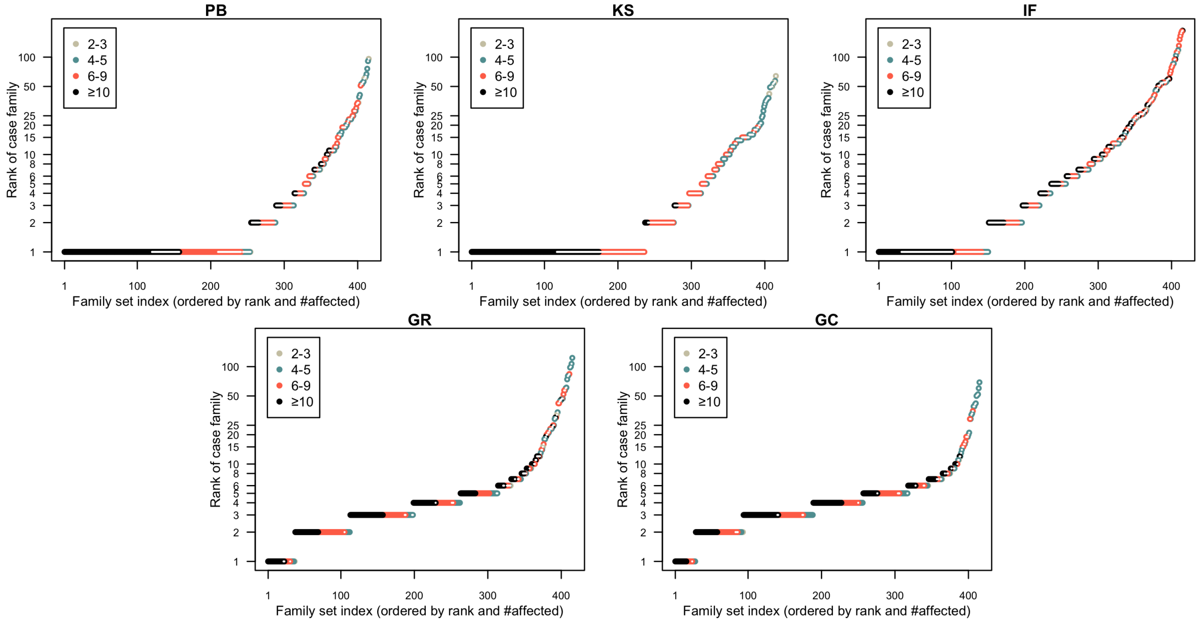

Supplementary Figure S26: Number of affected genereations $G = 3$, penetrance $Q = 100\%$, prevalence $R = 6.25\%$. All performance assessments are shown.

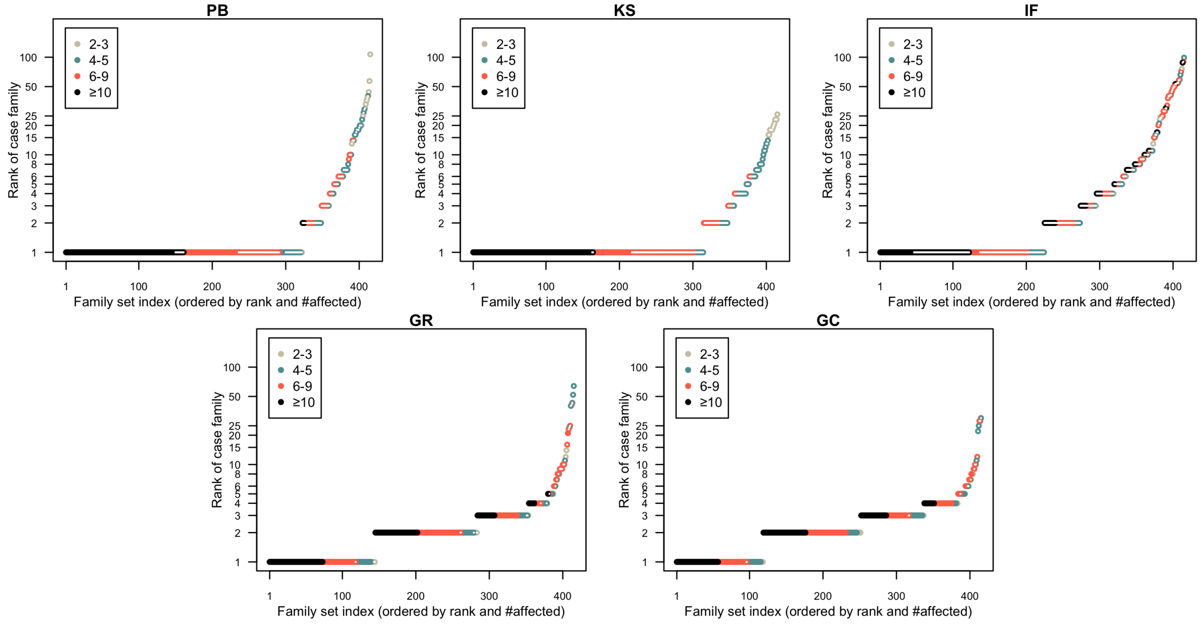

Supplementary Figure S27: Number of affected genereations $G = 3$, penetrance $Q = 100\%$, prevalence $R = 4\%$. All performance assessments are shown.

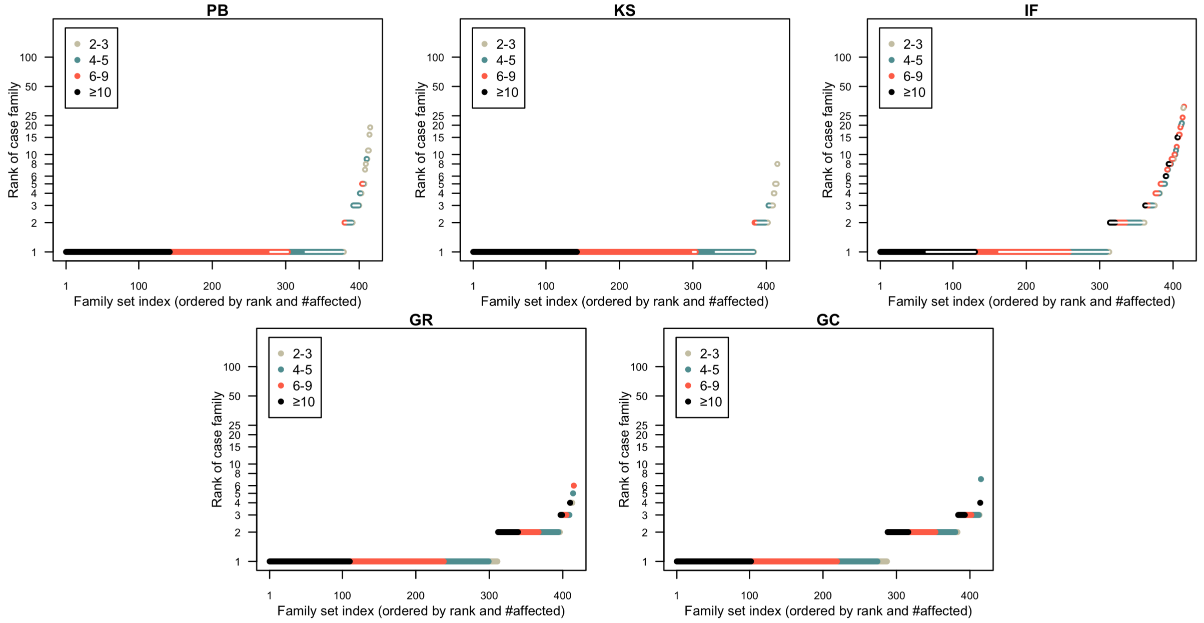

Supplementary Figure S28: Number of affected genereations $G = 3$, penetrance $Q = 100\%$, prevalence $R = 2\%$. All performance assessments are shown.

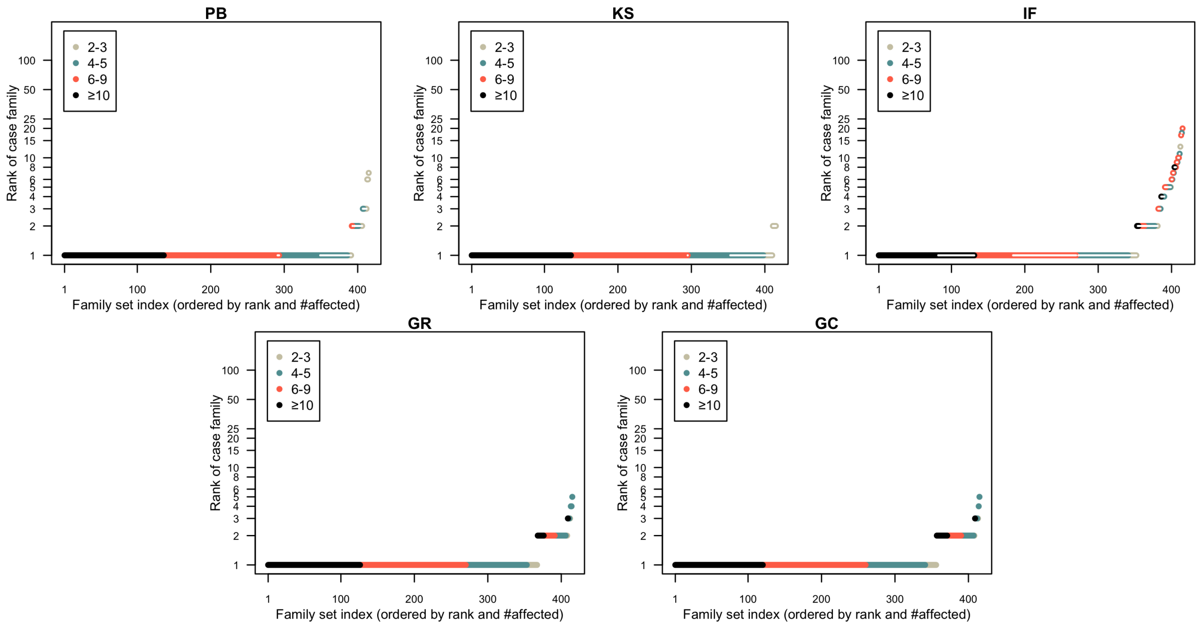

Supplementary Figure S29: Number of affected genereations $G = 3$, penetrance $Q = 100\%$, prevalence $R = 1.25\%$. All performance assessments are shown.

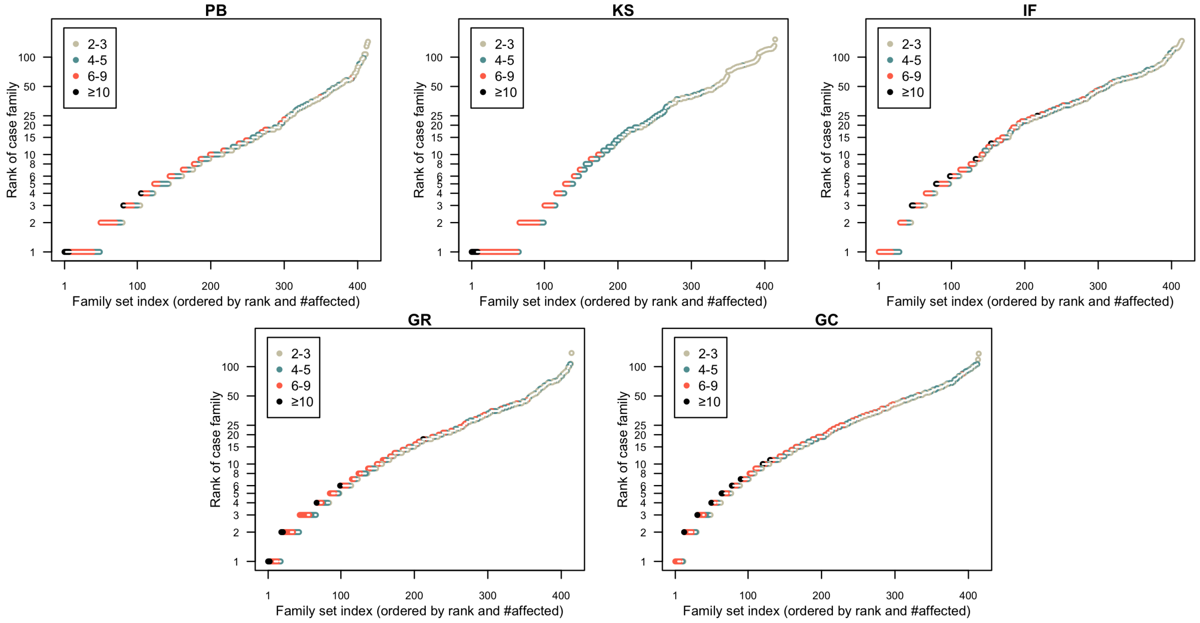

Supplementary Figure S30: Number of affected genereations $G =$ all, penetrance $Q = 30\%$, prevalence $R = 4\%$. All performance assessments are shown.

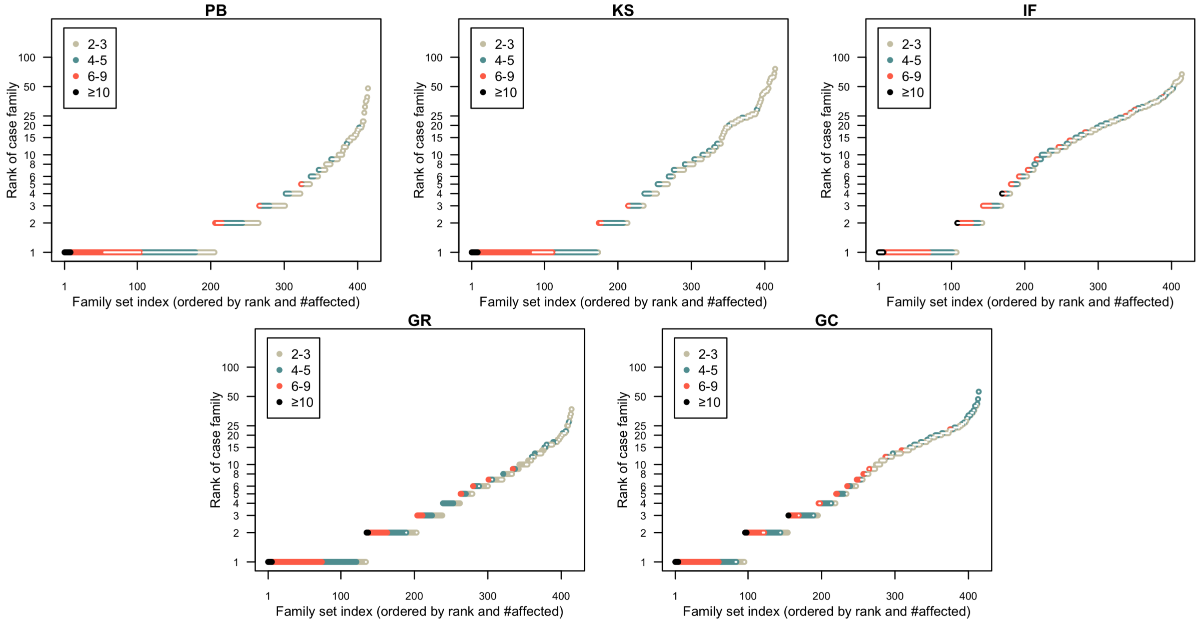

Supplementary Figure S31: Number of affected genereations G = all, penetrance $Q = 30\%$, prevalence $R = 2\%$. All performance assessments are shown.

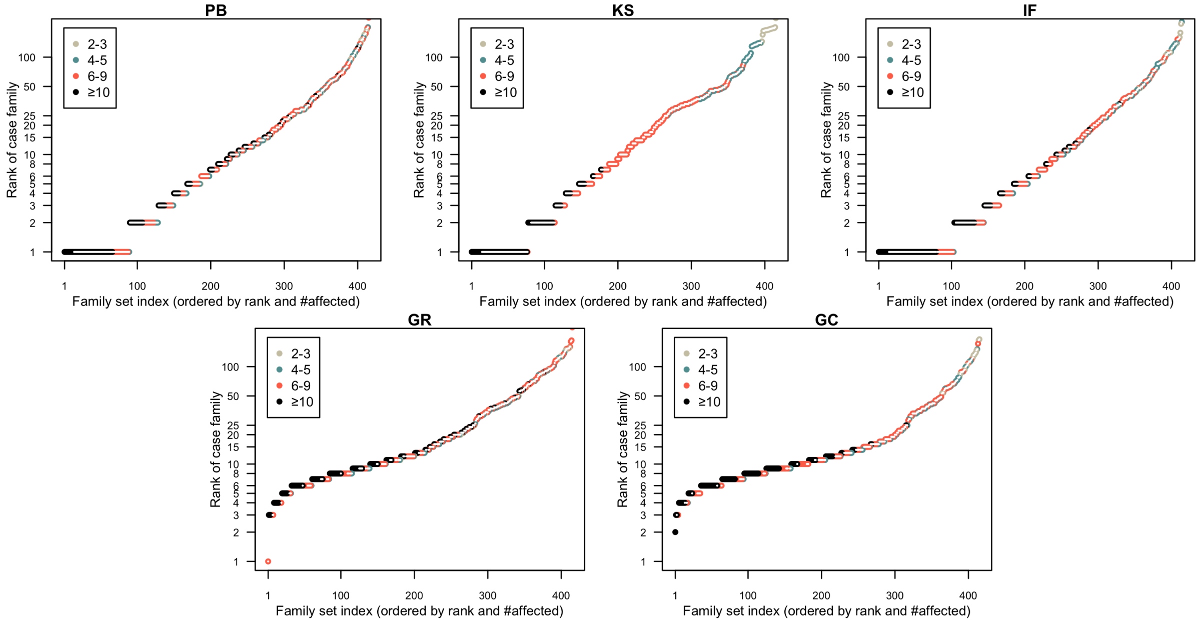

Supplementary Figure S32: Number of affected genereations G = all, penetrance $Q = 60\%$, prevalence $R = 10\%$. All performance assessments are shown.

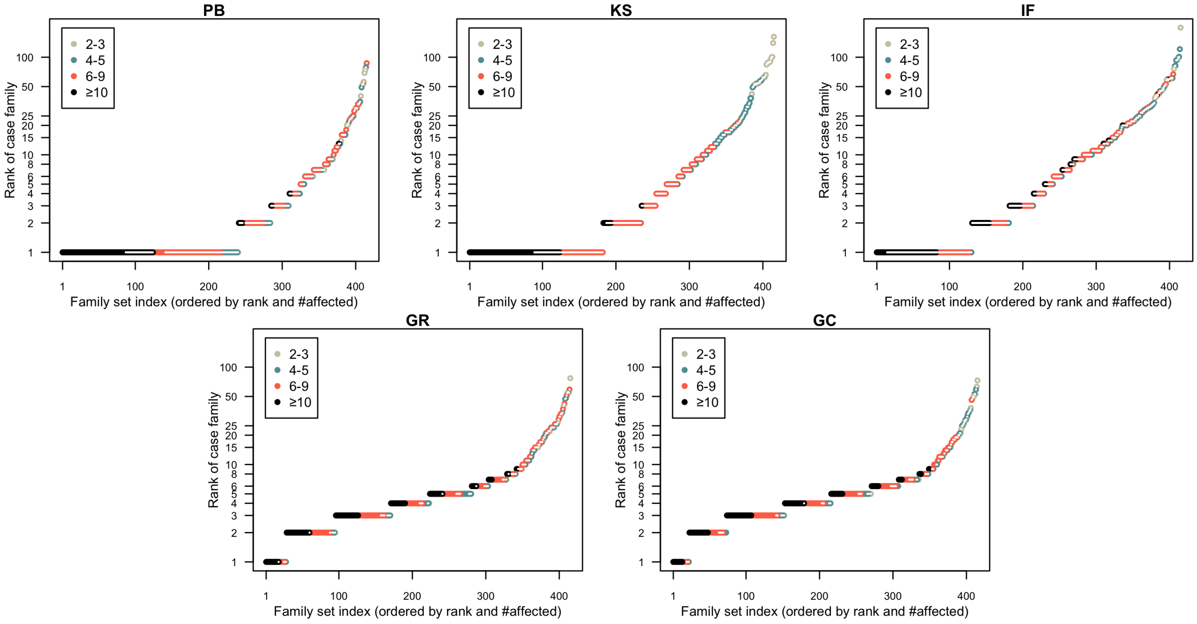

Supplementary Figure S33: Number of affected genereations G = all, penetrance $Q = 60\%$, prevalence $R = 6.25\%$. All performance assessments are shown.

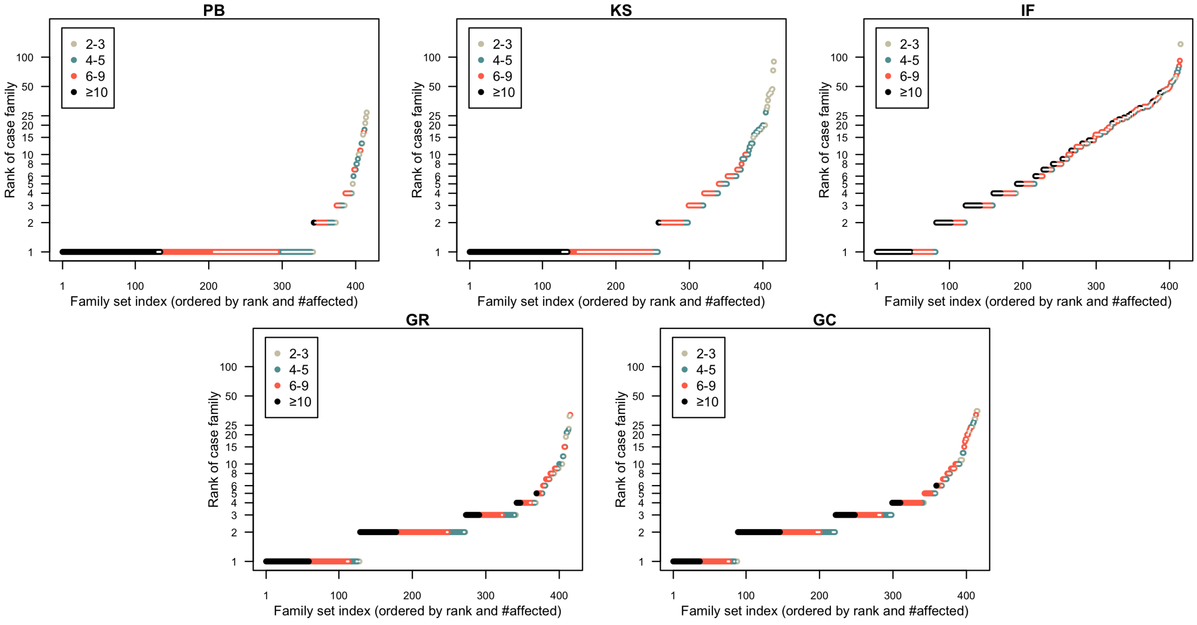

Supplementary Figure S34: Number of affected genereations G = all, penetrance $Q = 60\%$, prevalence $R = 4\%$. All performance assessments are shown.

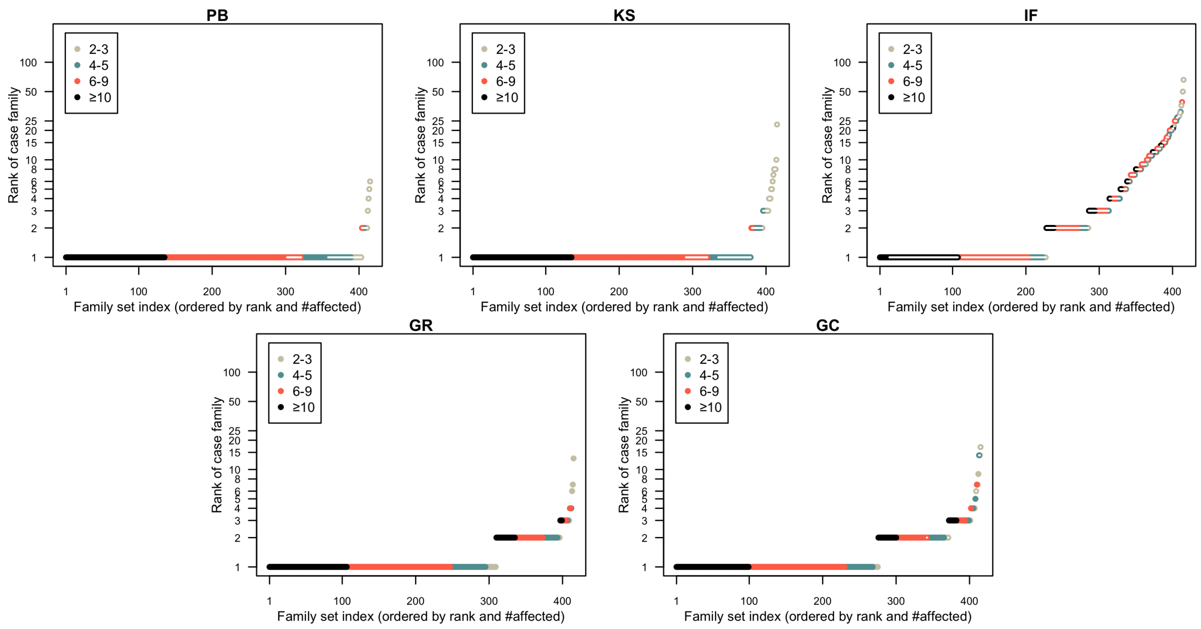

Supplementary Figure S35: Number of affected genereations G = all, penetrance $Q = 60\%$, prevalence $R = 2\%$. All performance assessments are shown.

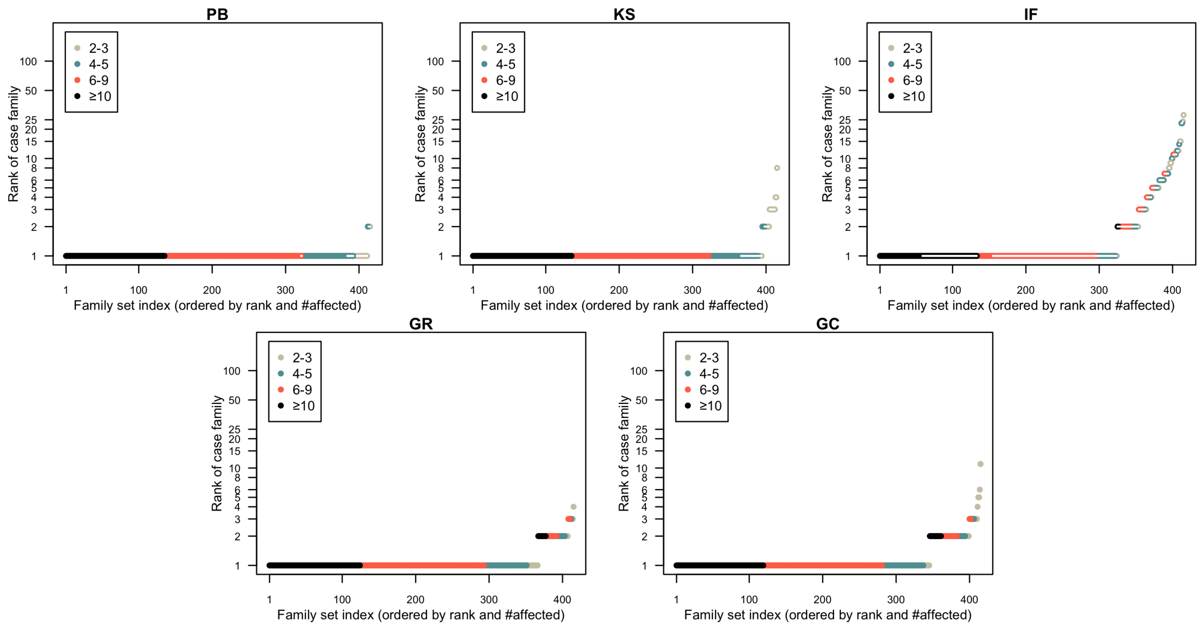

Supplementary Figure S36: Number of affected genereations G = all, penetrance $Q = 60\%$, prevalence $R = 1.25\%$. All performance assessments are shown.

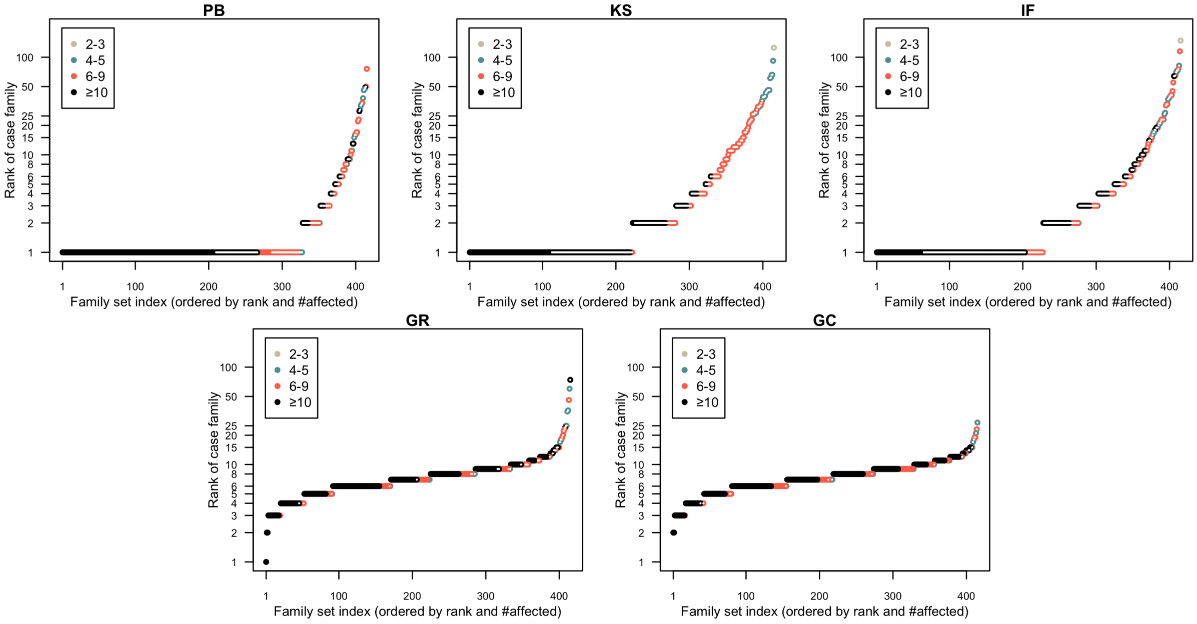

Supplementary Figure S37: Number of affected genereations G = all, penetrance $Q = 100\%$, prevalence $R = 10\%$. All performance assessments are shown.

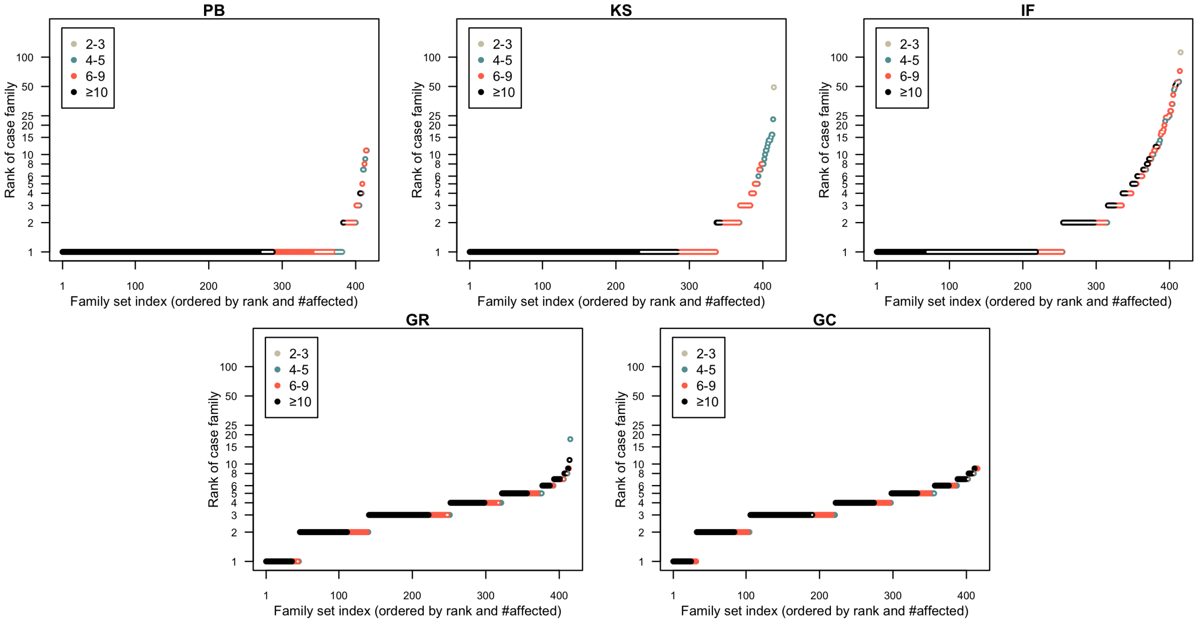

Supplementary Figure S38: Number of affected genereations G = all, penetrance $Q = 100\%$, prevalence $R = 6.25\%$. All performance assessments are shown.

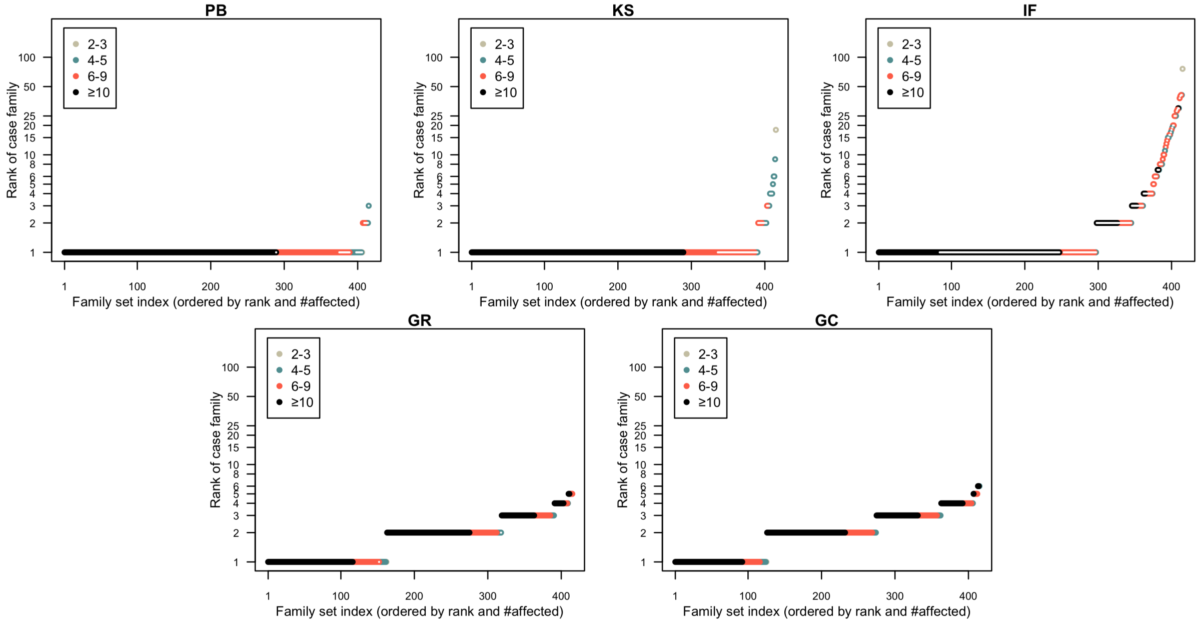

Supplementary Figure S39: Number of affected genereations G = all, penetrance $Q = 100\%$, prevalence $R = 4\%$. All performance assessments are shown.

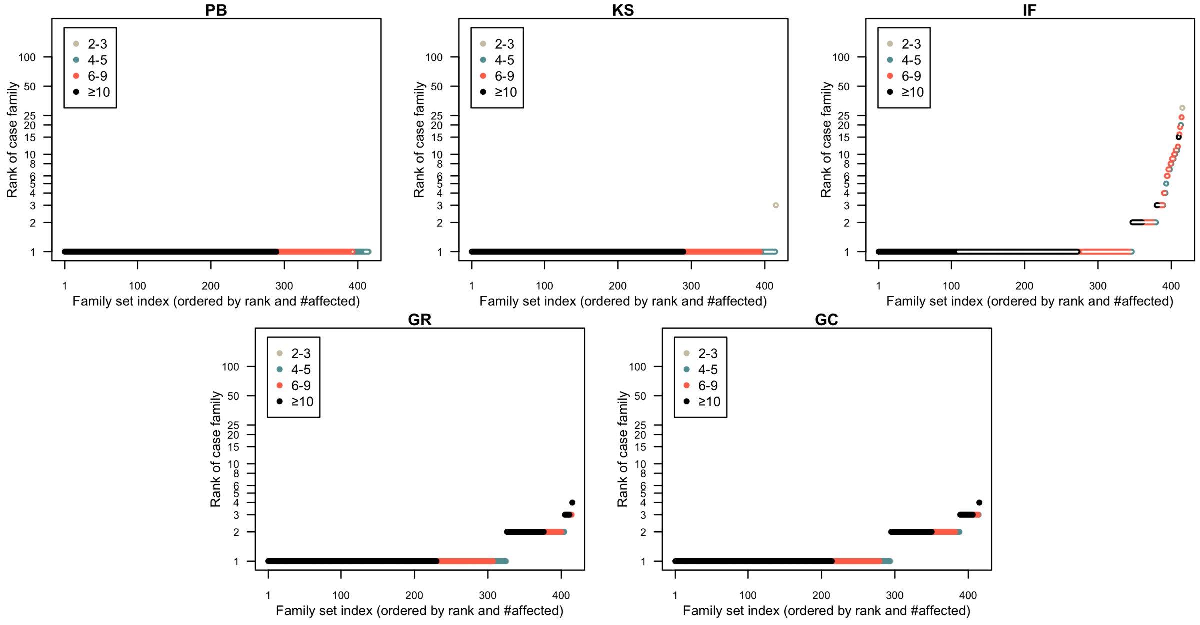

Supplementary Figure S40: Number of affected genereations G = all, penetrance $Q = 100\%$, prevalence $R = 2\%$. All performance assessments are shown.

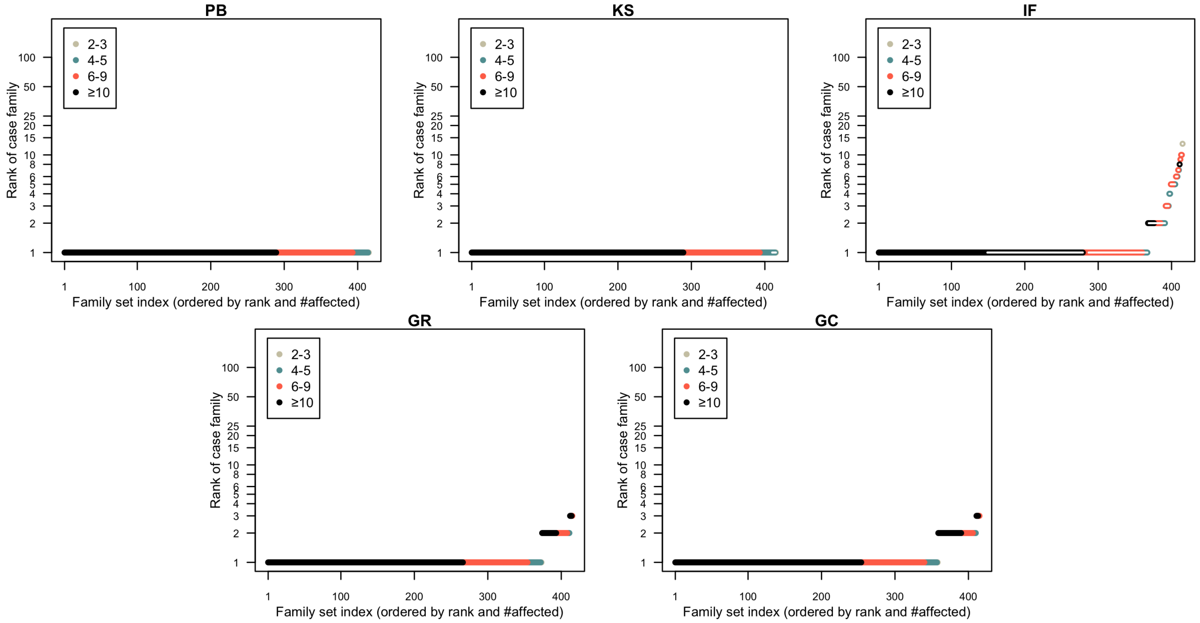

Supplementary Figure S41: Number of affected genereations G = all, penetrance $Q = 100\%$, prevalence $R = 1.25\%$. All performance assessments are shown.

3 Test overlap of top ranking case families

We have investigated how many top-ranking cases overlap between aggregation tests. For fixed parameters G, Q, and R, we take the cases that ranked first, independent on significance. For our five tests PB, KS, IF, GR, and GC this results in five sets, of those we are interested in their mutual overlaps. Compared to Venn diagrams, UpSet plots visualize overlaps between sets in a scalable way and are much easier to read and comprehend. The plots were generated using UpSetR [1], see Figure 2 for a detailed description of the plot.

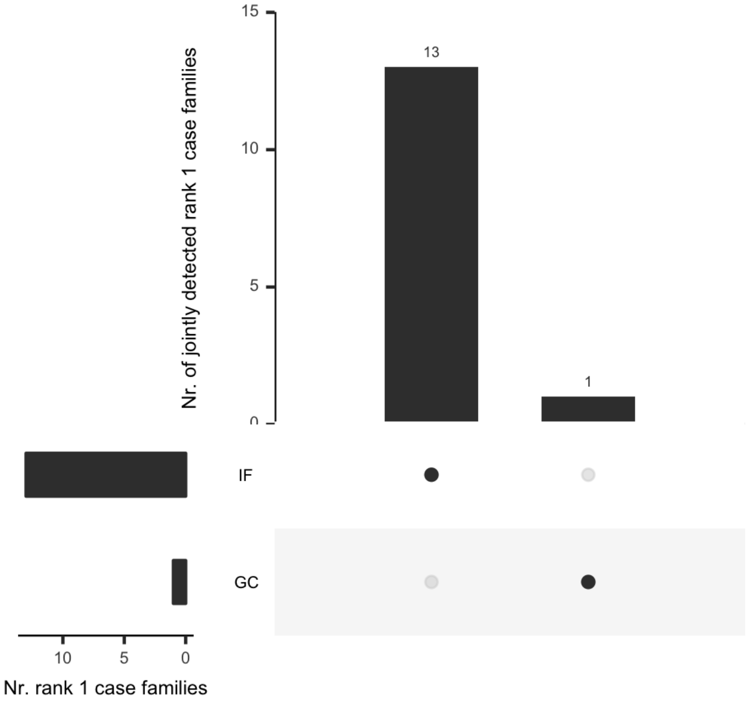

Supplementary Figure S42: Number of affected generations $G = 2$, penetrance $Q = 30\%$, prevalence $R = 4\%$. (See Figure 2 for a detailed description of the plot.)

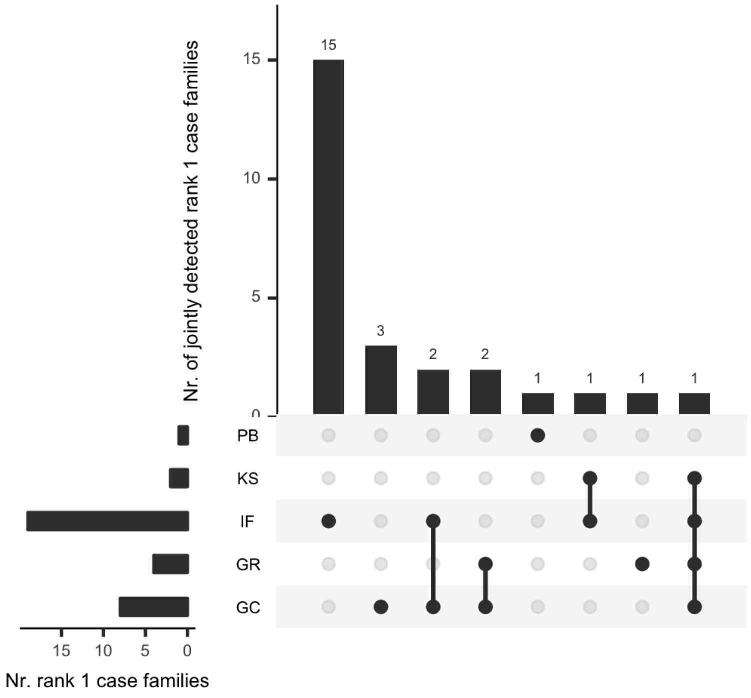

Supplementary Figure S43: Number of affected generations $G = 2$, penetrance $Q = 30\%$, prevalence $R = 2\%$. (See Figure 2 for a detailed description of the plot.)

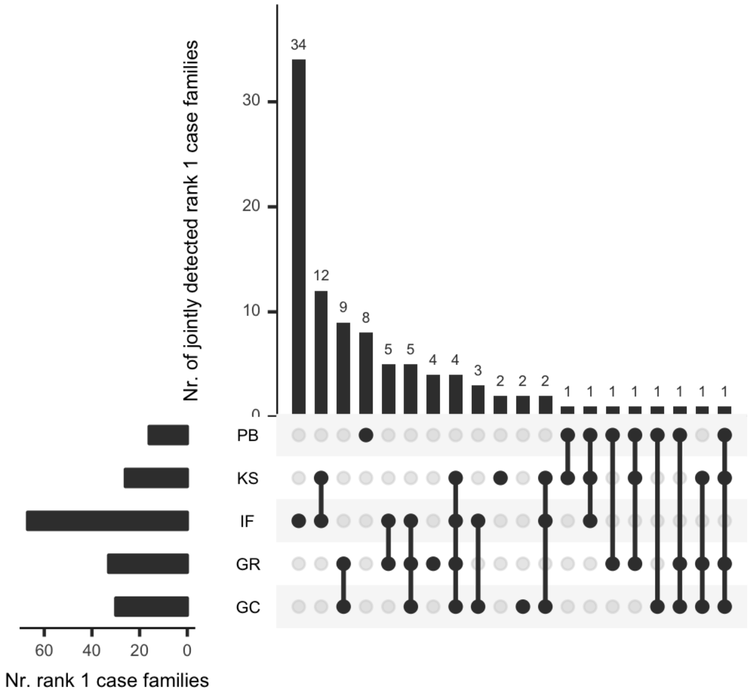

Supplementary Figure S44: Number of affected generations $G = 2$, penetrance $Q = 60\%$, prevalence $R = 4\%$. (See Figure 2 for a detailed description of the plot.)

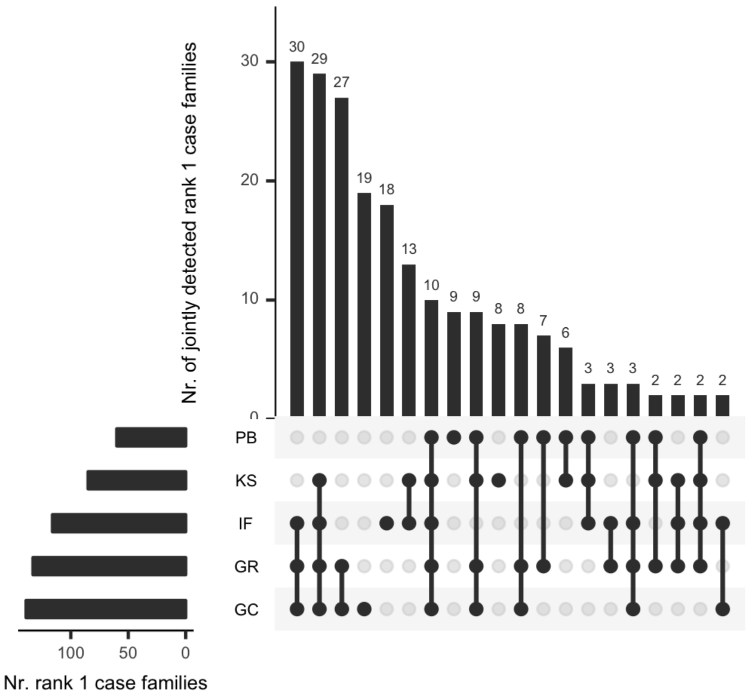

Supplementary Figure S45: Number of affected generations $G = 2$, penetrance $Q = 60\%$, prevalence $R = 2\%$. (See Figure 2 for a detailed description of the plot.)

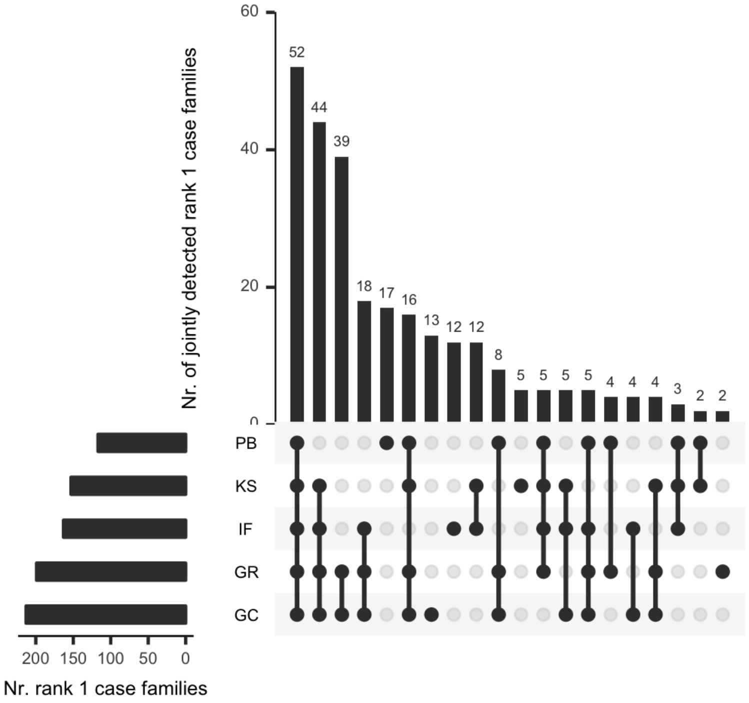

Supplementary Figure S46: Number of affected generations $G = 2$, penetrance $Q = 60\%$, prevalence $R = 1.25\%$. (See Figure 2 for a detailed description of the plot.)

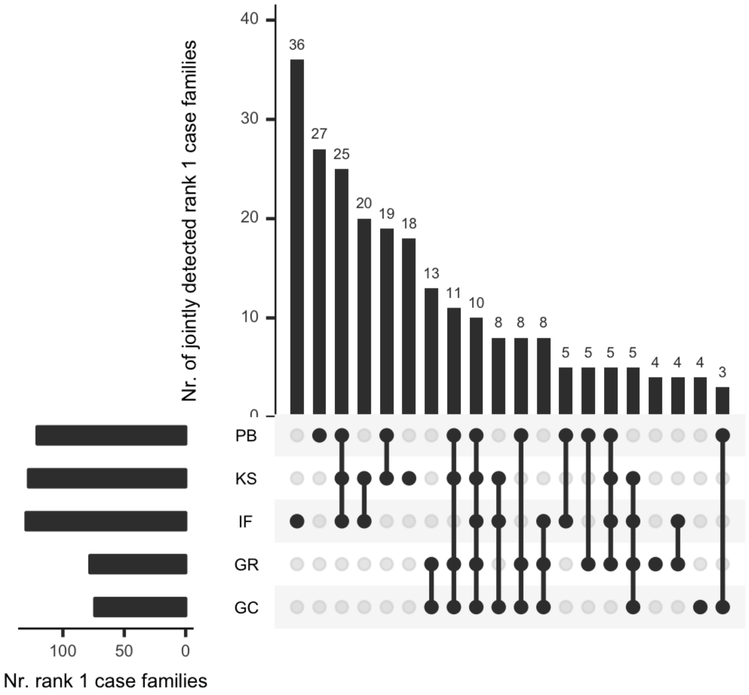

Supplementary Figure S47: Number of affected generations $G = 2$, penetrance $Q = 100\%$, prevalence $R = 4\%$. (See Figure 2 for a detailed description of the plot.)

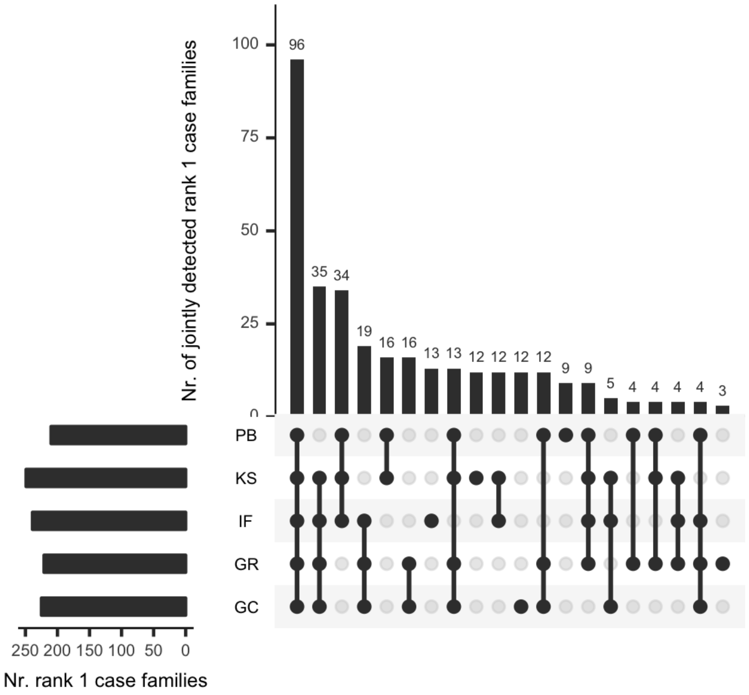

Supplementary Figure S48: Number of affected generations $G = 2$, penetrance $Q = 100\%$, prevalence $R = 2\%$. (See Figure 2 for a detailed description of the plot.)

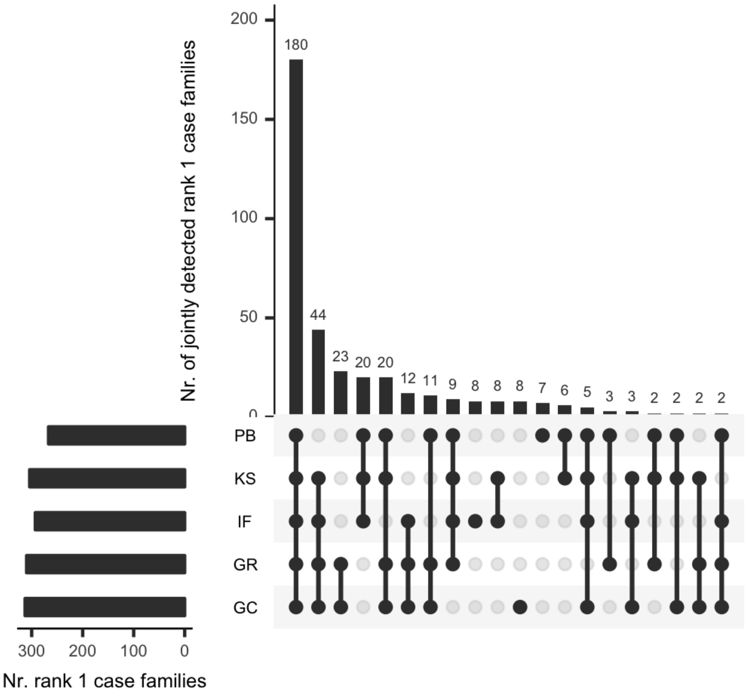

Supplementary Figure S49: Number of affected generations $G = 2$, penetrance $Q = 100\%$, prevalence $R = 1.25\%$. (See Figure 2 for a detailed description of the plot.)

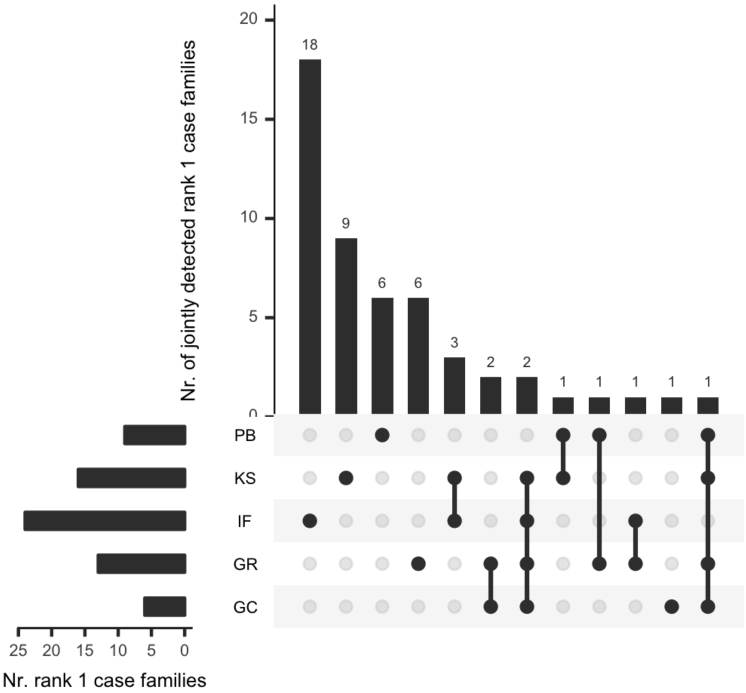

Supplementary Figure S50: Number of affected generations $G = 3$, penetrance $Q = 30\%$, prevalence $R = 4\%$. (See Figure 2 for a detailed description of the plot.)

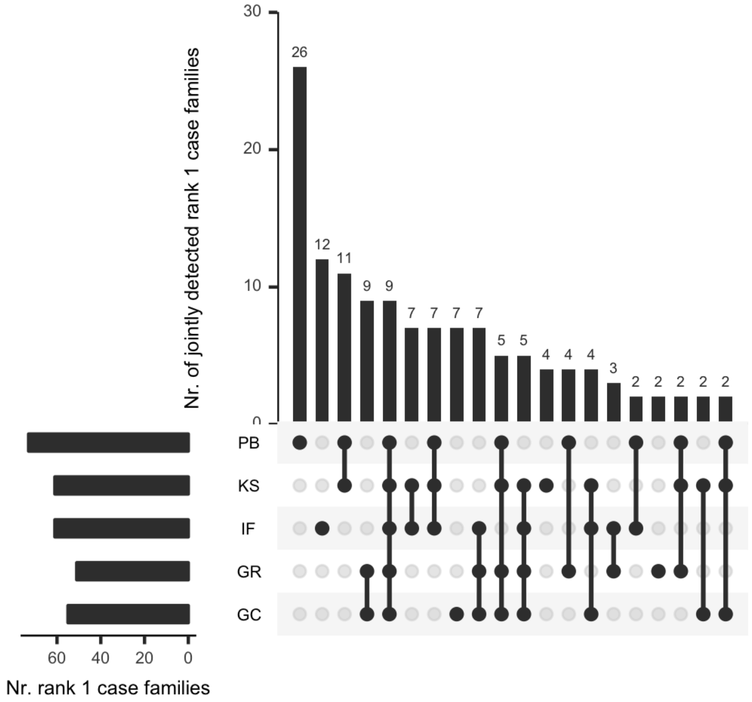

Supplementary Figure S51: Number of affected generations $G = 3$, penetrance $Q = 30\%$, prevalence $R = 2\%$. (See Figure 2 for a detailed description of the plot.)

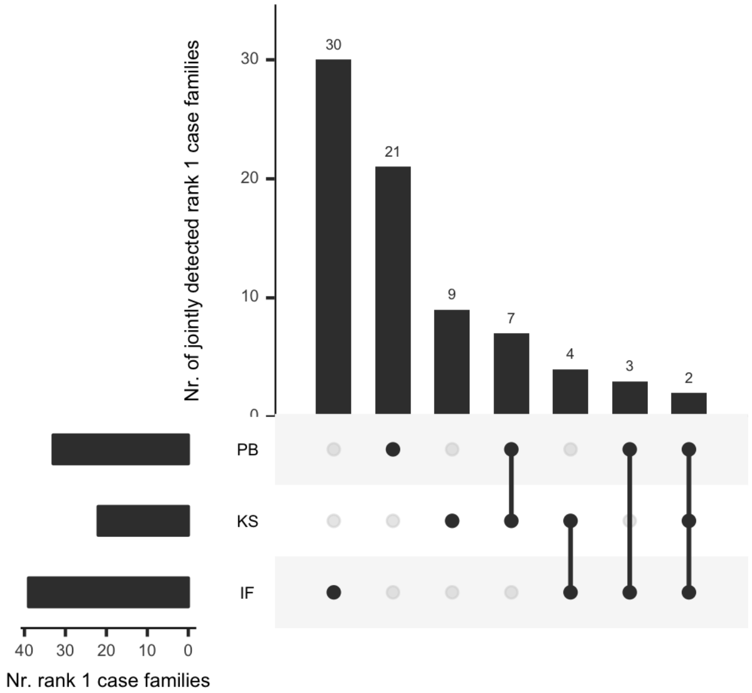

Supplementary Figure S52: Number of affected generations $G = 3$, penetrance $Q = 60\%$, prevalence $R = 10\%$. (See Figure 2 for a detailed description of the plot.)

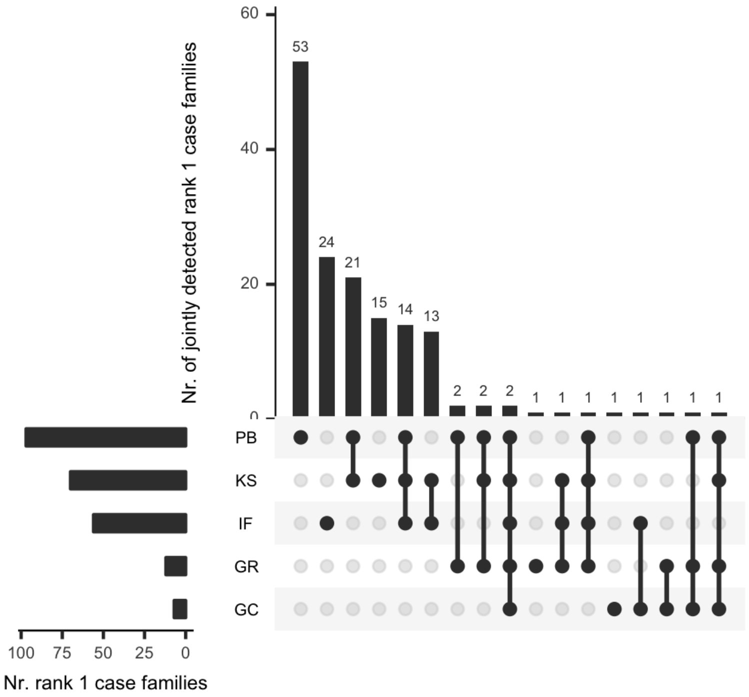

Supplementary Figure S53: Number of affected generations $G = 3$, penetrance $Q = 60\%$, prevalence $R = 6.25\%$. (See Figure 2 for a detailed description of the plot.)

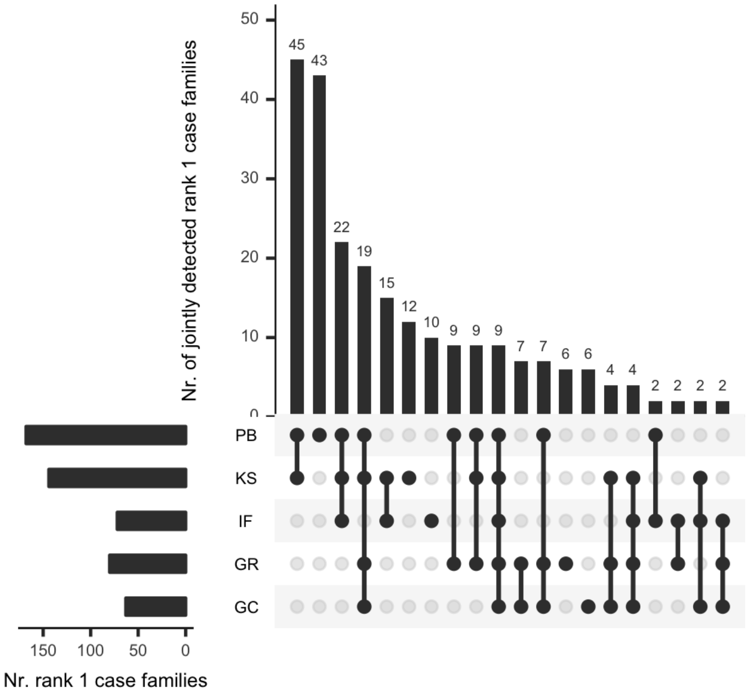

Supplementary Figure S54: Number of affected generations $G = 3$, penetrance $Q = 60\%$, prevalence $R = 4\%$. (See Figure 2 for a detailed description of the plot.)

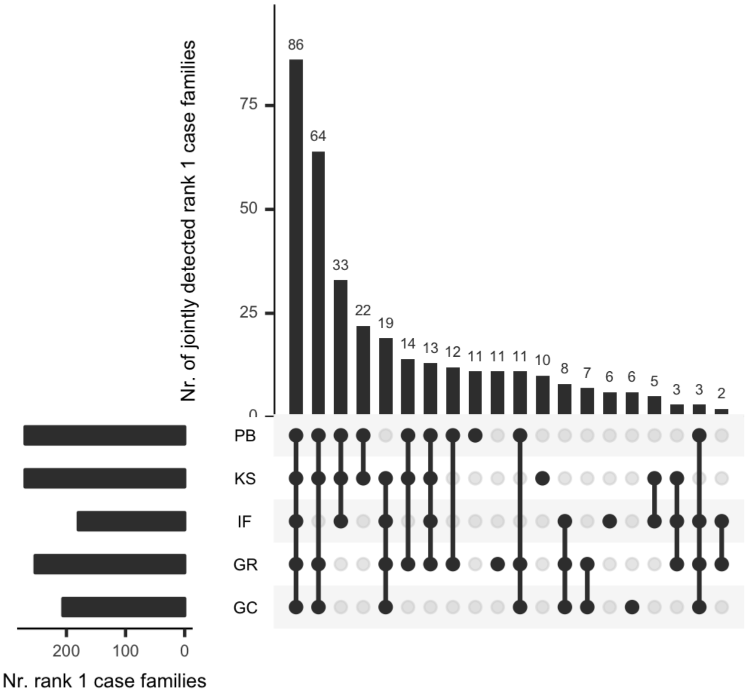

Supplementary Figure S55: Number of affected generations $G = 3$, penetrance $Q = 60\%$, prevalence $R = 2\%$. (See Figure 2 for a detailed description of the plot.)

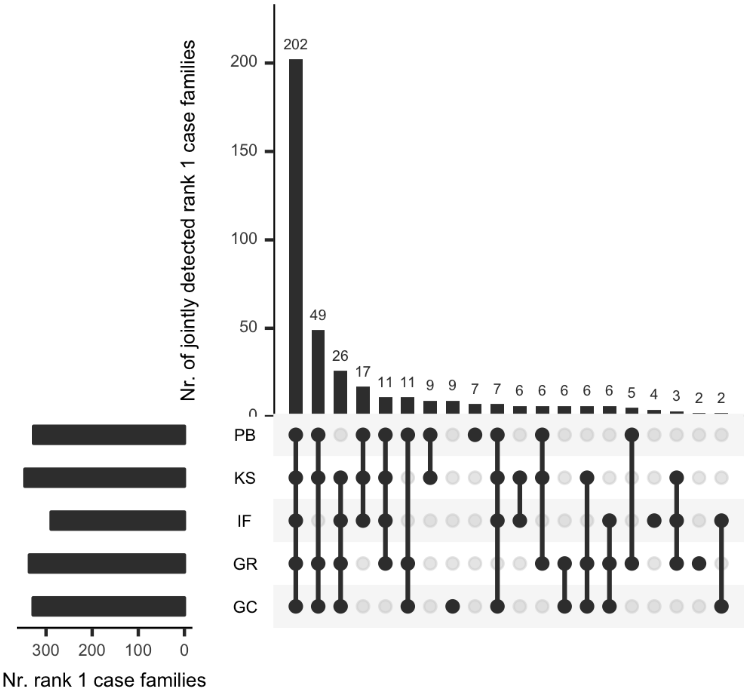

Supplementary Figure S56: Number of affected generations $G = 3$, penetrance $Q = 60\%$, prevalence $R = 1.25\%$. (See Figure 2 for a detailed description of the plot.)

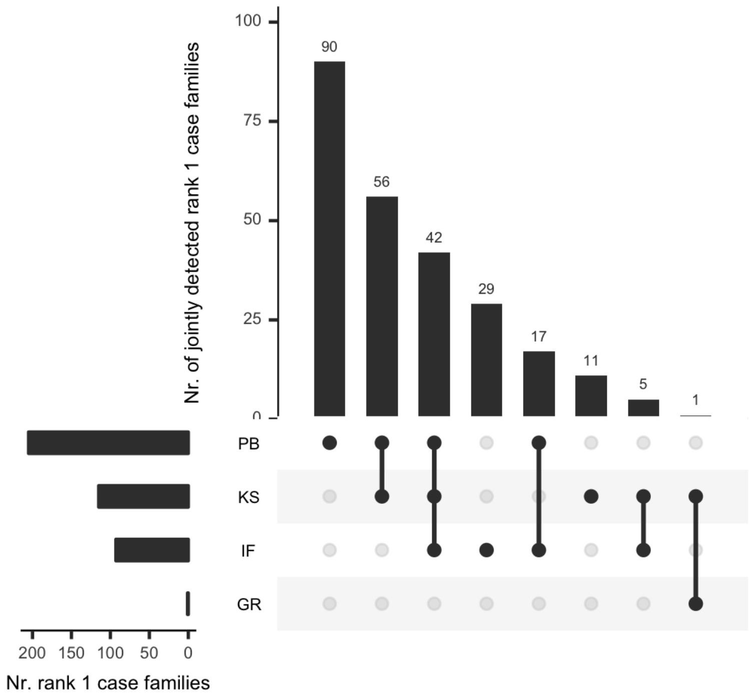

Supplementary Figure S57: Number of affected generations $G = 3$, penetrance $Q = 100\%$, prevalence $R = 10\%$. (See Figure 2 for a detailed description of the plot.)

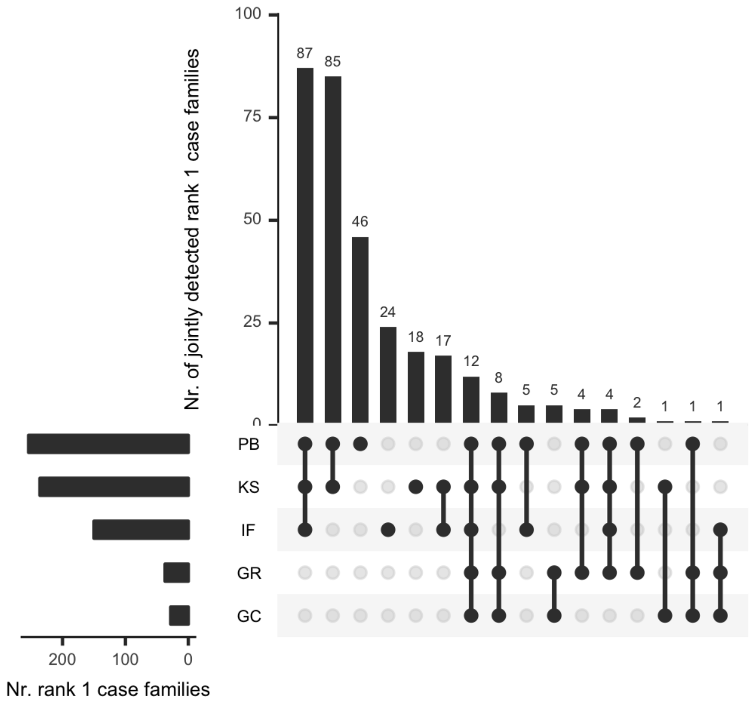

Supplementary Figure S58: Number of affected generations $G = 3$, penetrance $Q = 100\%$, prevalence $R = 6.25\%$. (See Figure 2 for a detailed description of the plot.)

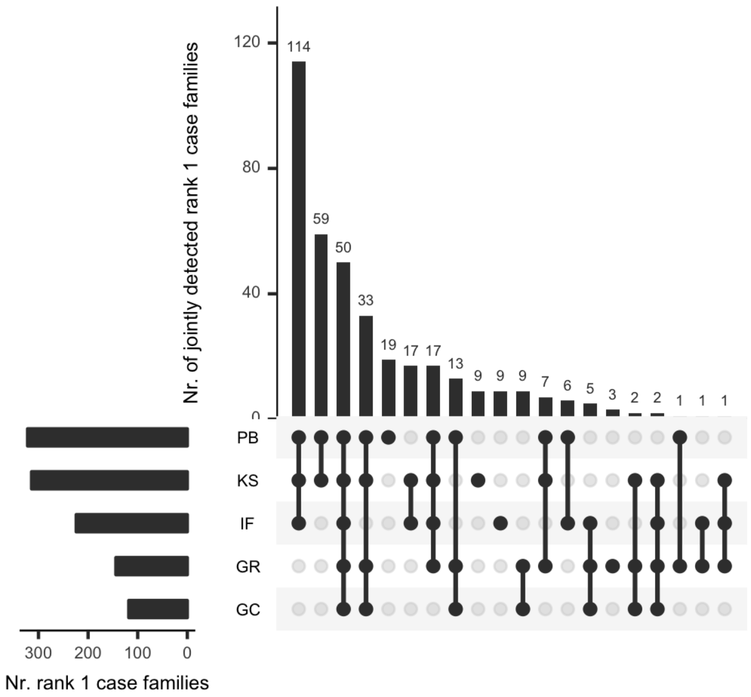

Supplementary Figure S59: Number of affected generations $G = 3$, penetrance $Q = 100\%$, prevalence $R = 4\%$. (See Figure 2 for a detailed description of the plot.)

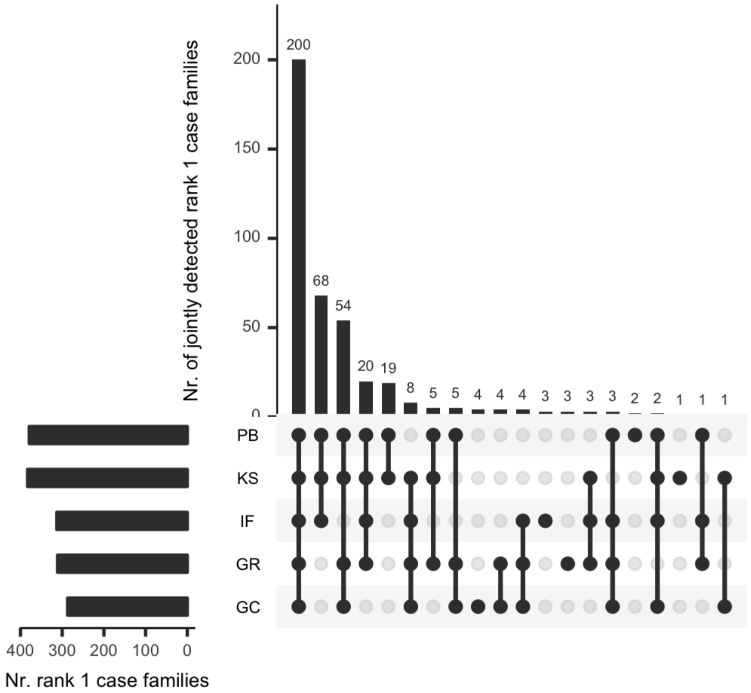

Supplementary Figure S60: Number of affected generations $G = 3$, penetrance $Q = 100\%$, prevalence $R = 2\%$. (See Figure 2 for a detailed description of the plot.)

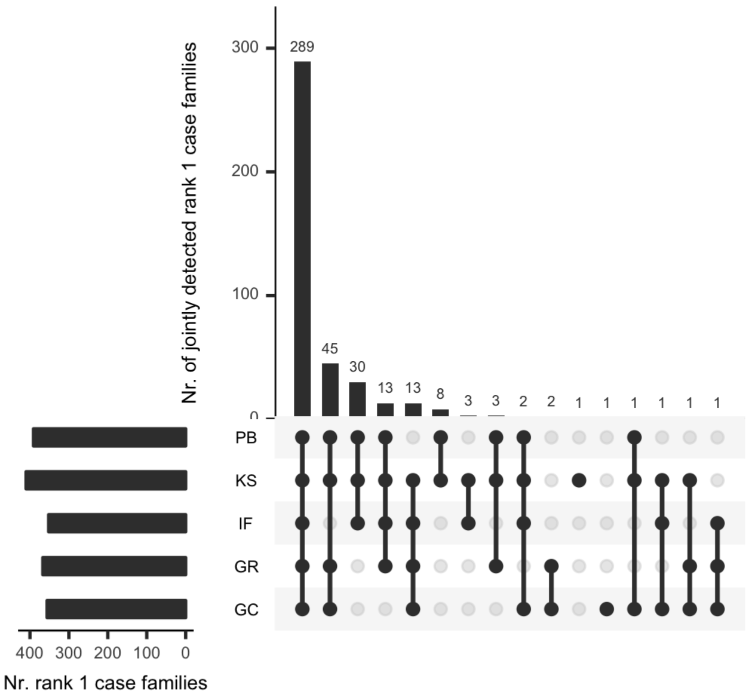

Supplementary Figure S61: Number of affected generations $G = 3$, penetrance $Q = 100\%$, prevalence $R = 1.25\%$. (See Figure 2 for a detailed description of the plot.)

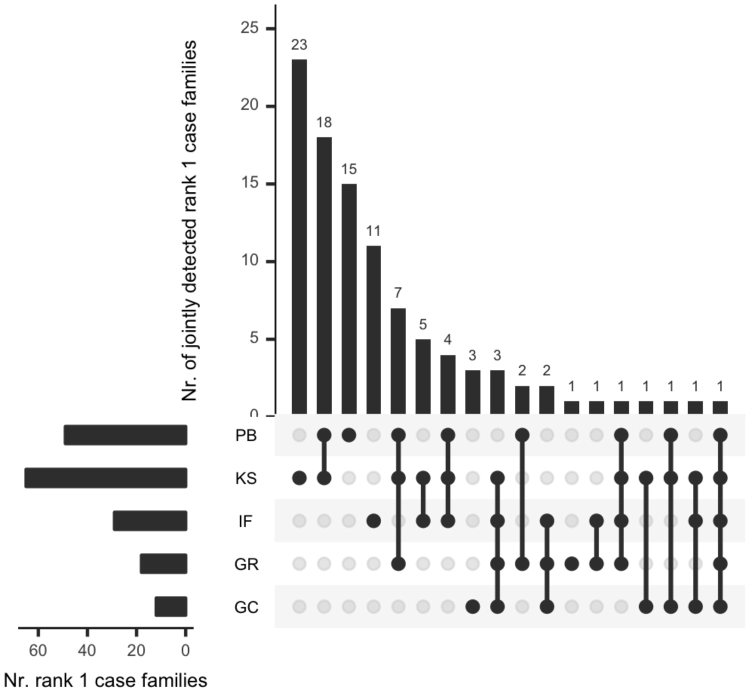

Supplementary Figure S62: Number of affected generations $G =$ all, penetrance $Q = 30\%$, prevalence $R = 4\%$. (See Figure 2 for a detailed description of the plot.)

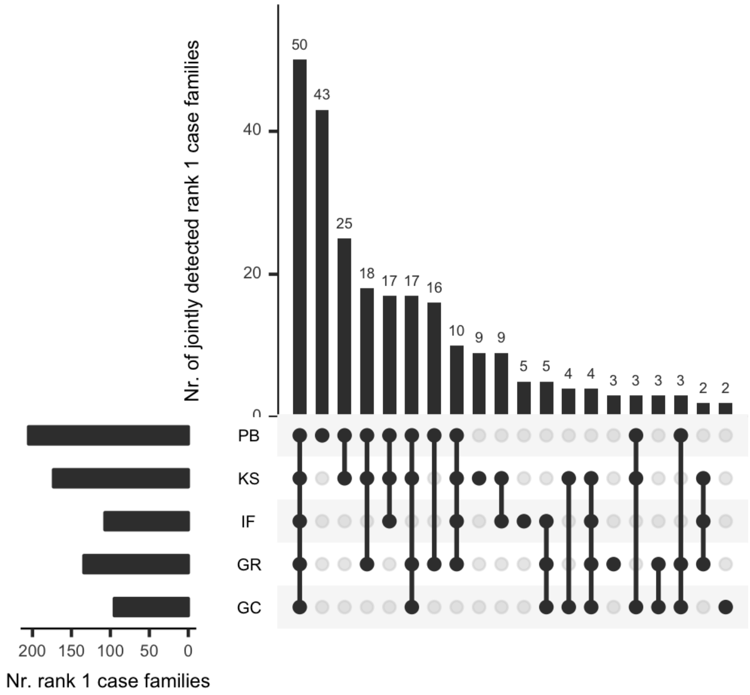

Supplementary Figure S63: Number of affected generations $G =$ all, penetrance $Q = 30\%$, prevalence $R = 2\%$. (See Figure 2 for a detailed description of the plot.)

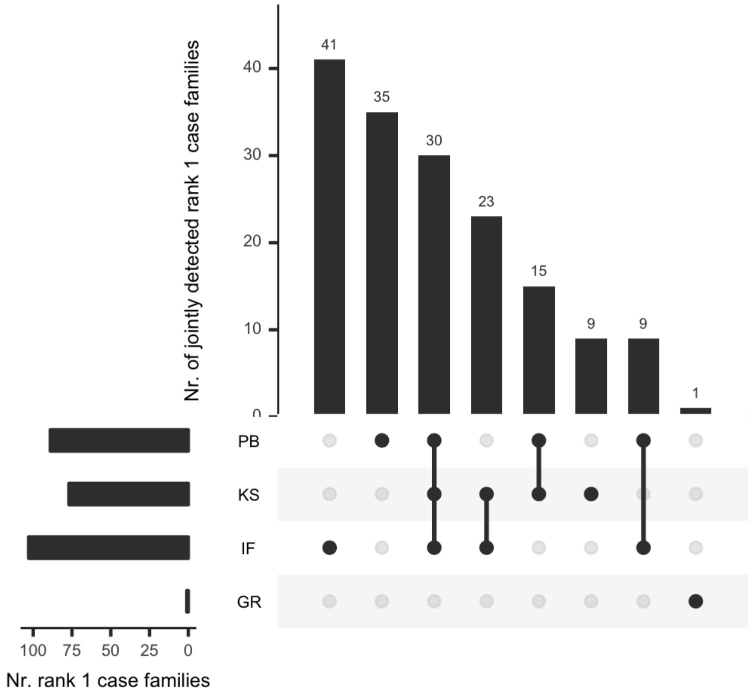

Supplementary Figure S64: Number of affected generations $G =$ all, penetrance $Q = 60\%$, prevalence $R = 10\%$. (See Figure 2 for a detailed description of the plot.)

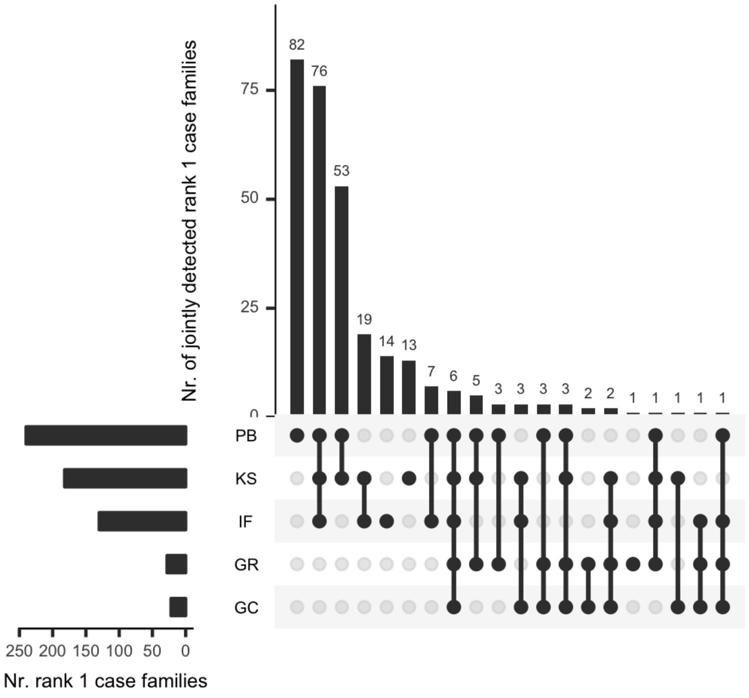

Supplementary Figure S65: Number of affected generations G = all, penetrance $Q = 60\%$, prevalence $R = 6.25\%$. (See Figure 2 for a detailed description of the plot.)

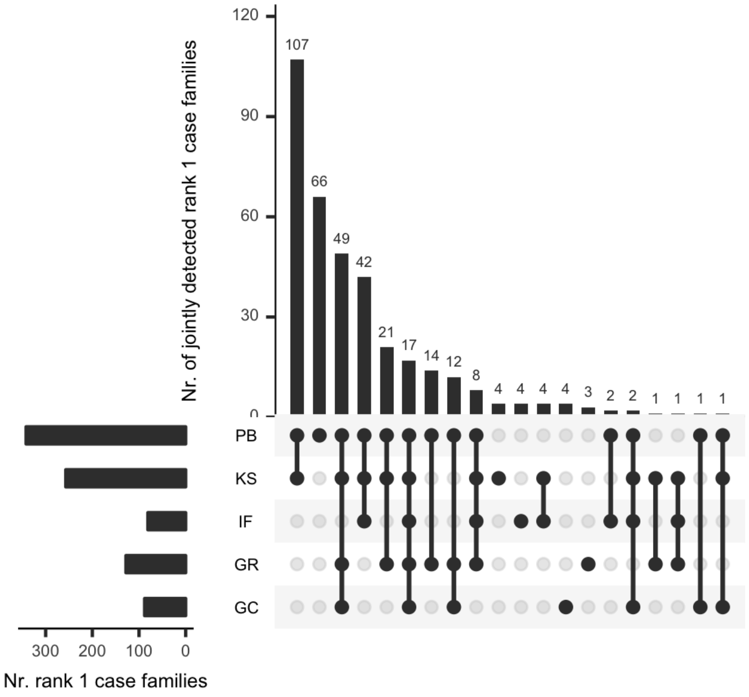

Supplementary Figure S66: Number of affected generations G = all, penetrance $Q = 60\%$, prevalence $R = 4\%$. (See Figure 2 for a detailed description of the plot.)

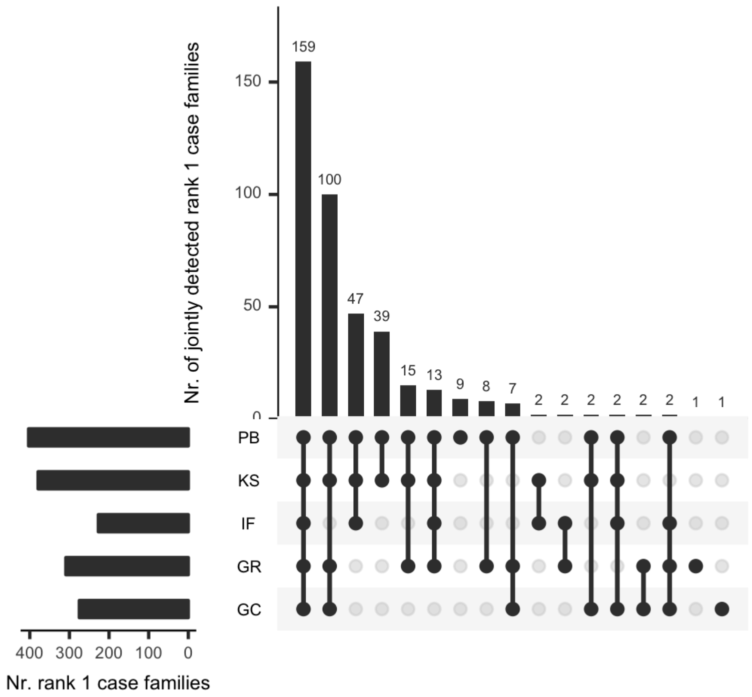

Supplementary Figure S67: Number of affected generations G = all, penetrance $Q = 60\%$, prevalence $R = 2\%$. (See Figure 2 for a detailed description of the plot.)

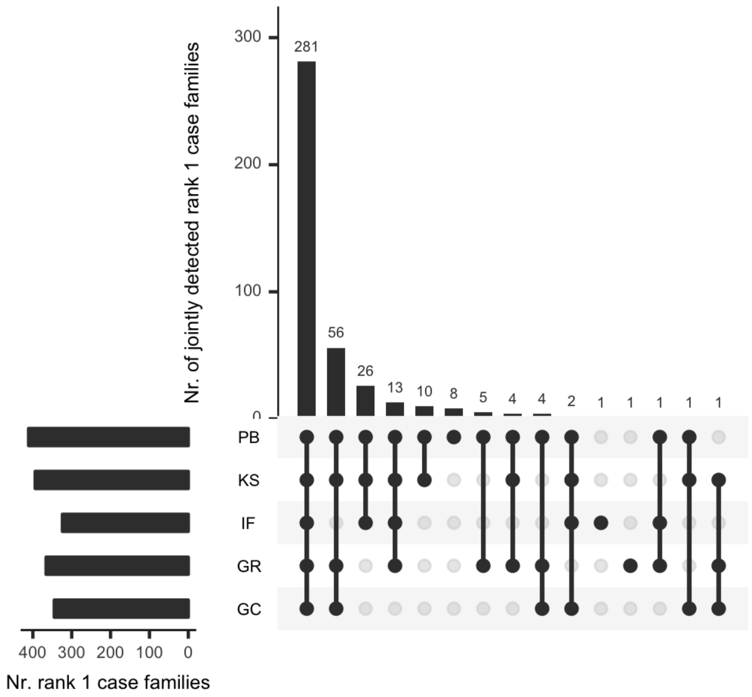

Supplementary Figure S68: Number of affected generations G = all, penetrance $Q = 60\%$, prevalence $R = 1.25\%$. (See Figure 2 for a detailed description of the plot.)

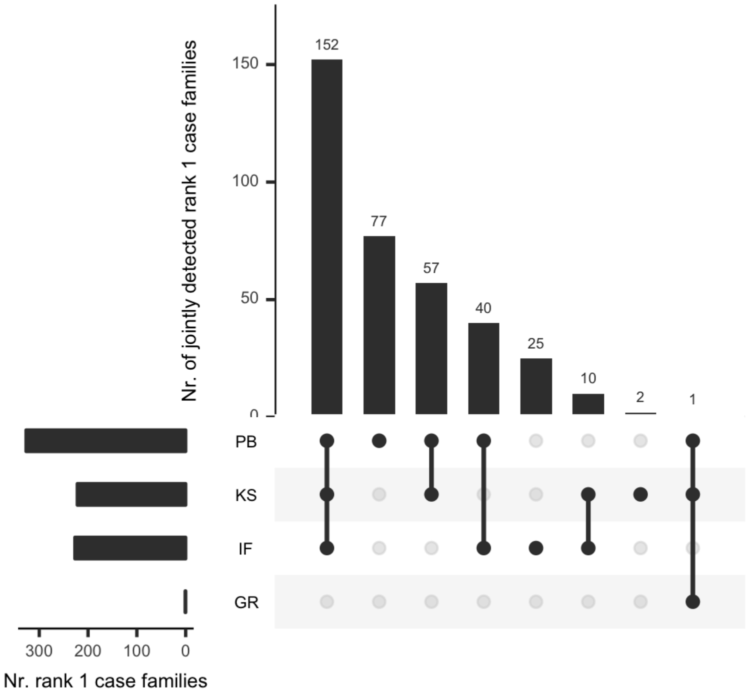

Supplementary Figure S69: Number of affected generations G = all, penetrance $Q = 100\%$, prevalence $R = 10\%$. (See Figure 2 for a detailed description of the plot.)

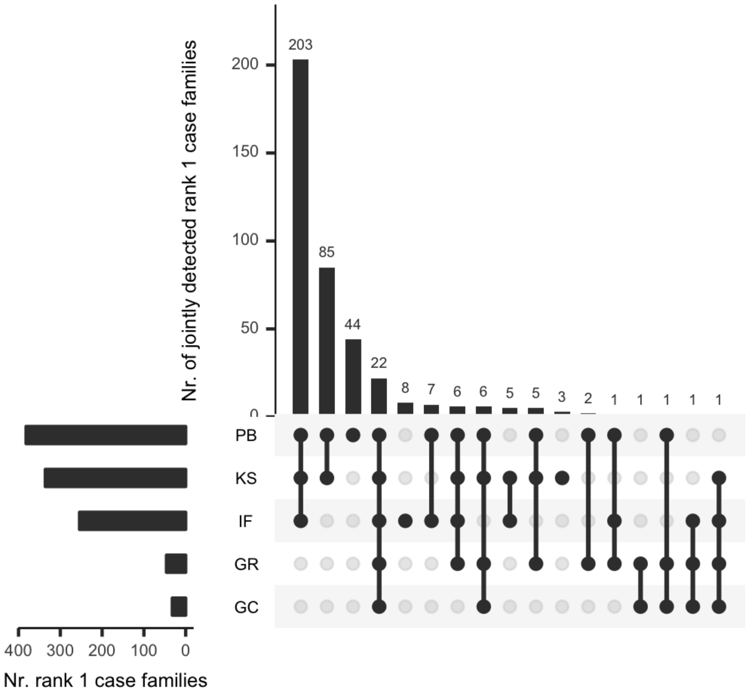

Supplementary Figure S70: Number of affected generations G = all, penetrance $Q = 100\%$, prevalence $R = 6.25\%$. (See Figure 2 for a detailed description of the plot.)

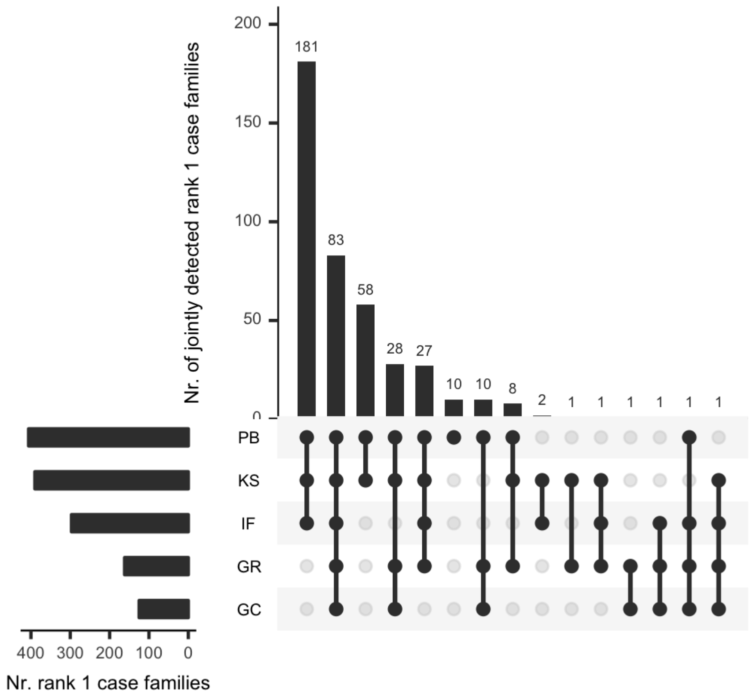

Supplementary Figure S71: Number of affected generations G = all, penetrance $Q = 100\%$, prevalence $R = 4\%$. (See Figure 2 for a detailed description of the plot.)

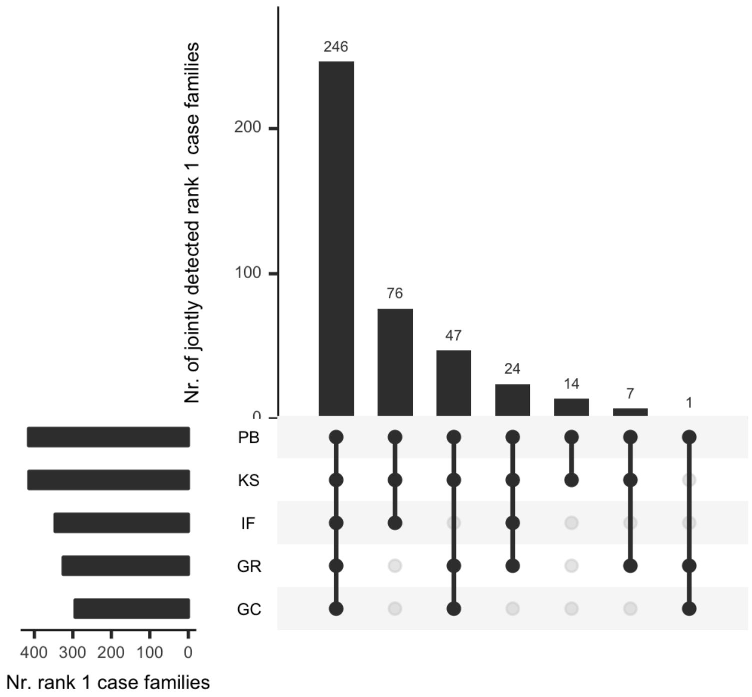

Supplementary Figure S72: Number of affected generations G = all, penetrance $Q = 100\%$, prevalence $R = 2\%$. (See Figure 2 for a detailed description of the plot.)

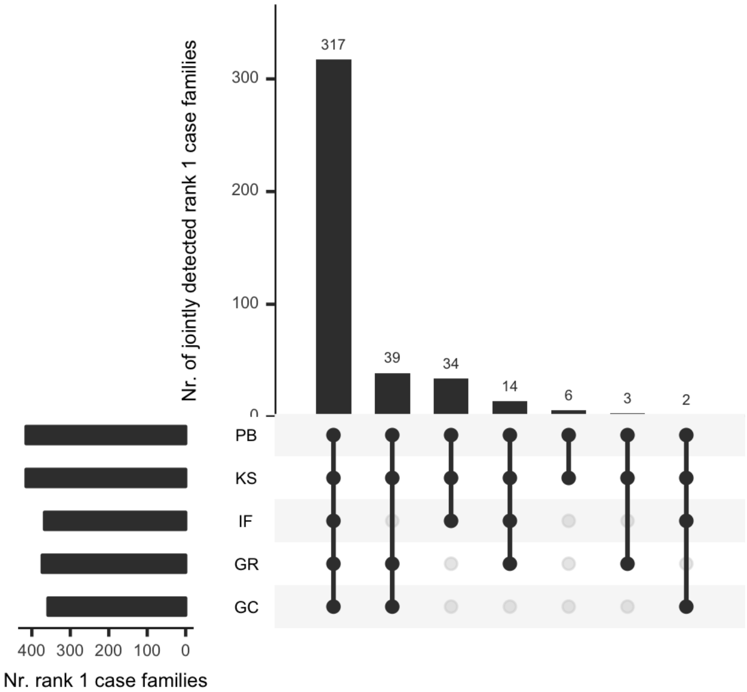

Supplementary Figure S73: Number of affected generations G = all, penetrance $Q = 100\%$, prevalence $R = 1.25\%$. (See Figure 2 for a detailed description of the plot.)

4 Binary classification performance evaluation

Putting the results from all 415 family sets into one single dataset allows test judgment by a binary classifier (via the P value threshold) for a fixed set of parameters G, Q, and R. This results in a very skewed dataset with 415 positives (cases) and 415×414=171810 controls. Receiver operating characteristic curves plot the true positive rate versus the false positive rate, which is an inappropriate means for a data set with an abundance of true negatives. Precision/recall plots are well suited for imbalanced data sets by putting emphasis on the detectability of positives and are used in Figure 3.

The following precision/recall plots represent curves that are parameterized on P_{adj}, with the lowest observed value associated with the leftmost point of the curve (i.e. recall=0.0). Possible parameter combinations are shown in Table 1.

In many cases, curves corresponding to tests GC and GR start with a precision value less than 1.0, because both cases and controls scored an identical, minimal P value. This happens during permutation testing phase, when no simulated constellation of affected family members produces a similar, or more extreme, test statistics as the one observed. Conversely, it may happen that $P_{adj} = 1.0$ for both cases and controls. From this point on, the curve becomes a straight line segment ending at point precision = 0 and recall = 1.0, as it is frequently visible for PB curves.

As an example, for GR test shown in Fig. 3b (corresponding to Supplementary Figure S87) we have 186 cases and 104 controls with identical, lowest P value. This results in a precision value of 186/(186+104) = 0.64 and a recall value of 186/415 = 0.45, which corresponds to the thin yellow line segment's vertical position and its length, respectively. In the same figure on the other hand, after 288 cases and 627 controls, the PB tests returns families only with $P_{adj} = 1.0$, such that the remaining 127 cases cannot be distinguished from the remaining 171 183 controls. The corresponding coordinate pair of (recall, precision) values is calculated as (0.69, 0.31), which is the point where PB test's black curve becomes a straight line ending in (1.0, 0.0).

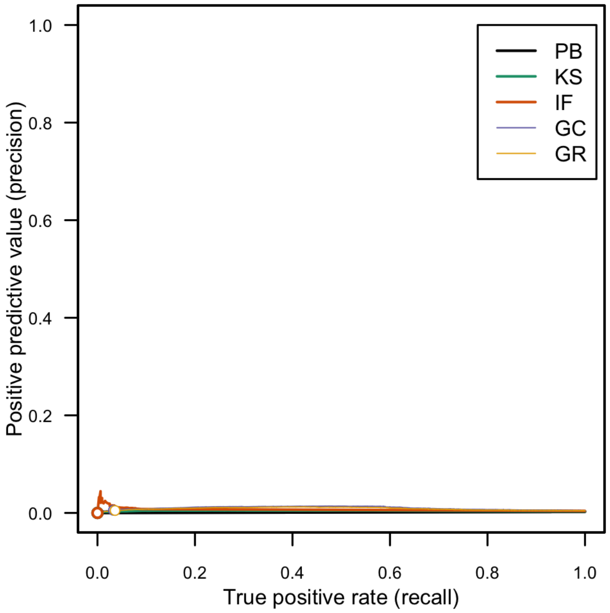

Supplementary Figure S74: Number of affected generations $G = 2$, penetrance $Q = 30\%$, prevalence $R = 4\%$. (See Figure 3 and the beginning or this section for a detailed description.)

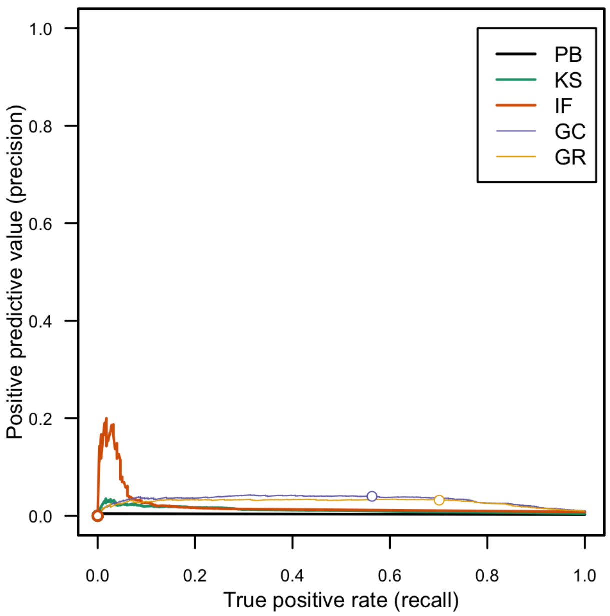

Supplementary Figure S75: Number of affected generations $G = 2$, penetrance $Q = 30\%$, prevalence $R = 2\%$. (See Figure 3 and the beginning or this section for a detailed description.)

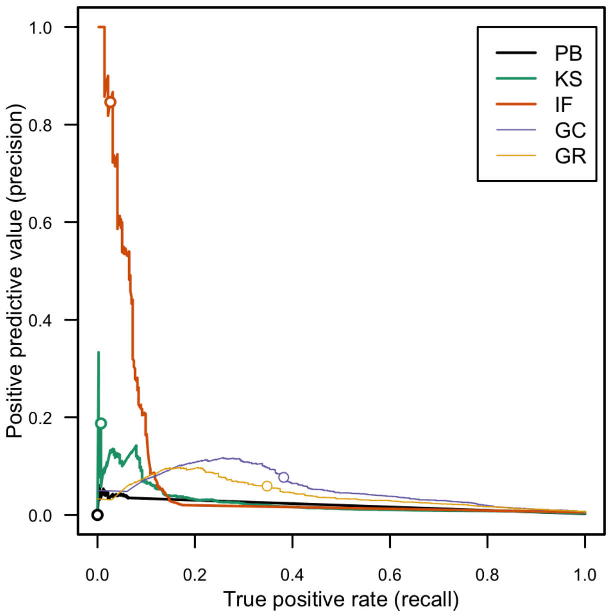

Supplementary Figure S76: Number of affected generations $G = 2$, penetrance $Q = 60\%$, prevalence $R = 4\%$. (See Figure 3 and the beginning or this section for a detailed description.)

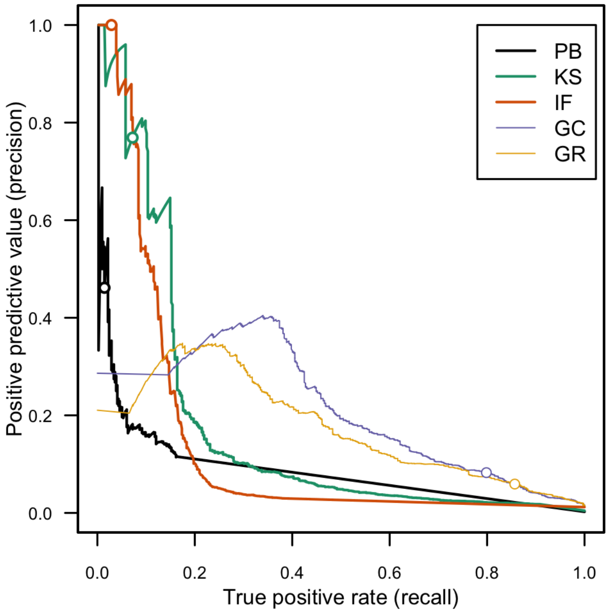

Supplementary Figure S77: Number of affected generations $G = 2$, penetrance $Q = 60\%$, prevalence $R = 2\%$. (See Figure 3 and the beginning or this section for a detailed description.)

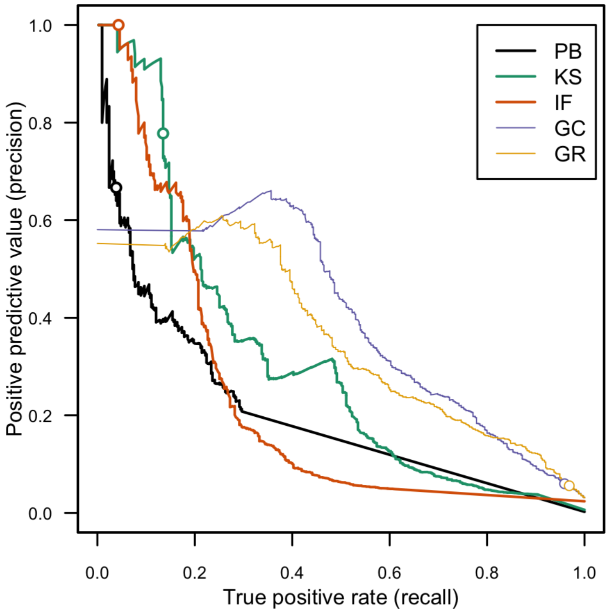

Supplementary Figure S78: Number of affected generations $G = 2$, penetrance $Q = 60\%$, prevalence $R = 1.25\%$. (See Figure 3 and the beginning or this section for a detailed description.)

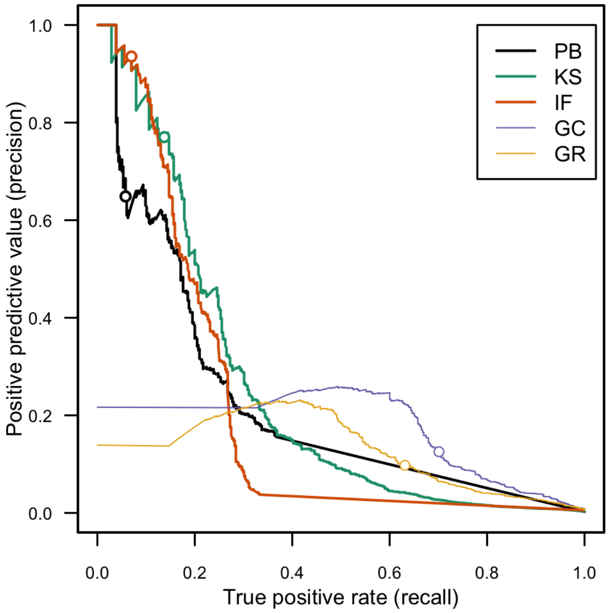

Supplementary Figure S79: Number of affected generations $G = 2$, penetrance $Q = 100\%$, prevalence $R = 4\%$. (See Figure 3 and the beginning or this section for a detailed description.)

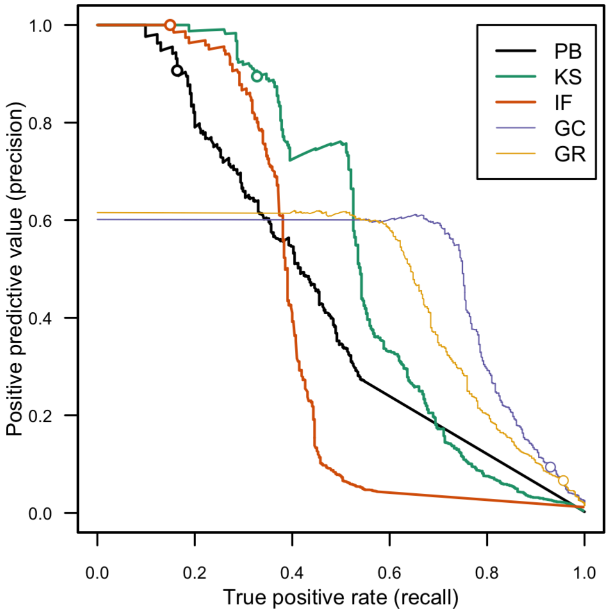

Supplementary Figure S80: Number of affected generations $G = 2$, penetrance $Q = 100\%$, prevalence $R = 2\%$. (See Figure 3 and the beginning or this section for a detailed description.)

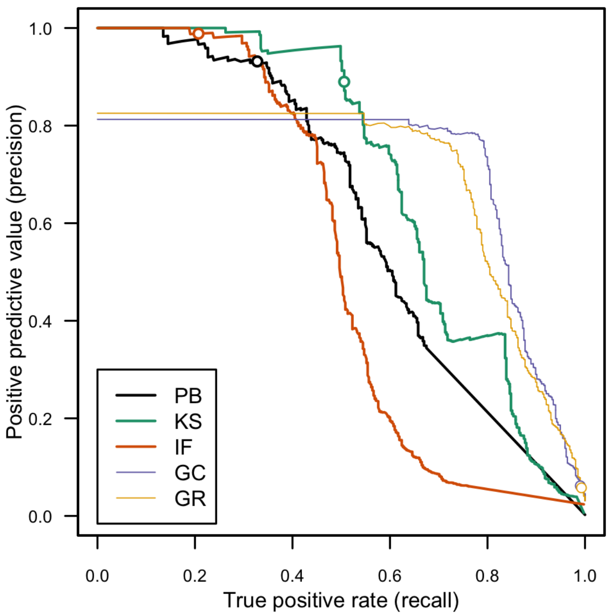

Supplementary Figure S81: Number of affected generations $G = 2$, penetrance $Q = 100\%$, prevalence $R = 1.25\%$. (See Figure 3 and the beginning or this section for a detailed description.)

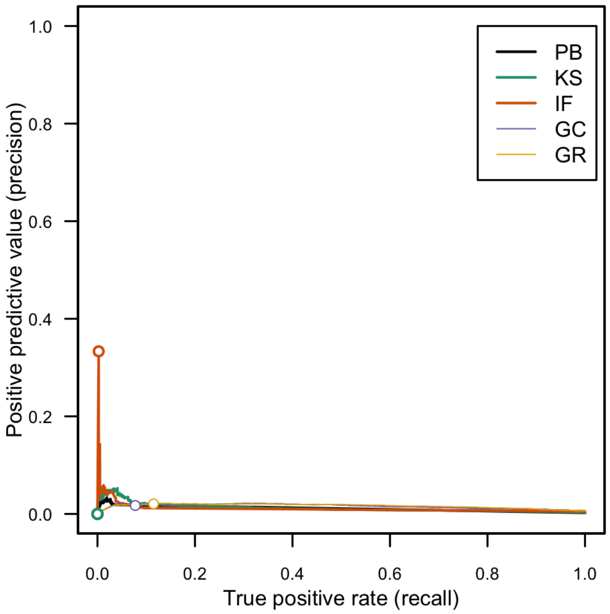

Supplementary Figure S82: Number of affected generations $G = 3$, penetrance $Q = 30\%$, prevalence $R = 4\%$. (See Figure 3 and the beginning or this section for a detailed description.)

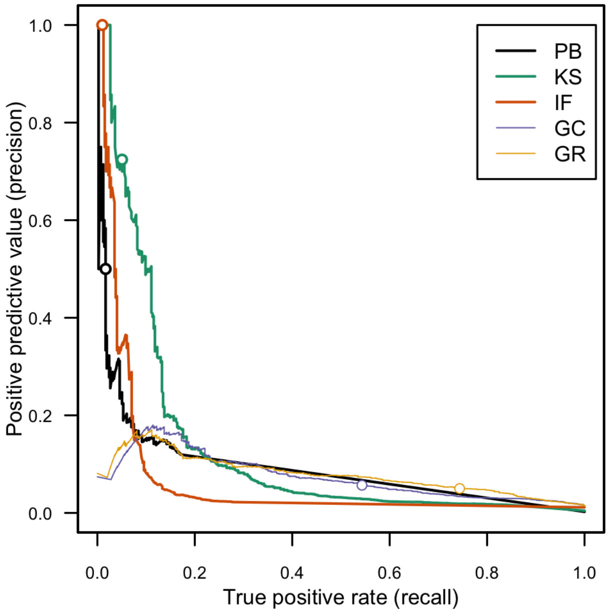

Supplementary Figure S83: Number of affected generations $G = 3$, penetrance $Q = 30\%$, prevalence $R = 2\%$. (See Figure 3 and the beginning or this section for a detailed description.)

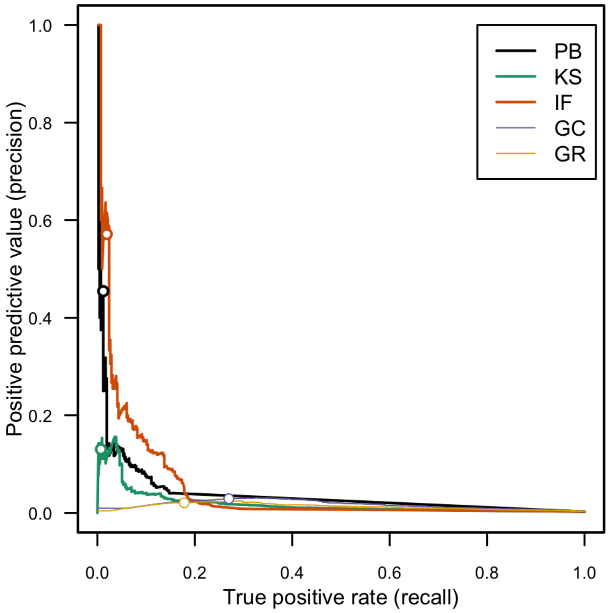

Supplementary Figure S84: Number of affected generations $G = 3$, penetrance $Q = 60\%$, prevalence $R = 10\%$. (See Figure 3 and the beginning or this section for a detailed description.)

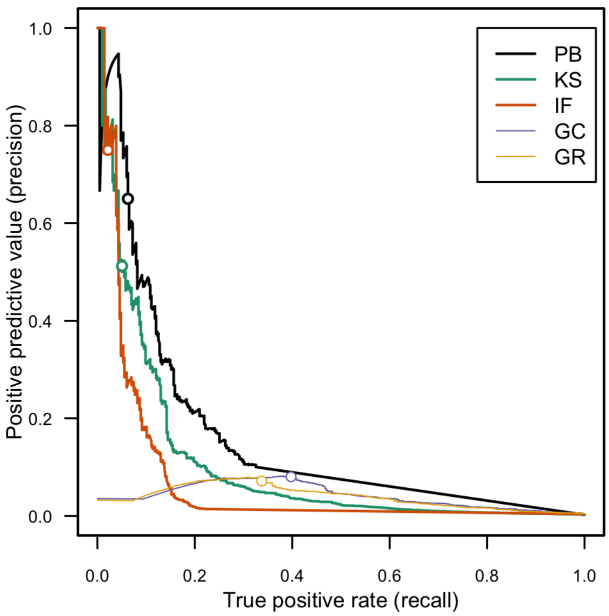

Supplementary Figure S85: Number of affected generations $G = 3$, penetrance $Q = 60\%$, prevalence $R = 6.25\%$. (See Figure 3 and the beginning or this section for a detailed description.)

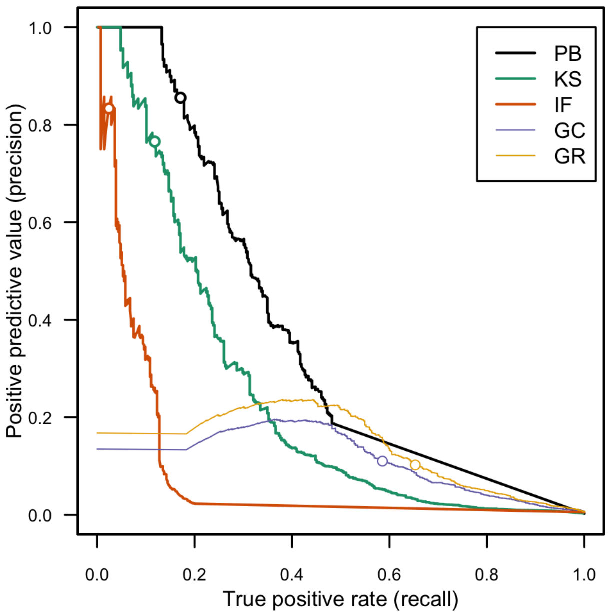

Supplementary Figure S86: Number of affected generations $G = 3$, penetrance $Q = 60\%$, prevalence $R = 4\%$. (See Figure 3 and the beginning or this section for a detailed description.)

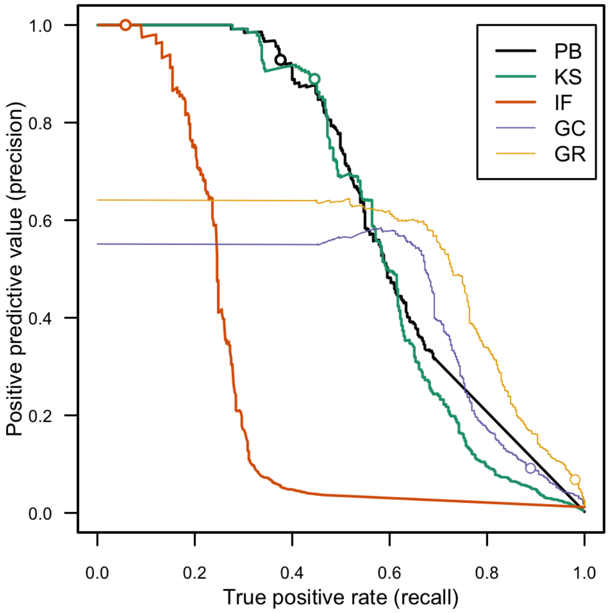

Supplementary Figure S87: Number of affected generations $G = 3$, penetrance $Q = 60\%$, prevalence $R = 2\%$. (See Figure 3 and the beginning or this section for a detailed description.)

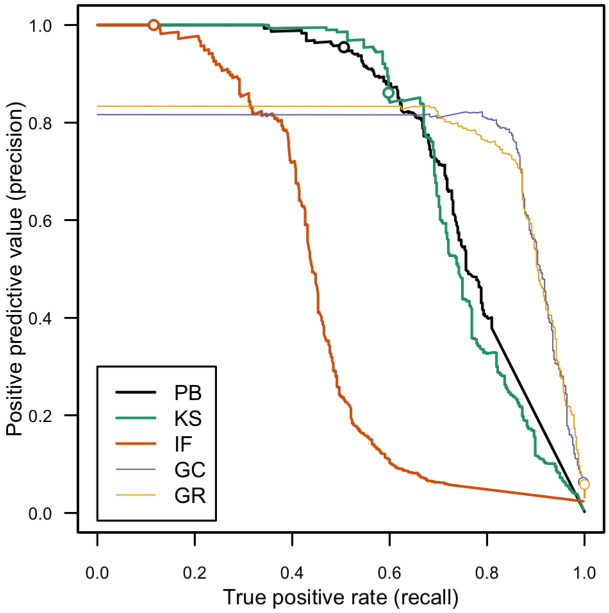

Supplementary Figure S88: Number of affected generations $G = 3$, penetrance $Q = 60\%$, prevalence $R = 1.25\%$. (See Figure 3 and the beginning or this section for a detailed description.)

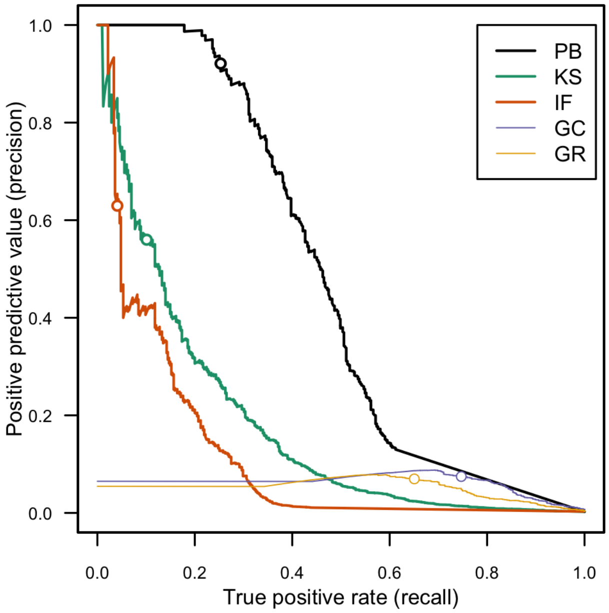

Supplementary Figure S89: Number of affected generations $G = 3$, penetrance $Q = 100\%$, prevalence $R = 10\%$. (See Figure 3 and the beginning or this section for a detailed description.)

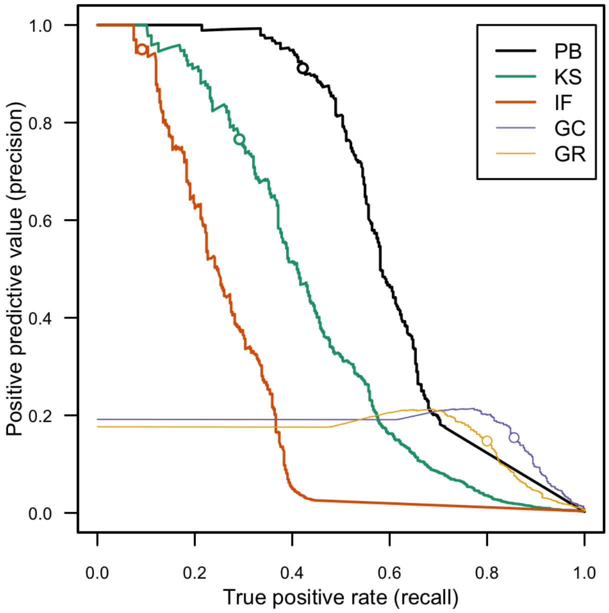

Supplementary Figure S90: Number of affected generations $G = 3$, penetrance $Q = 100\%$, prevalence $R = 6.25\%$. (See Figure 3 and the beginning or this section for a detailed description.)

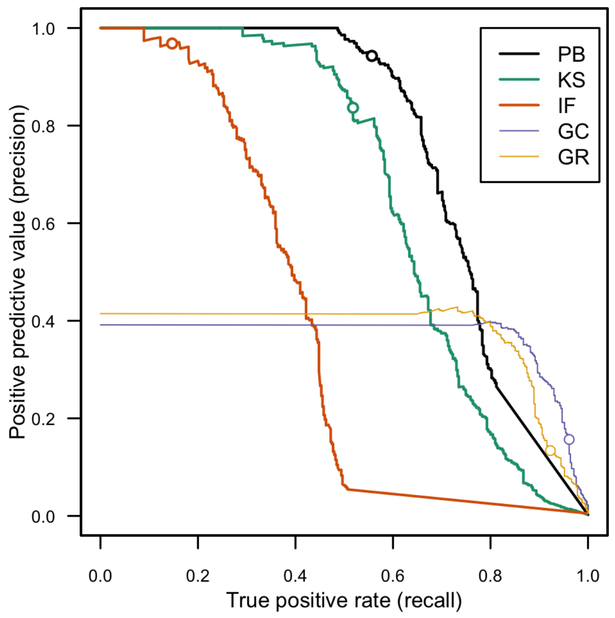

Supplementary Figure S91: Number of affected generations $G = 3$, penetrance $Q = 100\%$, prevalence $R = 4\%$. (See Figure 3 and the beginning or this section for a detailed description.)

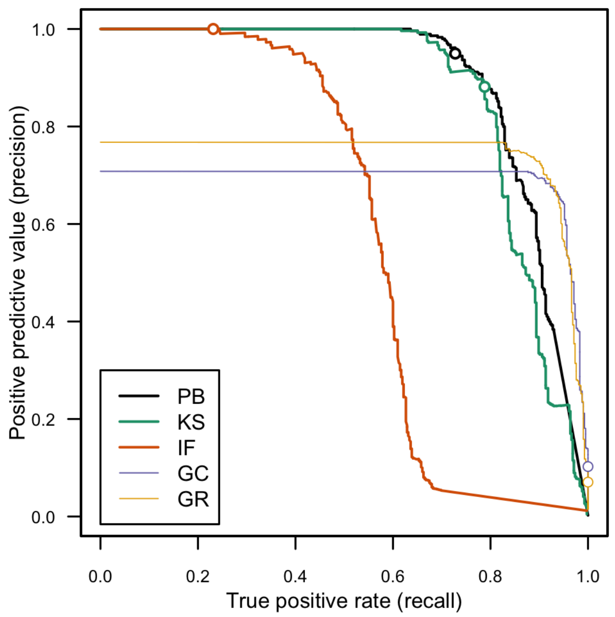

Supplementary Figure S92: Number of affected generations $G = 3$, penetrance $Q = 100\%$, prevalence $R = 2\%$. (See Figure 3 and the beginning or this section for a detailed description.)

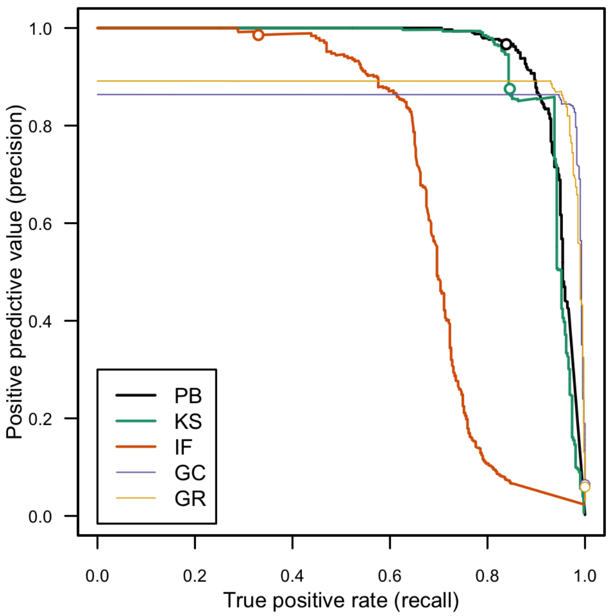

Supplementary Figure S93: Number of affected generations $G = 3$, penetrance $Q = 100\%$, prevalence $R = 1.25\%$. (See Figure 3 and the beginning or this section for a detailed description.)

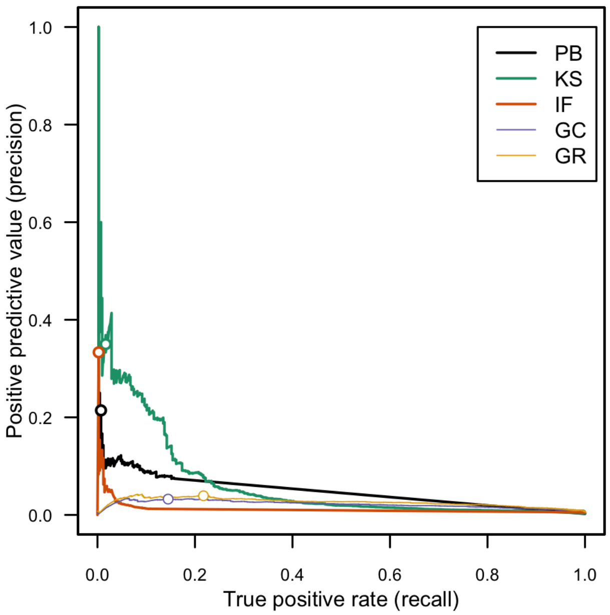

Supplementary Figure S94: Number of affected generations G = all, penetrance $Q = 30\%$, prevalence $R = 4\%$. (See Figure 3 and the beginning or this section for a detailed description.)

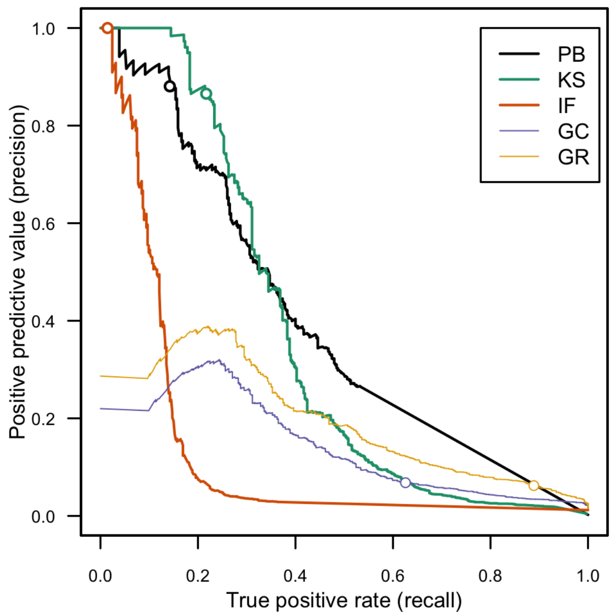

Supplementary Figure S95: Number of affected generations G = all, penetrance $Q = 30\%$, prevalence $R = 2\%$. (See Figure 3 and the beginning or this section for a detailed description.)

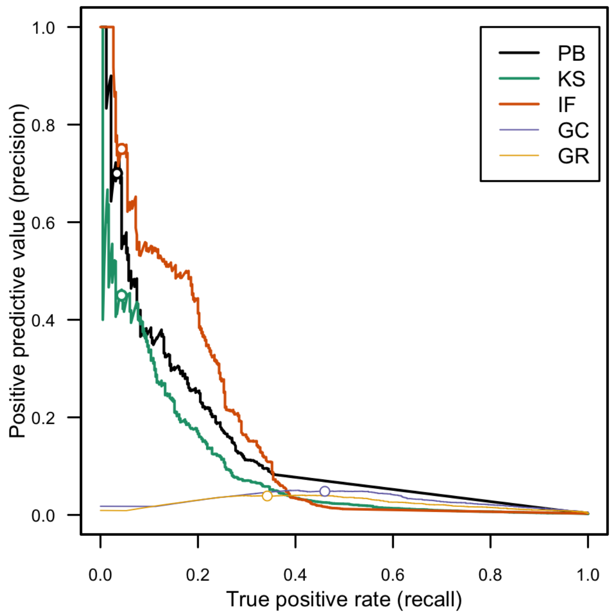

Supplementary Figure S96: Number of affected generations G = all, penetrance $Q = 60\%$, prevalence $R = 10\%$. (See Figure 3 and the beginning or this section for a detailed description.)

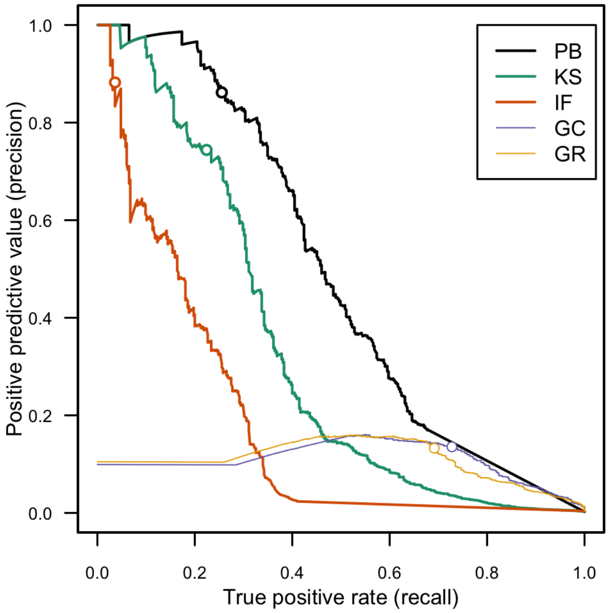

Supplementary Figure S97: Number of affected generations G = all, penetrance $Q = 60\%$, prevalence $R = 6.25\%$. (See Figure 3 and the beginning or this section for a detailed description.)

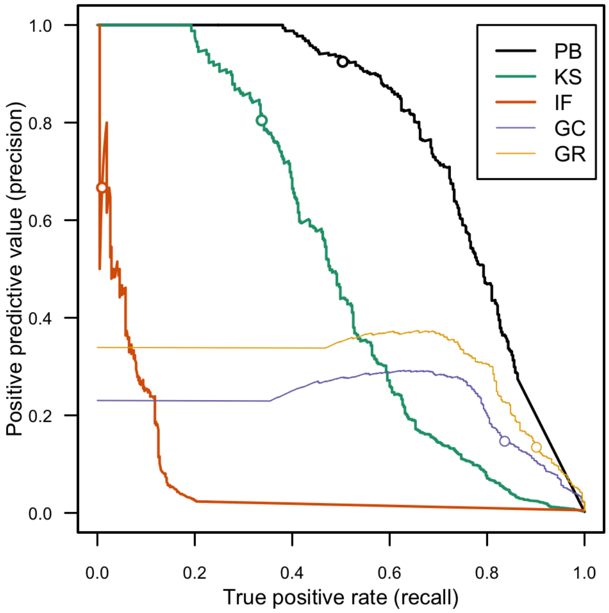

Supplementary Figure S98: Number of affected generations G = all, penetrance $Q = 60\%$, prevalence $R = 4\%$. (See Figure 3 and the beginning or this section for a detailed description.)

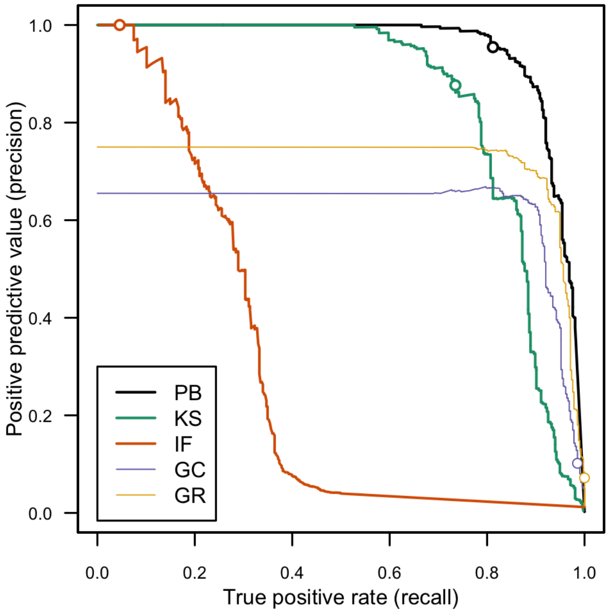

Supplementary Figure S99: Number of affected generations G = all, penetrance $Q = 60\%$, prevalence $R = 2\%$. (See Figure 3 and the beginning or this section for a detailed description.)

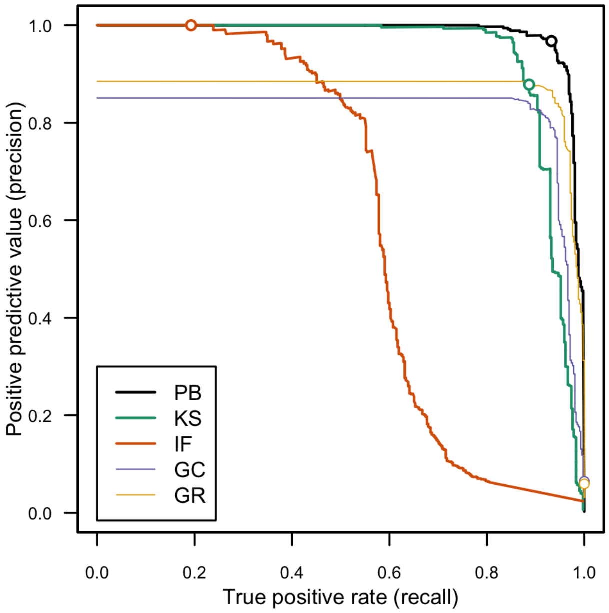

Supplementary Figure S100: Number of affected generations G = all, penetrance $Q = 60\%$, prevalence $R = 1.25\%$. (See Figure 3 and the beginning or this section for a detailed description.)

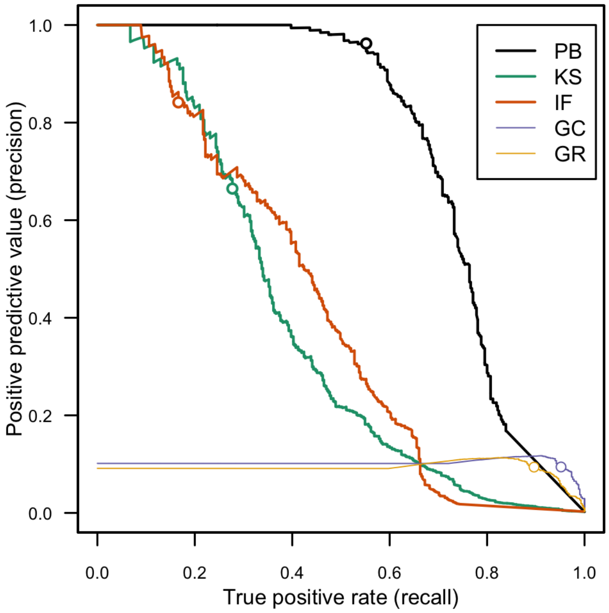

Supplementary Figure S101: Number of affected generations G = all, penetrance $Q = 100\%$, prevalence $R = 10\%$. (See Figure 3 and the beginning or this section for a detailed description.)

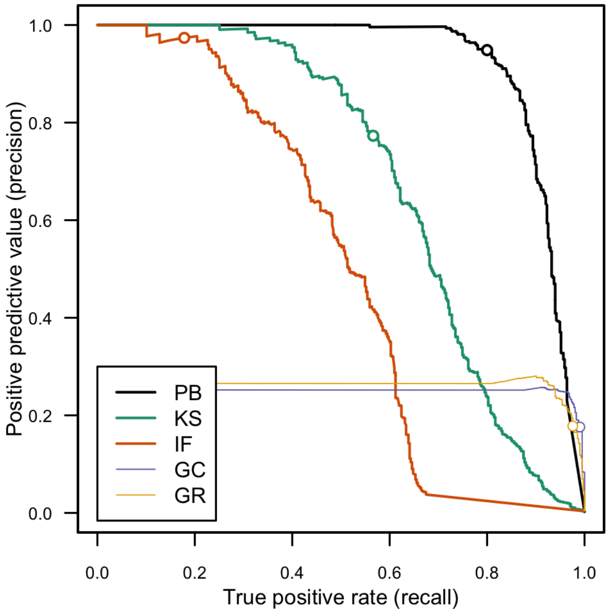

Supplementary Figure S102: Number of affected generations G = all, penetrance $Q = 100\%$, prevalence $R = 6.25\%$. (See Figure 3 and the beginning or this section for a detailed description.)

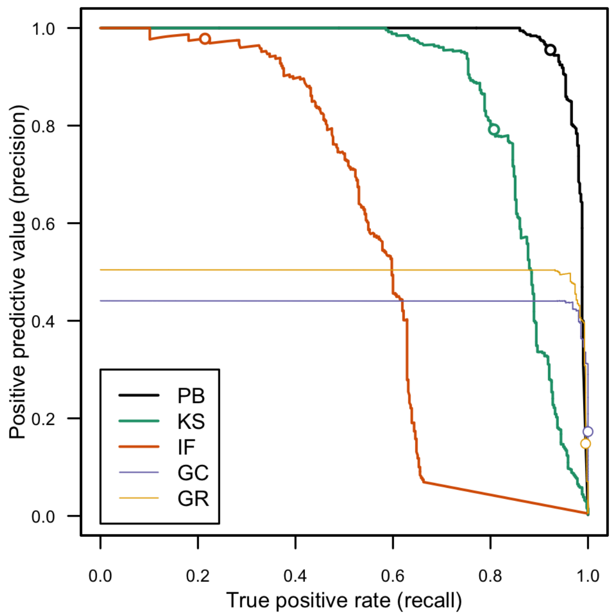

Supplementary Figure S103: Number of affected generations $G =$ all, penetrance $Q = 100\%$, prevalence $R = 4\%$. (See Figure 3 and the beginning or this section for a detailed description.)

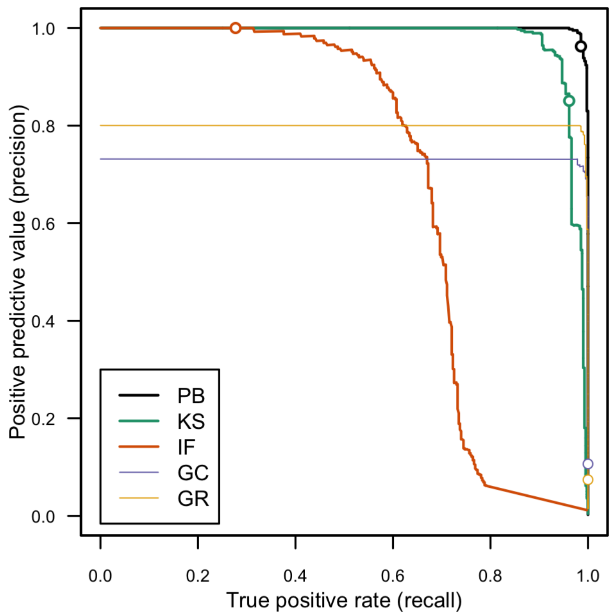

Supplementary Figure S104: Number of affected generations $G =$ all, penetrance $Q = 100\%$, prevalence $R = 2\%$. (See Figure 3 and the beginning or this section for a detailed description.)

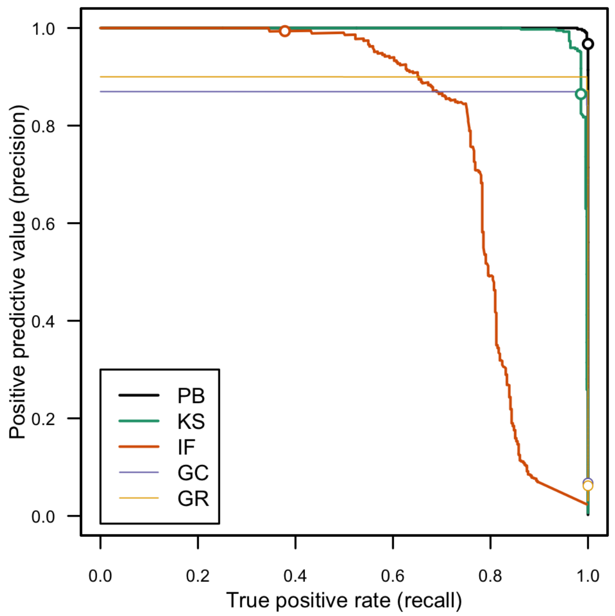

Supplementary Figure S105: Number of affected generations $G = \text{all}$, penetrance $Q = 100\%$, prevalence $R = 1.25\%$. (See Figure 3 and the beginning or this section for a detailed description.)

5 Investigation of familial cases of stroke in CHRIS

The CHRIS questionnaire specifically addresses stroke by several targeted questions following the stroke inquiry forms used by the Jackson Heart Study [2]. We have utilized the most general question "Have you ever been told by a doctor that you had a stroke?", which resulted in 54 cases of stroke out of a total of 4373 phenotyped participants. This yields a disease prevalence of 54/4373, i.e. $R = 1.23\%$, which is in line with reports from a study in the geographically close Italian Valle D'Aosta [3].

Within family XL, reported with $P_{adj}(\text{XL}) = 9.50 \times 10^{-4}$ by IF test, we have found a nuclear family consisting of an affected mother (age group 80s) and both of her sons (age group 60s) affected by stroke. All other kinship-based tests reported this occurrence of stroke as highly significant (KS, $P_{adj} = 5.55 \times 10^{-3}$; GR and GC, $P_{adj} < 10^{-5}$), whereas PB test reported $P_{adj} = 1.00$, since 49 cases occurred only in the large family.

The performance assessment provides a comparable scenario with parameters $G = 2$, $R = 1.25\%$, and $Q = 100\%$ (cf. Fig. 1d and Supplementary Figures S17, S49, and S81). The assessment indicates a high precision value of 99% for KS at the P_{adj} value observed for this stroke case in the CHRIS cohort. Even the overestimating GR and GC tests perform well with precision values higher than 80% when selecting their respective P_{adj} values as thresholds, corroborating the presence of a familial case of stroke in these CHRIS individuals.

References

1. Conway, J. R., Lex, A. & Gehlenborg, N. UpSetR: an R package for the visualization of intersecting sets and their properties. *Bioinformatics* **33,** 2938–2940 (Sept. 2017).
2. Sempos, C. T., Bild, D. E. & Manolio, T. A. Overview of the Jackson Heart Study: a study of cardiovascular diseases in African American men and women. *The American journal of the medical sciences* **317,** 142–146 (Mar. 1999).
3. D'Alessandro, G. *et al.* Prevalence of stroke and stroke-related disability in Valle d'Aosta, Italy. *Neurological sciences : official journal of the Italian Neurological Society and of the Italian Society of Clinical Neurophysiology* **31,** 137–141 (Apr. 2010).
